# Hypernetworks induce stable hyperlocking

**DOI:** 10.1038/s41467-026-74556-1

**Published:** 2026-06-24

**Authors:** Eddie Nijholt, Tiago Pereira, Matthias Wolfrum, Sagnik Chakraborty, István Z. Kiss, Jürgen Kurths

**Affiliations:** 1 Institute of Mathematical Sciences and Computation, University of São Paulo, São Paulo, Brazil; 2 Department of Mathematics, Imperial College London, London, UK; 3 Weierstrass Institute for Applied Analysis and Stochastics, Berlin, Germany; 4 Department of Chemistry, Saint Louis University, St. Louis, MO USA; 5 Potsdam Institute for Climate Impact Research, Potsdam, Germany

**Keywords:** Applied mathematics, Complex networks, Nonlinear phenomena

## Abstract

Hypernetworks capture coupling structures where interactions extend beyond pairs to groups of three or more units, called hyperedges. They are of increasing importance for many systems such as the brain, social groups, ecosystems, and the climate. We describe here a synchronization phenomenon that is distinctive for hypernetworks. We uncover that in a system of three coupled oscillators with resonant frequencies, the coupling by a triadic hypernetwork motif, where a third node modulates the interaction between two others, can induce a stable locking of a phase triplet, while no pairwise locking is observed. Using normal form transformations and phase reduction, we derive analytically how a specific choice of the coupling functions induces this hyperlocking. We confirm our predictions with both numerical simulations and chemical experiments. Our findings uncover a new synchronization mechanism intrinsic to higher-order interactions and open new directions for controlling real-world complex dynamics beyond pairwise frameworks.

## Abstract (web summary)

Higher-order interactions, such as those found in hypernetworks, are increasingly recognized for their significance in complex systems like the brain and ecosystems, where traditional pairwise interaction models may fall short. This study reveals a synchronization phenomenon termed “hyperlocking," which occurs in hypernetworks, demonstrating the emergence of a stable phase locking with higher-order relations between the frequencies.

## Introduction

Many natural and engineered systems, from the brain to social groups, ecosystems, and the climate, are composed of interacting elements^1^. A standard modeling assumption for such systems is that interactions happen between pairs of elements^2–5^. While this pairwise modeling has been successful, recent work has shown that, in many applications, interactions between more than two elements cannot be decomposed as a sum of pairwise interactions^6–9^. These interactions between three or more elements are known as higher-order interactions.

**Fig. 1:**
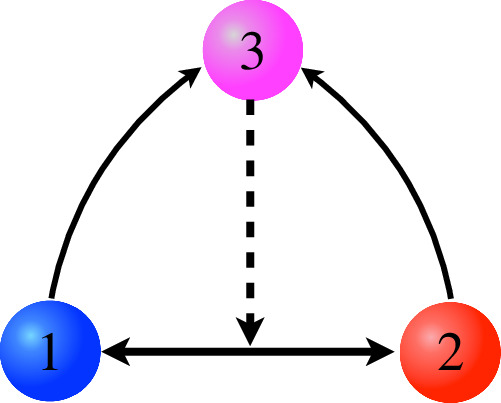
Triadic hypernetwork motif. Pairwise interactions (black arrows) and non-pairwise interaction: node 3 influences the coupling between nodes 1 and 2 (dashed arrow). Together, this establishes a hyperedge including all three nodes and acting on nodes 1 and 2 in a symmetric way.

Higher-order interactions encompass a broad class of mechanisms, including symmetric interactions, such as simplicial complexes, as well as asymmetric motifs in which one element modulates the interactions between others^6,10^. Here, we consider the latter case and focus on triadic interactions, where one node regulates or mediates the coupling between two others, rather than participating symmetrically in a group interaction. We depict this situation in Fig. 1.

Such triadic interactions appear in a wide range of systems. For example, in the brain, a triadic interaction appears when communication between two regions depends on the activity of a third one^11^. Similarly, in ecological communities, the interaction between two species can change in the presence of a third^12^. In climate systems, extreme compound events such as simultaneous heatwaves and droughts cannot be explained by pairwise statistics alone^13^. Similarly, the interaction between tipping elements, such as ice sheets and ocean circulation, is often mediated by the state of a third component^14^. In social dynamics, individuals typically require reinforcement from multiple peers to adopt a new behavior, reflecting a dependence on simultaneous rather than cumulative influence^15^.

Despite their growing relevance, the dynamical role of higher-order interactions remains elusive. Only recently have mathematical frameworks such as hypergraphs and simplicial complexes provided a direct way to represent and analyse these effects^7,16,17^. Parallel advances have shown that higher-order interactions can also be reliably recovered from data^18–20^. These developments reveal how higher-order coupling can reshape collective behavior^21,22^, alter stability^23^, shift the onset of synchronization^24–26^, and slow loss of synchrony under parameter changes^27^.

In this work, we demonstrate a new synchronization phenomenon that arises in networks with triadic interactions and has no analog in systems with purely pairwise coupling. We show that, when all oscillators have nearly identical natural frequencies, the triadic motif shown in Fig. 1 admits open sets of coupling functions for which the dynamics converge to a stable triplet-locked state that cannot be decomposed into combinations of pairwise phase-locking relations. Remarkably, despite the fact that all oscillator pairs are themselves frequency-resonant, pairwise phase synchronization does not occur. We call this phenomenon hyperlocking: a form of synchronization sustained exclusively by higher-order interactions, for which coherence is achieved only through a genuine triadic phase relation.

We develop a theory, based on phase reduction and normal form transformations, that explains how this behavior emerges. The framework is constructive: it allows us to design coupling functions that realize desired hypernetwork states, thus offering control over collective dynamics. We support our theoretical predictions with both numerical simulations and chemical experiments. Our findings show that triadic interactions can give rise to fundamentally new dynamical mechanisms, and suggest that they may play a key role in shaping collective behavior in real-world systems.

## Results

### Triadic network motif

The interaction structure we consider is depicted in Fig. 1. It corresponds to a system of coupled oscillators of the form

1 $$\dot{z}_{1} 	={f}_{1}({z}_{1})+\alpha g({z}_{3})h({z}_{2},{z}_{1}) \\ \dot{z}_{2} 	={f}_{2}({z}_{2})+\alpha g({z}_{3})h({z}_{1},{z}_{2}) \\ \dot{z}_{3} 	={f}_{3}({z}_{3})+\alpha h ({z}_{1},{z}_{3})+\alpha h({z}_{2},{z}_{3}),$$

with *z*_*j*_,  *j* ∈ {1, 2, 3}, being the dynamical variables for the oscillators and $\alpha \in {\mathbb{R}}$ the coupling strength. For *α* = 0, i.e., without coupling, each oscillator is assumed to have a stable periodic oscillation with natural frequency *ω*_*j*_. Eq. (1) captures the higher-order nature of Fig. 1 in the following way. The dyadic coupling function *h* establishes a classical network structure of pairwise interactions between the nodes, indicated in Fig. 1 by the black arrows. The hypernetwork nature of our motif is induced by the dashed arrow corresponding to the function *g*. It indicates that node 3 influences the dyadic couplings between nodes 1 and 2, and *g*, together with *h*, induces a hyperlink among the three nodes. As we will show below, in a situation close to full resonance, i.e., when each frequency is distinct but is near a common frequency, this type of higher-order coupling can give rise to hyperlocking.

### Effective frequencies and hyperlocking

Since the oscillators have stable periodic orbits, the oscillatory behavior persists for small couplings 0 < *α* ≪ 1, however, their frequencies can change because of the interaction and develop an effective frequency. To obtain the effective frequency for each oscillator, we first introduce the phase ${\phi }_{i}(t)={\phi }_{i}({z}_{i}(t))\in {S}^{1}={\mathbb{R}}/2\pi {\mathbb{Z}}$ and the unwrapped phase ${\widehat{\phi }}_{i}(t)\in {\mathbb{R}}$. The effective frequency is then given by

$${\Omega }_{i}={{\rm{lim}}}_{t\to \infty }\frac{1}{t}{\widehat{\phi }}_{i}(t).$$

Clearly, it depends on the natural frequencies *ω*_*j*_, on the network structure, and on the coupling functions.

#### Pairwise and triplet locking

In networks, one expects to have a phase locking between pairs of connected nodes whenever their natural frequencies are near a resonance *nω*_*i*_ + *mω*_*j*_ ≈ 0 for some integers *n*, *m*. This means that the effective frequencies exactly satisfy

2 $$n{\Omega }_{i}+m{\Omega }_{j}=0,$$

which corresponds to the fact that $n{\widehat{\phi }}_{i}(t)+m{\widehat{\phi }}_{j}(t)$ stay bounded. Similarly, one can define triplet locking^28^, where for three integers (*ℓ*, *n*, *m*) we have

3 $$\ell {\Omega }_{i}+n{\Omega }_{j}+m{\Omega }_{k}=0.$$

Classical theory shows that close to a triplet resonance of the natural frequencies

$$\ell {\omega }_{1}+n{\omega }_{2}+m{\omega }_{3}={{\mathcal{O}}}({\alpha }^{2})$$

a generic pairwise coupling typically induces a corresponding triplet locking (3). Such a triplet resonance typically comes without any additional pairwise resonances and, hence, no simultaneous pairwise locking is expected. This is completely different in the case of full resonance, where all three frequencies are nearly identical. In this case, all pairs of frequencies are resonant and pairwise locking (2), where one or several of the pairs (*ℓ*, *n*), (*n*, *m*), (*m*, *ℓ*) are equal to (1, −1), could emerge and, at the same time, non-trivial triplets as, e.g., (*ℓ*, *n*, *m*) = (1, 1, −2) are resonant and could induce a corresponding triplet locking (3). Note that at full resonance, triplet locking may also arise as a direct consequence of two pairwise lockings. For a triplet with effective frequencies (*Ω*_1_, *Ω*_2_, *Ω*_3_), if *Ω*_1_ = *Ω*_2_ and *Ω*_2_ = *Ω*_3_, then the triplet relation

$${\Omega }_{1}+{\Omega }_{2}-2{\Omega }_{3}=0$$

is satisfied. In fact, two pairwise resonances

$$n{\Omega }_{1}=m{\Omega }_{2}\,\,{{\rm{and}}}\,\,r{\Omega }_{2}=s{\Omega }_{3}$$

imply not only a third pairwise resonance *nrΩ*_1_ = *smΩ*_3_, but also a triplet resonance

$$n{\Omega }_{1}+s{\Omega }_{3}=(m+r){\Omega }_{2}.$$

Here, the triplet relations are induced by pairs.

We define *hyperlocking* as a situation where, close to a full resonance, a stable triplet locking arises, while none of the available pairwise resonances induces a corresponding pairwise locking. This means in particular that a triplet locking arising at a triplet resonance we do not designate as hyperlocking, since pairwise locking is excluded a priori by the frequency detuning.

Our main result is that, in the hypernetwork motif, robust hyperlocking occurs for proper choices of the coupling function. The synergy between the coupling function and triadic motif generates a collective phase relation that cannot be decomposed into pairwise locking.

#### Networks versus triadic motif

Notice that if we set *g*(*z*) = 1, this is equivalent to removing the dashed link from node 3, in Fig. 1. This reduces the triadic motif to a classical network of pairwise coupled oscillators. Then, classical synchronization theory shows that if the natural frequencies are not close to full resonance, but instead satisfy a triplet relation such as *ω*_1_−2*ω*_3_ + *ω*_2_ ≈ 0, then triplet locking may occur^28,29^. However, near full resonance *ω*_1_ ≈ *ω*_2_ ≈ *ω*_3_, triplet locking appears as a byproduct of pairwise locking generically. Indeed, for networks, the effective dynamics is dominated by pairwise interactions, as we show in Supplementary Note I. This is a fundamental feature of networks: when interactions are pairwise, and nodes are near full resonance, the effective reduced dynamics remain pairwise, generically.

### Hyperlocking near a Hopf bifurcation

We choose Stuart-Landau oscillators

4 $${f}_{j}(z)=(\lambda+i{\omega }_{j})z-| z{| }^{2}z,$$

where *z* is a complex number, with *j* = 1, 2, 3, and $\lambda \in {\mathbb{R}}$ provides the radius of a stable periodic orbit ${z}_{j}(t)=\sqrt{\lambda }{e}^{i{\omega }_{j}t}$ with frequency *ω*_*j*_ emerges for positive values of *λ*. We can achieve the hyperlocking behavior by choosing linear coupling functions

5 $$h(z,w) 	=w-\overline{z} \\ g(u) 	=u\,.$$

**Fig. 2:**
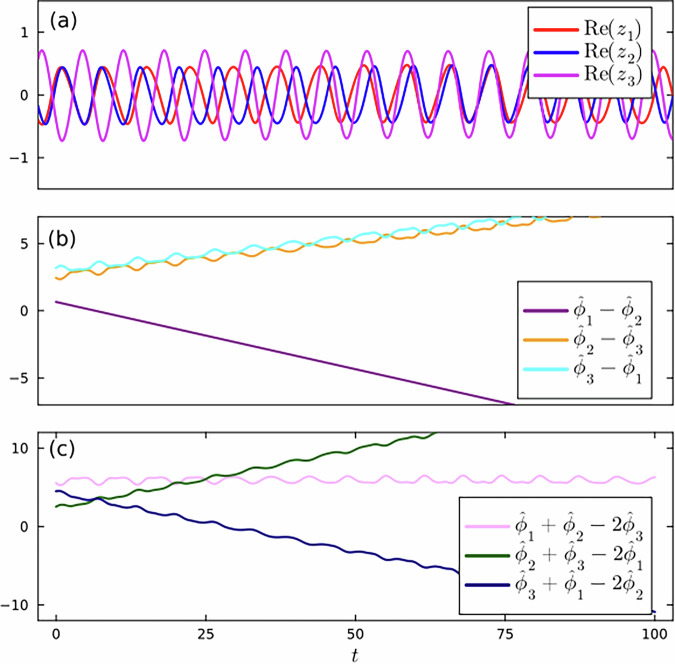
Dynamics of the triadic network. Numerical solution of system (1)–(5). **a** Shows the time traces of the real parts of *z*_1_, *z*_2_, and *z*_3_. **b** Shows the pairwise phase differences and reveals that no phase locking (where the pairwise phase difference is bounded) is observed. **c** Shows the triplet phase differences. The triplet combination *ϕ*_1_ + *ϕ*_2_−2*ϕ*_3_ (blue) remains bounded, indicating the existence of a hyperlocking between this triplet of phases. Parameters: *ω*_1_ = 0.95, *ω*_2_ = 1.05, *ω*_3_ = 0.98, *λ* = 0.2, *α* = 0.15.

We choose similar frequencies *ω*_1_ = 0.95, *ω*_2_ = 1.05 and *ω*_3_ = 0.98 together with a small coupling *α* and solve system (1) numerically using Julia^30^. In Fig. 2a, we show the time traces of the real parts ${{\rm{Re}}}({z}_{j}(t))$. These coupling functions *g* and *h* were not chosen ad hoc. They are designed from our normal form approach so that our triadic network displays hyperlocking.

We observe nearly sinusoidal oscillations with amplitude $\sqrt{\lambda }\approx 0.55$. To see the interaction of their phases, we perform a polar decomposition ${z}_{i}(t)={r}_{j}{e}^{i{\phi }_{j}}$ and obtain the unwrapped phases ${\widehat{\phi }}_{j}$. In Fig. 2b, we plot the pairwise phase differences between ${\widehat{\phi }}_{i}-{\widehat{\phi }}_{j}$ for all pairs *i* ≠ *j* and notice that the differences grow in time, indicating the absence of pairwise frequency locking. However, as seen in Fig. 2c, there is hyperlocking for (*ℓ*, *m*, *n*) = (1, 1, −2), since the corresponding triplet phase ${\widehat{\phi }}_{1}+{\widehat{\phi }}_{2}-2{\widehat{\phi }}_{3}$ stays bounded.

**Fig. 3:**
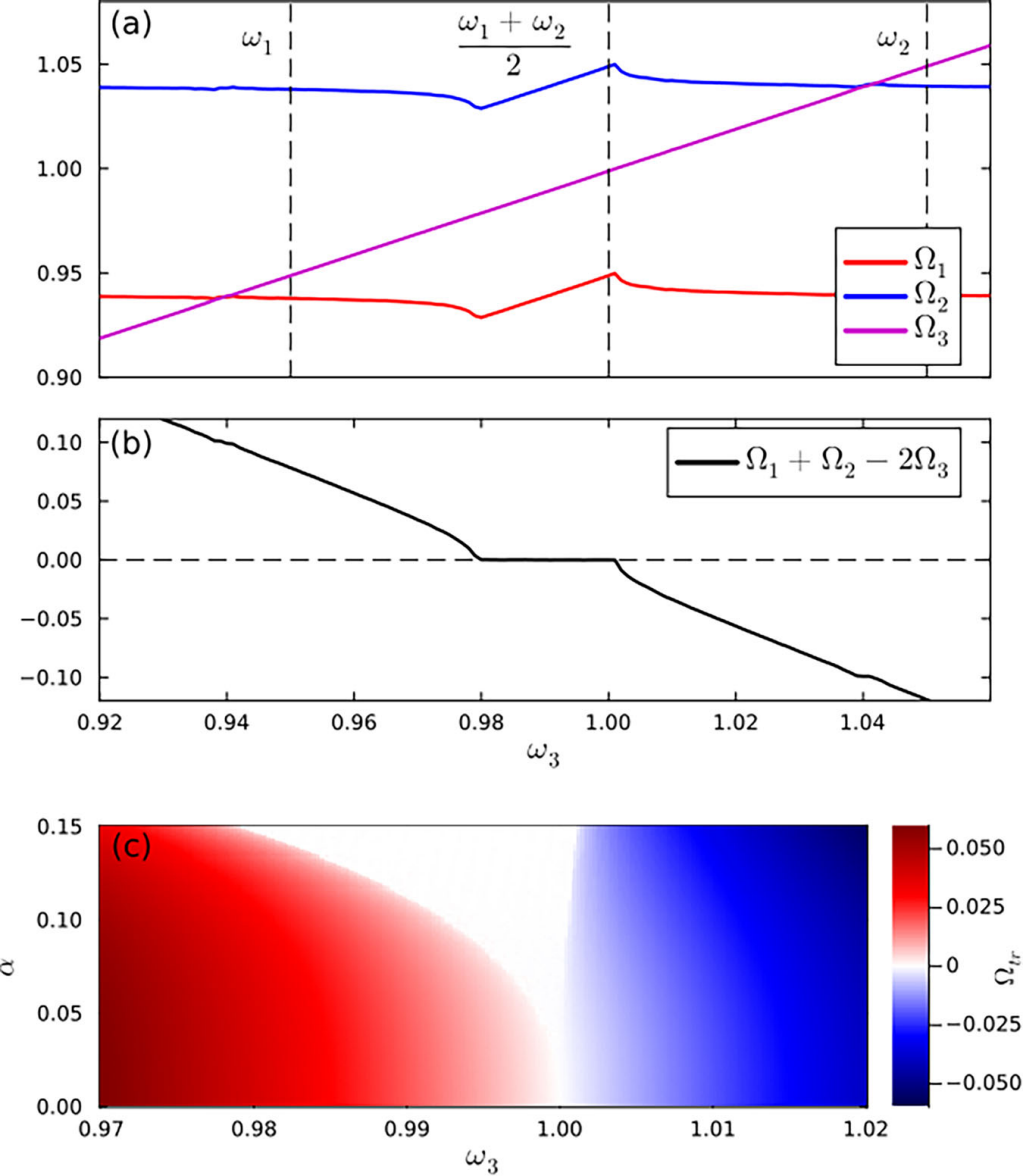
Hyperlocking between triplets of phases. **a** Shows effective frequencies for varying natural frequency *ω*_3_. **b** Shows the effective frequency triplet ${\Omega }_{{{\rm{tr}}}}={\Omega }_{1}+{\Omega }_{2}-2{\Omega }_{3}$ for varying *ω*_3_. The plateau at zero reveals the hyperlocking emergent from the triadic interaction. **c** Shows the quadratic locking cone that arises when also the coupling strength *α* is varied (white region where ${\Omega }_{{{\rm{tr}}}}=0$). All other parameters are the same as in Fig. 2.

To explore this hyperlocking further, we fix the parameters as before but vary the frequency *ω*_3_. For each value of *ω*_3_, we calculate numerically the effective frequencies *Ω*_1_, *Ω*_2_, and *Ω*_3_ shown in Fig. 3a. In panel (b), we show the triplet *Ω*_1_ + *Ω*_2_ − 2*Ω*_3_. In the vicinity of the triplet resonance of the natural frequencies at *ω*_3_ = (*ω*_1_ + *ω*_2_)/2, we observe a large plateau where the triadic coupling induces stable hyperlocking without any pairwise locking. The pairwise locking at the two points of pairwise resonance *ω*_3_ = *ω*_1,2_ is much less pronounced.

### A theory for emergent hyperlocking

To explain the hyperlocking behavior, we develop a normal form theory for the triadic network as shown in Supplementary Note II. We give a short outline of our treatment. For this triadic motif with the specific choice of the coupling functions (5), all coupling terms appearing in first order are non-resonant. This means that they represent small, rapid fluctuations that do not reveal the dynamics on larger time scales. To treat the higher-order terms, we employ techniques from normal form theory. The idea is to use successive coordinate transformations on the *z*-variables to remove as many terms as possible from Eq. (1), before performing the phase reduction. We show that non-resonant terms can be removed.

The coordinate transformations employed in our theoretical analysis are perturbations of the identity map. This proximity ensures that any locking relations observed in the transformed coordinates also manifest in the original system. Therefore, the analytical derivation based on transformed coordinates captures the actual dynamics of the original system.

After the normal form transformations, we obtain the phase equations to leading order

6 $$\dot{\phi }_{1}={\omega }_{1}^{{{\rm{eff}}}}-{\alpha }_{{{\rm{eff}}}}^{2}\sin (2{\phi }_{3}-{\phi }_{1}-{\phi }_{2}+{\gamma }_{1})$$

7 $$\dot{\phi }_{2}={\omega }_{2}^{{{\rm{eff}}}}-{\alpha }_{{{\rm{eff}}}}^{2}\sin (2{\phi }_{3}-{\phi }_{1}-{\phi }_{2}+{\gamma }_{1})$$

8 $$\dot{\phi }_{3}={\omega }_{3}$$

where ${\omega }_{i}^{{{\rm{eff}}}}$ and *α*_eff_ are the effective frequency and coupling functions. They are obtained in Supplementary Note II. We obtain that ${\omega }_{i}^{{{\rm{eff}}}}={\omega }_{i}+O({\alpha }^{2}\lambda )$, and ${\alpha }_{{{\rm{eff}}}}\approx \alpha \sqrt{\lambda /{\omega }_{3}}$. Notice that, to leading order, no pairwise synchronization is present. For instance, consider *θ* = *ϕ*_1_ − *ϕ*_2_ and a frequency gap *ω*_1_ − *ω*_2_ = *O*(*α*^2^). In this case, the higher-order corrections to Eqs. (6)–(8) are of order *O*(*α*^3^), which means that for small coupling, no pairwise locking will be present. Because to obtain a pairwise locking state, the frequency gap needs to be of the same order as the coupling strength. We provide the full argument in Supplementary Note III. On the other hand, we can obtain a stable triplet locking even in the absence of a pairwise locking. The hyperlocked state is hyperbolic and stable under small smooth perturbations.

Introducing the triplet phase

$$\varphi={\phi }_{1}+{\phi }_{2}-2{\phi }_{3},$$

we get the Adler equation

$$\dot{\varphi }=\Delta -2{\alpha }_{{{\rm{eff}}}}^{2}\sin ({\gamma }_{1}-\varphi )$$

where $\Delta={\omega }_{1}^{{{\rm{eff}}}}+{\omega }_{2}^{{{\rm{eff}}}}-2{\omega }_{3}$. In Supplementary Note III, we determine the frequency locking properties of such Adler equations. Hence, our analytical approach explains the numerically observed stable hyperlocking and, in particular, reproduces the plateau observed in Fig. 3b and the quadratic locking cone in Fig. 3c.

Note that the effective phase dynamics in Eqs. (6)–(8) contains only terms of order *O*(*α*^2^) and no Kuramoto-type pairwise sinusoidal coupling of the form $\sin ({\phi }_{j}-{\phi }_{k}+\gamma )$. This absence is a direct consequence of the underlying triadic interaction motif. This behavior is fundamentally different from that of the *classical Kuramoto model*, which arises as the phase reduction of Stuart-Landau oscillators with nearly identical natural frequencies and *pairwise linear coupling*. As shown in the Supplementary Note I, for purely pairwise linear coupling, the resonant pairwise terms persist under normal-form transformations and cannot be eliminated by nonlinear coordinate changes. Consequently, when pairs of oscillators are frequency-resonant, pairwise phase locking dominates the dynamics and is generically stable. In this situation, any apparent triplet locking then occurs only as a secondary consequence of pairwise locking.

### Synergy between triadic motif and nonlinear coupling

**Fig. 4:**
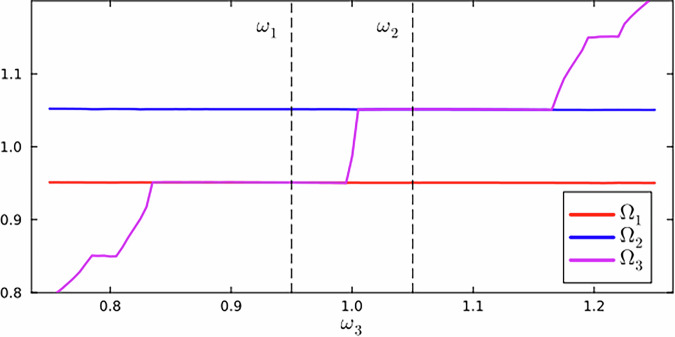
Different coupling functions may lead to pairwise locking. Using in system (1)–(5) the coupling function *h*(*z*, *w*) = *w*−*z*, pairwise locking becomes predominant. Around the corresponding resonances of the natural frequencies, there are two large regions with *Ω*_1_ = *Ω*_3_ and *Ω*_2_ = *Ω*_3_. Triplet locking 2*Ω*_1_ = *Ω*_2_ + *Ω*_3_ and 2*Ω*_3_ = *Ω*_1_ + *Ω*_2_ occurs only in two small regions close to the corresponding triplet resonances. Parameters as in Fig. 2.

The emergent dynamics of the triadic motif reflect a synergy between the hypernetwork structure and the nonlinearities of the coupling functions. For the class of coupling functions we consider, it is striking that, despite near full resonance, no pairwise synchronization occurs. A different choice of the coupling function, however, can restore stable pairwise locking: Fig. 4 shows that with the coupling function given as *h*(*z*, *w*) = *w* − *z*, the dynamics are dominated by pairwise interactions resulting in pairwise locking as can be seen by the plateaus where *Ω*_3_ = *Ω*_2_ and *Ω*_3_ = *Ω*_1_, which means that the hyperlocking effect disappears. Note that the coupling still has the same nonlinear triadic structure, only the specific monomials in the interaction are different. But this already results in new resonant terms that are essential for the effective phase dynamics.

### Hyperlocking with electrochemical oscillator network

**Fig. 5:**
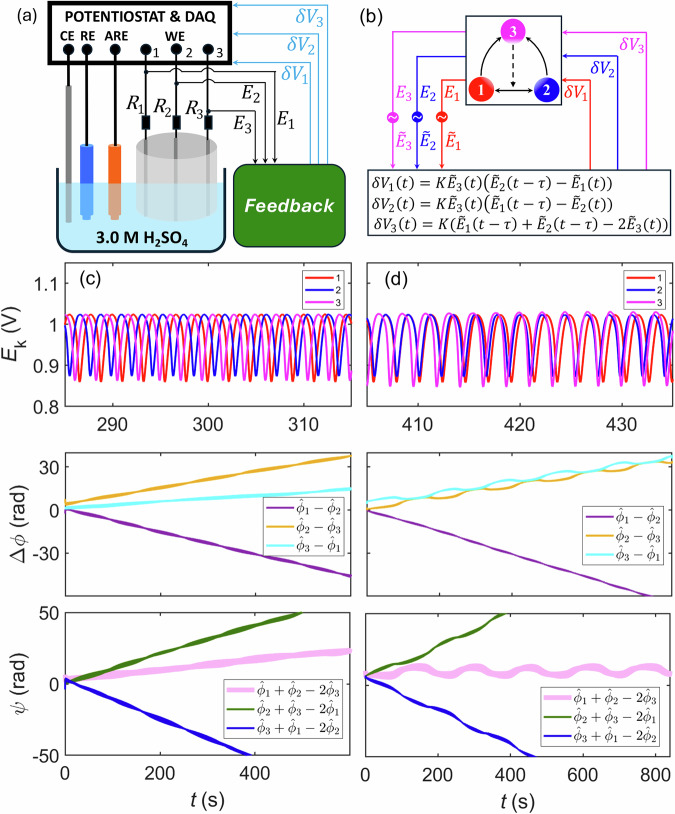
Hyperlocking in a triadic hypernetwork motif with electrochemical oscillators. **a** Experimental setup. CE, RE, ARE, and WE are the counter, reference, auxiliary reference, and the working electrodes. *R*_*k*_, *δV*_*k*_, and *E*_*k*_ denote the individual resistors, the circuit potential perturbations, and the measured electrode potentials, respectively. **b** Schematic illustration of the feedback mechanism. *K* and *τ* are the feedback strength and delay, and $\widetilde{{E}_{k}}$ are the rescaled electrode potentials used in the feedback scheme. **c**, **d** (Top to bottom) Electrode potential time series, evolution of pairwise and triplet phase differences with time, respectively. **c** Uncoupled oscillations. *K* = 0 V^−1^. **d** Hyperlocking in the presence of feedback. *K* = −0.2 V^−1^ and *τ* = 1.1 s.

We confirmed the emergence of triplet locking in the absence of pairwise phase locking through experiments involving three oscillatory electrochemical reactions coupled via nonlinear feedback and delay. The experimental setup, shown in Fig. 5, consists of a multichannel potentiostat connected to the three nickel working electrodes in sulfuric acid electrolyte. At a constant circuit potential with an added external resistance, the electrochemical dissolution of nickel exhibits periodic electrode potential (*E*_*k*_) oscillations reflecting the variations of the chemical composition of the metal-electrolyte interface.

We consider three such nickel wires that constitute the three nodes of the network. The network coupling is introduced using a real-time controller that implements an external feedback as

$$\delta {V}_{1}(t) 	=K{\widetilde{E}}_{3}(t)({\widetilde{E}}_{2}(t-\tau )-{\widetilde{E}}_{1}(t)) \\ \delta {V}_{2}(t) 	=K{\widetilde{E}}_{3}(t)({\widetilde{E}}_{1}(t-\tau )-{\widetilde{E}}_{2}(t)) \\ \delta {V}_{3}(t) 	=K(({\widetilde{E}}_{1}(t-\tau )-{\widetilde{E}}_{3}(t))+({\widetilde{E}}_{2}(t-\tau )-{\widetilde{E}}_{3}(t)))$$

where *δV*_*k*_ and ${\widetilde{E}}_{k}$(*t*) are the circuit potential perturbation applied via the feedback mechanism and the offset corrected and rescaled electrode potential of the *k*th electrode, respectively, *K* is the coupling strength, *τ* is a time delay. The circuit potentials of oscillators 1 and 2 depend nonlinearly on the states of all three oscillators. Further experimental details are given in Supplementary Note IV. The feedback scheme represents the hypernetwork: Nodes 1 and 2 are delay-coupled, and the coupling strength is modulated by node 3. Node 3 is coupled to nodes 1 and 2 through a delayed difference mechanism.

Note that without a delay term in the coupling, the system would settle into a pairwise, in-phase synchronized state, leading to regular phase locking^31^. In this sense, introducing delay in the experiments serves a role similar to introducing conjugate coupling in the model, namely, preventing stable pairwise synchronization. We adopted this form of coupling because delays are known to disrupt synchronization in coupled networks^32^. The delayed nonlinear feedback applied here does not produce obvious synchronization patterns, for example, one or multiple phase-cluster states^33^ due to the modulation of the coupling strength.

The triadic structure arises from the form of the coupling itself, while the delay enables access to a phase regime in which pairwise synchronization is destabilized and hyperlocking can emerge. In the weak-coupling regime, delays act by adjusting the effective phase of the interaction, playing a role akin to conjugate coupling^34^.

To describe the nature of the phase dynamics, the phases ${\widehat{\phi }}_{k}$, from the electrode potential oscillations, were extracted using the Hilbert transform technique^35^. The natural frequency of each oscillator was adjusted with a set of resistors so that *ω*_2_−*ω*_1_ = 12 mHz, *ω*_3_−*ω*_1_ = 3 mHz and *ω*_2_−*ω*_3_ = 9 mHz. The natural frequencies *ω*_1_  ≈ 448 mHz, *ω*_2_  ≈ 460 mHz, and *ω*_3_  ≈ 451 mHz create a condition for triplet resonance, as there is a small detuning for *ω*_1_ - 2*ω*_3_ + *ω*_2_ = 6 mHz. Figure 5c demonstrates that without coupling (*K* = 0 V^−1^), the electrode potentials of the three nodes oscillate independently, and the pairwise and triplet phase differences (including *φ*) vary linearly with time. In this state, there is no pairwise or triplet phase locking. Figure 5d shows the results in the presence of feedback with *K* = −0.2 V^−1^ and *τ* = 1.1 s (half the mean period of oscillations of the three nodes). No pairwise synchrony was observed: ${\widehat{\phi }}_{2}-{\widehat{\phi }}_{3}$ and ${\widehat{\phi }}_{3}-{\widehat{\phi }}_{1}$ show oscillations and drift with time. The triplet phase difference *φ* exhibited phase locking (hyperlocking, with 0 mHz triplet frequency difference) with a small amplitude oscillation, while the other two possible triplet phase differences drifted with time. The experiments demonstrate that hyperlocking is a robust phenomenon in a hypernetwork motif where pairwise locking is not possible due to the nature of the nonlinear interactions.

The electrochemical dynamics are chemically complex, involving multiple reaction pathways such as direct metal oxidation, the formation of oxide films, oxygen evolution, and the adsorption of chemical species within a diffuse double layer^36^. The experimental observation of hyperlocking, therefore, indicates that the phenomenon is robust to intrinsic nonlinearities, measurement noise, and slow experimental drifts. The results are also robust to the chosen coupling delay: Hyperlocking was also observed with delays set to 2–4% different than *T*/2 as shown in Supplementary Note IV. Although coupling was implemented using an external feedback loop, this setup effectively mimics realistic situations in which interaction strengths depend nonlinearly on the system state.

### Hyperlocking for other nonlinear oscillators and larger networks

So far, we have investigated hyperlocking in a triadic hypernetwork motif of three coupled oscillators near a Hopf bifurcation. In Supplementary Note V, we demonstrate numerically that a similar coupling structure can also induce hyperlocking in a system of three nonlinear van der Pol oscillators.

The hyperlocking mechanism uncovered here is not restricted to the specific triadic motif. Rather, this motif can serve as a fundamental building block for more complex networks that contain such triadic structures and exhibit hyperlocking behavior. In the Supplementary Note III, we demonstrate how our analytical theory can be applied similarly to an extended motif containing the triadic motif. Below, we give an example of a hypernetwork consisting of a set ${{\mathcal{N}}}=\{1,\ldots,n\}$ of oscillating nodes with randomly chosen directed pairwise links *j* → *k*, given by a directed graph ${{\mathcal{P}}}$, and an additional randomly selected collection ${{\mathcal{T}}}$ of triplet hyperlinks *k* → {*j*, *ℓ*}, where node *k* influences a pairwise interaction between nodes *j* and *ℓ*. For ${{\mathcal{T}}}$, we admit only triplets of nodes with no pairwise links in ${{\mathcal{P}}}$. The dynamics are given by

9 $$\dot{z}_{j}={f}_{j}({z}_{j})+\frac{\beta }{| {{\mathcal{P}}}| }{\sum }_{(j\to k)\in {{\mathcal{P}}}}q({z}_{k},{z}_{j}) \\+\frac{\alpha }{| {{\mathcal{T}}}| }{\sum }_{(k\to \{j,\ell \})\in {{\mathcal{T}}}}g({z}_{k})h({z}_{\ell },{z}_{j})$$

with Stuart-Landau oscillators *f*_*j*_ as in eq. (4) and triplet coupling functions *g* and *h* from (5) with coupling strength *α*. For the pairwise coupling, we choose *q*(*z*, *w*) = *z* − *w* with coupling strength *β*.

**Fig. 6:**
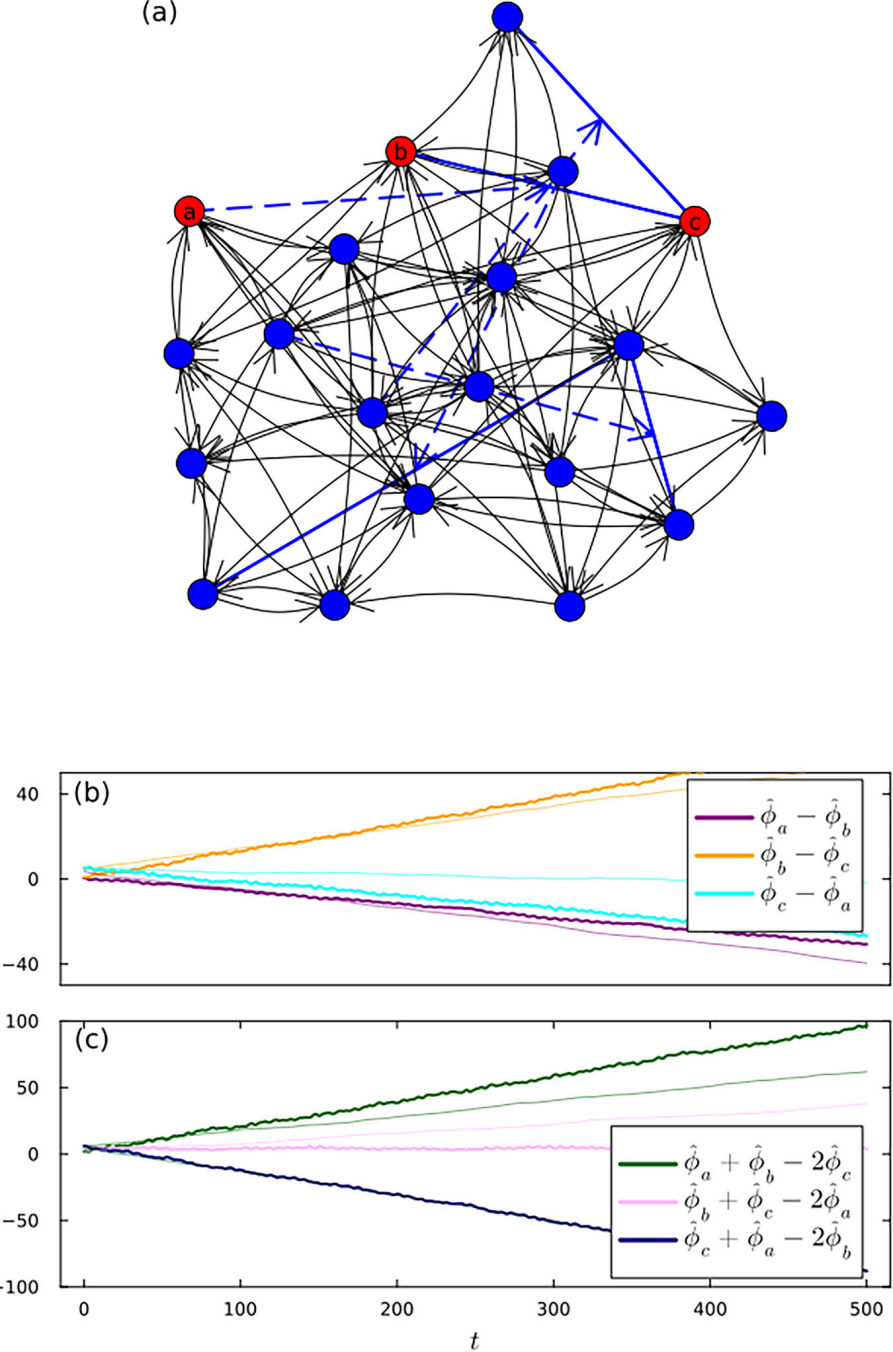
Hyperlocking in a larger network containing triadic motifs. **a** Random hypernetwork with directed pairwise links (black arrows) and triadic coupling (blue), where a third node influences (dashed blue arrow) the interaction between a pair (blue arrows). The triplet of red nodes has frequencies *ω*_*a*,*b*,*c*_ close to full resonance. All other natural frequencies are chosen randomly. The dynamics of system (9) shows a hyperlocking of this triplet for sufficiently strong triplet coupling strength *α* (dark colors), while for *α* = 0, i.e., only pairwise but no triplet coupling, no hyperlocking is present (light colors). **b** Pairwise frequency differences of the resonant triplet show no pairwise locking among these nodes. **c** Triplet phase differences show hyperlocking when triplet coupling is present. Parameters: *α* = 1.5,  *β* = 1.0,  *λ* = 0.3.

For the numerical simulations shown in Fig. 6, we have first generated a random hypernetwork with *n* = 20 nodes, 93 directed links, and four triplet hyperedges, see panel (a). After choosing random natural frequencies equally distributed in the interval [0.7, 1.3], we have slightly adapted the frequencies *ω*_*a*,*b*,*c*_ of the nodes in one of the hyperlinks to achieve a situation sufficiently close to a full resonance among these nodes. In panels (b) and (c), we plotted the pairwise and triplet phase combinations among these nodes with (dark colors) and without (light colors) the triplet coupling. The results clearly show that sufficiently strong triplet coupling induces hyperlocking among these nodes.

These cases illustrate that hyperlocking can emerge in extended systems through the embedding of triadic interactions, highlighting the broader relevance of this phenomenon for the dynamics of higher-order networks.

## Discussion

We have shown that hyperlocking emerges as a synergy between a triadic coupling structure and a nonlinear coupling function. We demonstrate that hyperlocking is a robust dynamical phenomenon that appears in a triadic motif of coupled Stuart-Landau oscillators, where it can be treated analytically.

The experiments on electrochemical oscillators demonstrated that hyperlocking is robust under realistic conditions, including experimental noise and slow parameter drifts caused by electrode deterioration. Although the oscillations originate from a Hopf bifurcation^37^, the observed waveforms indicate that the system operates far from the bifurcation point, as illustrated in Fig. 5, showing that hyperlocking is not a near-bifurcation effect. Consistently, we also observe hyperlocking in other dynamical settings, including networks of coupled van der Pol oscillators.

There is extensive literature showing that higher-order interactions can modify known dynamical phenomena in oscillator networks, such as chimeras^38^, synchronization thresholds^39^, and solitary states^40^, often by shifting parameter regimes or stability boundaries relative to purely pairwise coupling. Our result is qualitatively different. In the present system, when the coupling reduces to a purely pairwise network, no triplet locking appears. The hyperlocking reported here arises only from triadic interactions, rather than as a higher-order correction to effects of pairwise coupling. Our results also highlight that the boundary between network and hypernetwork dynamics is not clear-cut. Only the interplay between network structure, coupling functions, and resonances decides the true nature of the emergent dynamical interactions in the system.

Our analytical approach to this hyperlocking consists of performing a nonlinear change of coordinates that preserves dynamical phenomena. In these new coordinates, pairwise resonant terms are eliminated, while triadic interactions survive and organize the dynamics. Therefore, hyperlocking cannot be reduced to a sum of pairwise interactions. In certain scenarios, a higher-order interaction can be expressed as a combination of pairwise interactions, see for instance^41^. In this case, the same vector field can be represented in different bases. An instructive example is when the coupling function is linear, and the higher-order interaction can be expressed as a sum of pairwise interactions^42^. In this sense, higher-order or pairwise terms sometimes provide just alternative representations of the same system.

We introduce a nonlinear change of variables that maps the system to a dynamically equivalent vector field, retaining higher-order terms only when they are dynamically relevant. This coordinate transformation changes the vector field and allows us to demonstrate that hyperlocking is a dynamical effect induced by higher-order interactions, rather than a reparametrization of pairwise coupling. Triadic motifs emerge as flexible building blocks of collective behavior, capable of supporting synchronization regimes that cannot be sustained by networks with pairwise interactions alone, and point to a broader class of higher-order dynamical phenomena awaiting exploration.

Hyperlocking is a general synchronization mechanism in networks with higher-order interactions, in which certain expected frequency lockings, although formally possible, are not dynamically selected, and coherence emerges only through higher-order relations. Surprisingly, we show that hyperlocking is negligible in generic networks with purely pairwise coupling. Triadic networks, therefore, constitute minimal building blocks in which this phenomenon can occur. These findings demonstrate that, in networks with higher-order interactions, synchronization cannot in general be reduced to the analysis of pairwise correlations and is intrinsically multibody in nature. This opens several directions for future research, including extending the analysis to other bifurcation scenarios that generate periodic oscillations in the isolated system, examining whether similar effects arise in systems with symmetric multibody interactions, and exploring the possibility of hyperlocking between clusters of oscillators rather than between individual units.

## Supplementary information

Supplementary Information

Transparent Peer Review file

## Data Availability

Data from numerical simulations are provided at 10.5281/zenodo.19821152. Experimental source data for all figures are deposited at 10.5281/zenodo.19928267.

## Code availability

The numerical simulations and analysis routines used in this study were implemented in Julia, using the package ’DifferentialEquations.jl’^30^. The computational notebooks can be downloaded from 10.5281/zenodo.19821152.
