# Supplementary material for: Hypernetworks induce stable hyperlocking
Source: Nat Commun. 2026 Jun 24;17:7947. doi: 10.1038/s41467-026-74556-1 (PMC13448840; doi:10.1038/s41467-026-74556-1)
Supplement: Supplementary file 1 [file 41467_2026_74556_MOESM1_ESM.pdf]

Supplementary Information to the manuscript: "Hypernetworks induce stable hyperlocking"

Eddie Nijholt,[1] Tiago Pereira,[1,2] Matthias Wolfrum,[3] Sagnik Chakraborty,[4] István Z. Kiss,[4] and Jürgen Kurths[5]

[1]*Institute of Mathematical Sciences and Computation, University of São Paulo, São Paulo, Brazil*
[2]*Department of Mathematics, Imperial College London, London, UK*
[3]*Weierstrass Institute for Applied Analysis and Stochastics, Mohrenstr. 39, 10117 Berlin, Germany*
[4]*Department of Chemistry, Saint Louis University, St. Louis, MO 63025, USA.*
[5]*Potsdam Institute for Climate Impact Research - Telegraphenberg A 31, Potsdam, 14473, Germany*

SUPPLEMENTARY NOTE I: ABSENCE OF HYPERLOCKING IN GRAPHS

Hyperlocking, as seen in our triadic motif, is not a phenomenon associated with generic dyadic graphs. Here, we make this claim more precise, by showing that the terms associated with hyperlocking, e.g. $\sin / \cos(\phi_p - 2\phi_q + \phi_r)$, where p, q, r are distinct, do not show up in the phase reduced normal form up to leading order for dyadic networks with linear coupling.

The main idea is that each coupling term is either *resonant* or *non-resonant*. In our setting of full resonance, a term $z_1^{p_1} \dots z_n^{p_n} \overline{z}_1^{q_1} \dots \overline{z}_n^{q_n}$ is resonant when $p_1 + \dots + p_n - q_1 - \dots - q_n = 1$ and non-resonant otherwise. Resonant terms correspond to slow time-scale behavior of the system and may be used to predict quantitative phenomena such as synchronization and hyperlocking. Non-resonant terms, in contrast, correspond to rapid oscillations in the signal, and are often indistinguishable from noise in real-world systems. Another difference is that non-resonant terms can always be 'transformed away'. That is, for every non-resonant term, there exists a near-identity coordinate transformation that removes it from the equations. This introduces new terms, resonant and otherwise, though of a higher order in the coupling strength α.

An interesting situation happens when all coupling terms are non-resonant. In that case, the dynamics can only be elucidated from terms that appear after the coordinate transformation needed to remove these non-resonant terms. A priori, a linearly coupled dyadic network could present terms associated to hyperlocking in this way. We show that this is, nevertheless, not the case.

Thus, we consider the ODE:

$$\dot{z}_j = (\lambda + i\omega_j)z_j - z_j|z_j|^2 + \alpha \sum_{k=1}^{n} A_{j,k} z_k + \alpha \sum_{k=1}^{n} B_{j,k} \overline{z}_k \tag{1}$$

for $j \in \{1, \dots, n\}$. Here the $\omega_j \in \mathbb{R}$ are approximately equal, we assume $\alpha < \lambda \ll 1$ and the $A_{j,k}, B_{j,k} \in \mathbb{C}$ signify the (weighted) dyadic connection structure. To exclude pairwise synchronization at order α, we moreover assume the coupling is through non-resonant terms only. Therefore, we now assume

$$A_{j,k} = \begin{cases} a_j \in \mathbb{C} \text{ if } j = k; \\ 0 \text{ otherwise.} \end{cases} \tag{2}$$

Note that using the coupling function from the main text, $h(z, w) = w - \overline{z}$, gives a system as in Eq. (1), with Eq. (2) satisfied. It follows that we may write Eq. (1) as

$$\dot{z}_j = (\lambda + i\omega_j)z_j - z_j|z_j|^2 + \alpha a_j z_j + \alpha \sum_{k=1}^{n} B_{j,k} \overline{z}_k \tag{3}$$

To show our claim, we introduce the new coordinates:

$$u_j = z_j - \alpha \sum_{k=1}^{n} C_{j,k} \overline{z}_k \tag{4}$$

where

$$C_{j,k} := \frac{-B_{j,k}}{i(\omega_j + \omega_k)} \text{ for all } j, k \in \{1, \dots, n\}\,, \tag{5}$$

with the aim of removing the non-resonant coupling terms from Eq. (3). Note that, as u_j can be expressed as a near-identity $\mathbb{R}$-linear expression of $z_1, \ldots, z_n$ by Eq. (4), the same is true for z_j with respect to $u_1, \ldots, u_n$. More precisely, let us write C for the matrix of coefficients $C_{j,k}$, and similarly $z, u \in \mathbb{C}^n$ for the vectors with entries z_j and u_j, respectively. Eq. (4) then becomes

$$u = z - \alpha C\overline{z}\,. \tag{6}$$

It is not hard to verify that

$$z = u + \alpha C\overline{u} + \alpha^2 C\overline{C}u + \alpha^3 C\overline{C}C\overline{u} + \alpha^4 C\overline{C}C\overline{C}u + \ldots\,, \tag{7}$$

which converges for α sufficiently small. Combining Equations (3) and (4) gives us.

$$\begin{aligned}
\dot{u}_j &= \dot{z}_j - \alpha \sum_{k=1}^{n} C_{j,k}\dot{\overline{z}}_k \\
&= (\lambda + i\omega_j)z_j - z_j|z_j|^2 + \alpha a_j z_j + \alpha \sum_{k=1}^{n} B_{j,k}\overline{z}_k \\
&- \alpha \sum_{k=1}^{n} C_{j,k}\left((\lambda - i\omega_k)\overline{z}_k - \overline{z}_k|z_k|^2 + \alpha\overline{a}_k\overline{z}_k + \alpha\sum_{\ell=1}^{n}\overline{B}_{k,\ell}z_\ell\right) \\
&= (\lambda + i\omega_j)u_j - z_j|z_j|^2 \\
&+ \alpha\sum_{k=1}^{n}(\lambda + i\omega_j)C_{j,k}\overline{z}_k + \alpha\sum_{k=1}^{n}B_{j,k}\overline{z}_k - \alpha\sum_{k=1}^{n}(\lambda - i\omega_k)C_{j,k}\overline{z}_k \\
&+ \alpha a_j z_j + \alpha\sum_{k=1}^{n}C_{j,k}\overline{z}_k|z_k|^2 \\
&- \alpha^2\sum_{k=1}^{n}C_{j,k}\overline{a}_k\overline{z}_k - \alpha^2\sum_{k,\ell=1}^{n}C_{j,k}\overline{B}_{k,\ell}z_\ell \\
&= (\lambda + i\omega_j)u_j - z_j|z_j|^2 \\
&+ \alpha a_j z_j + \alpha\sum_{k=1}^{n}C_{j,k}\overline{z}_k|z_k|^2 \\
&- \alpha^2\sum_{k=1}^{n}C_{j,k}\overline{a}_k\overline{z}_k - \alpha^2\sum_{k,\ell=1}^{n}C_{j,k}\overline{B}_{k,\ell}z_\ell\,,
\end{aligned} \tag{8}$$

where in the last step we have used that

$$\begin{aligned}
&\alpha\sum_{k=1}^{n}(\lambda + i\omega_j)C_{j,k}\overline{z}_k + \alpha\sum_{k=1}^{n}B_{j,k}\overline{z}_k - \alpha\sum_{k=1}^{n}(\lambda - i\omega_k)C_{j,k}\overline{z}_k \\
=&\,\alpha\sum_{k=1}^{n}i(\omega_j + \omega_k)C_{j,k}\overline{z}_k + \alpha\sum_{k=1}^{n}B_{j,k}\overline{z}_k = 0\,.
\end{aligned} \tag{9}$$

We now focus on the term $z_j|z_j|^2$. By Equation (7), we may write

$$\begin{aligned}
z_j|z_j|^2 &= (u + \alpha C\overline{u} + \alpha^2 C\overline{C}u)_j^2(\overline{u} + \alpha\overline{C}u + \alpha^2\overline{C}C\overline{u})_j + \mathcal{O}(|\alpha|^3|u|^3) \\
&= u_j|u_j|^2 + \alpha(2(C\overline{u})_j|u_j|^2 + (\overline{C}u)_j u_j^2) \\
&+ \alpha^2(2|(C\overline{u})_j|^2 u_j + (C\overline{u})_j^2\overline{u}_j + 2|u_j|^2(C\overline{C}u)_j + u_j^2(\overline{C}C\overline{u})_j) + \mathcal{O}(|\alpha|^3|u|^3) \\
&= u_j|u_j|^2 + \alpha P_j(u) + \alpha^2(u_j Q_j(u) + R_j(u)) + \mathcal{O}(|\alpha|^3|u|^3)\,.
\end{aligned} \tag{10}$$

Here we have set

$$\begin{aligned}
P_j(u) &:= 2(C\overline{u})_j|u_j|^2 + (\overline{C}u)_j u_j^2 \\
Q_j(u) &:= 2|(C\overline{u})_j|^2 + 2\overline{u}_j(C\overline{C}u)_j + u_j(\overline{C}C\overline{u})_j \\
R_j(u) &:= (C\overline{u})_j^2\overline{u}_j\,.
\end{aligned} \tag{11}$$

Note that $P_j(u)$ and $R_j(u)$ consist of only non-resonant terms of degree 3. The polynomial $Q_j(u)$ consists of only terms of degree 2.

In a similar way, we find

$$\begin{aligned}\overline{z}_k|z_k|^2 &= (u+\alpha C\overline{u})_k(\overline{u}+\alpha\overline{C}u)_k^2 + \mathcal{O}(|\alpha|^2|u|^3) \\ &= \overline{u}_k|u_k|^2 + \alpha(2|u_k|^2(\overline{C}u)_k + (C\overline{u})_k\overline{u}_k^2) + \mathcal{O}(|\alpha|^2|u|^3) \\ &= \overline{u}_k|u_k|^2 + \alpha(|u_k|^2\hat{Q}_k(u) + \hat{P}_k(u)) + \mathcal{O}(|\alpha|^2|u|^3)\,.\end{aligned} \tag{12}$$

Here we have set $\hat{Q}_k(u) := 2(\overline{C}u)_k$ and $\hat{P}_k(u) := (C\overline{u})_k\overline{u}_k^2$, the latter of which is non-resonant. Putting these expressions for $z_j|z_j|^2$ and $\overline{z}_k|z_k|^2$ in Equation (8), we obtain

$$\begin{aligned}\dot{u}_j &= (\lambda+i\omega_j)u_j - z_j|z_j|^2 \\ &+ \alpha a_j z_j + \alpha\sum_{k=1}^n C_{j,k}\overline{z}_k|z_k|^2 \\ &- \alpha^2\sum_{k=1}^n C_{j,k}\overline{a}_k\overline{z}_k - \alpha^2\sum_{k,\ell=1}^n C_{j,k}\overline{B}_{k,\ell}z_\ell \\ &= (\lambda+i\omega_j)u_j - u_j|u_j|^2 \\ &+ \alpha a_j z_j + \alpha\sum_{k=1}^n C_{j,k}\overline{u}_k|u_k|^2 - \alpha P_j(u) \\ &- \alpha^2\sum_{k=1}^n C_{j,k}\overline{a}_k\overline{z}_k - \alpha^2\sum_{k,\ell=1}^n C_{j,k}\overline{B}_{k,\ell}z_\ell - \alpha^2(u_jQ_j(u)+R_j(u)) \\ &+ \alpha^2\sum_{k=1}^n C_{j,k}(|u_k|^2\hat{Q}_k(u) + \hat{P}_k(u)) + \mathcal{O}(|\alpha|^3|u|^3)\,.\end{aligned} \tag{13}$$

Next, concerning the linear coupling terms, we note that

$$\begin{aligned}&\alpha a_j z_j - \alpha^2\sum_{k=1}^n C_{j,k}\overline{a}_k\overline{z}_k - \alpha^2\sum_{k,\ell=1}^n C_{j,k}\overline{B}_{k,\ell}z_\ell \\ &= \alpha a_j u_j + \alpha^2\sum_{k=1}^n a_jC_{j,k}\overline{u}_k - \alpha^2\sum_{k=1}^n C_{j,k}\overline{a}_k\overline{u}_k - \alpha^2\sum_{k,\ell=1}^n C_{j,k}\overline{B}_{k,\ell}u_\ell + \mathcal{O}(|\alpha|^3|u|) \\ &= \alpha a_j u_j + \alpha^2\sum_{\ell=1}^n L_{j,\ell}\overline{u}_\ell - \alpha^2\sum_{\ell=1}^n M_{j,\ell}u_\ell + \mathcal{O}(|\alpha|^3|u|)\,.\end{aligned} \tag{14}$$

Here $L_{j,\ell} := a_jC_{j,\ell} - C_{j,\ell}\overline{a}_\ell$ and $M_{j,\ell} := \sum_{k=1}^n C_{j,k}\overline{B}_{k,\ell}$ may be seen as coupling weights capturing (part of) the new interaction structure.

Thus, we obtain

$$\begin{aligned}\dot{u}_j &= (\lambda+i\omega_j)u_j - u_j|u_j|^2 \\ &+ \alpha a_j u_j + \alpha\sum_{k=1}^n C_{j,k}\overline{u}_k|u_k|^2 - \alpha P_j(u) \\ &+ \alpha^2\sum_{\ell=1}^n L_{j,\ell}\overline{u}_\ell - \alpha^2\sum_{\ell=1}^n M_{j,\ell}u_\ell - \alpha^2(u_jQ_j(u)+R_j(u)) \\ &+ \alpha^2\sum_{k=1}^n C_{j,k}(|u_k|^2\hat{Q}_k(u) + \hat{P}_k(u)) + \mathcal{O}(|\alpha|^3|u|)\,.\end{aligned} \tag{15}$$

Now recall that

$$\sum_{k=1}^n C_{j,k}\overline{u}_k|u_k|^2 - P_j(u)$$

consists entirely of non-resonant terms of order 3. This means that a similar coordinate transformation as before can remove them. In turn, this yields new non-resonant terms of order 1 in α and 7 in u, as well as terms $\mathcal{O}(|\alpha^2||u|^5)$. Continuing this way, we may arrange, for any $N \in \mathbb{N}$, to have a near-identity coordinate transformation $v(u)$ in which the system is of the form

$$\begin{aligned}\dot{v}_j &= (\lambda + i\omega_j)v_j - v_j|v_j|^2 + \alpha a_j v_j \\ &+ \alpha^2 \sum_{\ell=1}^{n} L_{j,\ell}\overline{v}_\ell - \alpha^2 \sum_{\ell=1}^{n} M_{j,\ell} v_\ell - \alpha^2 (v_j Q_j(v) + R_j(v)) \\ &+ \alpha^2 \sum_{k=1}^{n} C_{j,k}(|v_k|^2 \hat{Q}_k(v) + \hat{P}_k(v)) + \mathcal{O}(|\alpha||v|^{2N+1} + |\alpha|^2|v|^5 + |\alpha|^3|v|)\,.\end{aligned} \tag{16}$$

More details about this latter procedure are provided in Supplementary Note II.

After this, we can similarly use coordinate transformations to remove non-resonant terms in order α^2. Specifically, these are the terms

$$\sum_{\ell=1}^{n} L_{j,\ell}\overline{v}_\ell\,, \;\; R_j(v) \;\text{ and }\; \sum_{k=1}^{n} C_{j,k}\hat{P}_k(v)\,. \tag{17}$$

Thus, after a final coordinate transformation $w(v)$, we obtain the system

$$\begin{aligned}\dot{w}_j &= (\lambda + i\omega_j)w_j - w_j|w_j|^2 + \alpha a_j w_j \\ &- \alpha^2 \sum_{\ell=1}^{n} M_{j,\ell} w_\ell - \alpha^2 w_j Q_j(w) + \alpha^2 \sum_{k=1}^{n} C_{j,k}|w_k|^2 \hat{Q}_k(w) \\ &+ \mathcal{O}(|\alpha||w|^{2N+1} + |\alpha|^2|w|^5 + |\alpha|^3|w|)\,.\end{aligned} \tag{18}$$

It is now straightforward to obtain the phase dynamics of this system to order α^2. Setting $w_j(t) = r_j(t)e^{i\phi_j(t)}$, we find

$$\dot{\phi}_j = \operatorname{Im}\left(\frac{\dot{w}_j}{w_j}\right)\,, \tag{19}$$

where $\operatorname{Im}(z)$ denotes the imaginary part of a complex number z. Note moreover that $r_j(t) = \sqrt{\lambda} + \mathcal{O}(|\alpha||\lambda|^{1/2})$.

The term $\alpha^2 \sum_{\ell=1}^{n} M_{j,\ell} w_\ell$ will only contribute terms $\alpha^2 c \sin(\phi_\ell - \phi_j + \gamma)$, for some constants $c, \gamma \in \mathbb{R}$, to the equations for $\dot{\phi}_j$.

Next, recall that we have set $Q_j(w) = 2|(C\overline{w})_j|^2 + 2\overline{w}_j(C\overline{C}w)_j + w_j(\overline{C}C\overline{w})_j$. It follows that

$$\begin{aligned}\operatorname{Im}\left(\frac{w_j Q_j(w)}{w_j}\right) &= \operatorname{Im}(Q_j(w)) = \operatorname{Im}(2|(C\overline{w})_j|^2 + 2\overline{w}_j(C\overline{C}w)_j + w_j(\overline{C}C\overline{w})_j) \\ &= \operatorname{Im}(2\overline{w}_j(C\overline{C}w)_j + w_j(\overline{C}C\overline{w})_j) = \operatorname{Im}(\overline{w}_j(C\overline{C}w)_j)\,.\end{aligned} \tag{20}$$

Thus the term $\alpha^2 w_j Q_j(w)$ contributes only terms $\alpha^2 \lambda c \sin(\phi_\ell - \phi_j + \gamma)$, for some constants $c, \gamma \in \mathbb{R}$, to the equations for $\dot{\phi}_j$.

Finally, as $\hat{Q}_k(w) = 2(\overline{C}w)_k$, we moreover find

$$\begin{aligned}\operatorname{Im}\left(\frac{\sum_{k=1}^{n} C_{j,k}|w_k|^2 \hat{Q}_k(w)}{w_j}\right) &= \sum_{k=1}^{n} \frac{|w_k|^2}{|w_j|^2} \operatorname{Im}\left(C_{j,k}\overline{w_j}\hat{Q}_k(w)\right) \\ &= \sum_{k=1}^{n} \operatorname{Im}\left(C_{j,k}\overline{w_j}\hat{Q}_k(w)\right) + \mathcal{O}(|\alpha|) \\ &= 2\sum_{k=1}^{n} \operatorname{Im}\left(C_{j,k}(\overline{C}w)_k\overline{w_j}\right) + \mathcal{O}(|\alpha|) \\ &= 2\operatorname{Im}\left(\overline{w_j}(C\overline{C}w)_j\right) + \mathcal{O}(|\alpha|)\,.\end{aligned} \tag{21}$$

Thus the term $\alpha^2 \sum_{k=1}^{n} C_{j,k}|w_k|^2\hat{Q}_k(w)$ likewise only contributes terms $\alpha^2\lambda C\sin(\phi_\ell - \phi_j + \gamma)$ to the equations for $\dot{\phi}_j$. This shows that up to leading order, the phase reduced equations for (1) have no triplet terms $\sin/\cos(\phi_p - 2\phi_q + \phi_r)$ for distinct p, q, r.

SUPPLEMENTARY NOTE II: TRIADIC NORMAL FORM REDUCTION

In this Supplementary Note we give an analytical treatment of the three-cell motif which is the main model of the paper. The techniques are very similar to those of Supplementary Note I. That is, we start with a coupled oscillator system where all coupling terms are non-resonant. We remove these terms using a number of subsequent coordinate transformations, after which we are left with leading order terms which are resonant. Basic first-order phase reduction then gives a dynamical equation for the evolution of the phases, from which the hyperlocking phenomena can be inferred.

Thus we consider the equations

$$\begin{aligned}\dot{z}_1 &= (\lambda + i\omega_1)z_1 - z_1|z_1|^2 + \alpha z_3(z_1 - \overline{z_2}) \\ \dot{z}_2 &= (\lambda + i\omega_2)z_2 - z_2|z_2|^2 + \alpha z_3(z_2 - \overline{z_1}) \\ \dot{z}_3 &= (\lambda + i\omega_3)z_3 - z_3|z_3|^2 + \alpha(z_3 - \overline{z_1}) + \alpha(z_3 - \overline{z_2})\,,\end{aligned} \tag{22}$$

corresponding to the dynamical system considered in the main text, where $h(z, w) = w - \overline{z}$ and $g(u) = u$. We moreover assume that $\alpha < \lambda \ll 1$. The main result we will prove in this section is as follows:

Theorem 1. *The phase-reduced equations of system* (22) *are conjugate to*

$$\begin{aligned}\dot{\theta}_1 &= \omega_1 + \alpha^2\frac{\lambda(\omega_3 - \omega_2 - \omega_1)}{\lambda^2 + (\omega_3 - \omega_2 - \omega_1)^2} - \alpha^2\frac{\lambda}{\sqrt{\lambda^2 + \omega_3^2}}\sin(\theta_1 + \theta_2 - 2\theta_3 + \gamma) \\ &\quad + \mathcal{O}(|\alpha||\lambda|^M + |\alpha|^2|\lambda|^2 + |\alpha|^3) \\ \dot{\theta}_2 &= \omega_2 + \alpha^2\frac{\lambda(\omega_3 - \omega_2 - \omega_1)}{\lambda^2 + (\omega_3 - \omega_2 - \omega_1)^2} - \alpha^2\frac{\lambda}{\sqrt{\lambda^2 + \omega_3^2}}\sin(\theta_1 + \theta_2 - 2\theta_3 + \gamma) \\ &\quad + \mathcal{O}(|\alpha||\lambda|^M + |\alpha|^2|\lambda|^2 + |\alpha|^3) \\ \dot{\theta}_3 &= \omega_3 + \mathcal{O}(|\alpha||\lambda|^M + |\alpha|^2|\lambda|^2 + |\alpha|^3)\,,\end{aligned} \tag{23}$$

where $M \in \mathbb{N}$ may be chosen arbitrarily large and where $\gamma \in \mathbb{R}$ is an argument of $\lambda + i\omega_3$.

Truncating the system of Equation (23), we see that a good approximation is given by

$$\begin{aligned}\dot{\theta}_1 &= \tilde{\omega}_1 - \alpha^2 C\sin(\theta_1 + \theta_2 - 2\theta_3 + \gamma) \\ \dot{\theta}_2 &= \tilde{\omega}_2 - \alpha^2 C\sin(\theta_1 + \theta_2 - 2\theta_3 + \gamma) \\ \dot{\theta}_3 &= \omega_3\,,\end{aligned} \tag{24}$$

where we have set

$$\tilde{\omega}_j := \omega_j + \alpha^2\frac{\lambda(\omega_3 - \omega_2 - \omega_1)}{\lambda^2 + (\omega_3 - \omega_2 - \omega_1)^2} \qquad \text{for } j \in \{1, 2\}\,, \tag{25}$$

with

$$C := \frac{\lambda}{\sqrt{\lambda^2 + \omega_3^2}} \tag{26}$$

and where γ is an argument of $\lambda + i\omega_3$.

We will arrive at Theorem 1 in two steps. More precisely, it follows directly from Theorems 2 and 3 below, the proofs of which will occupy the remainder of this section. In what follows, we write $z = (z_1, z_2, z_3) \in \mathbb{C}^3$ and likewise $q = (q_1, q_2, q_3) \in \mathbb{C}^3$.

Theorem 2. *For any $M' \in \mathbb{N}$, there exists a coordinate transformation $(z,\alpha) \mapsto (q,\alpha) = (\Phi(z,\alpha),\alpha)$ that transforms Equation (22) into*

$$\begin{aligned} \dot{q}_1 &= (\lambda + i\omega_1)q_1 - q_1|q_1|^2 + \alpha^2\left(\frac{q_3^2\overline{q}_2}{\lambda + i\omega_3} - \frac{|q_3|^2 q_1}{\lambda + i(\omega_3 - \omega_2 - \omega_1)}\right) \\ &\quad + \mathcal{O}(|\alpha||q|^{M'} + |\alpha|^2|q|^5 + |\alpha|^3|q|) \\ \dot{q}_2 &= (\lambda + i\omega_2)q_2 - q_2|q_2|^2 + \alpha^2\left(\frac{q_3^2\overline{q}_1}{\lambda + i\omega_3} - \frac{|q_3|^2 q_2}{\lambda + i(\omega_3 - \omega_2 - \omega_1)}\right) \\ &\quad + \mathcal{O}(|\alpha||q|^{M'} + |\alpha|^2|q|^5 + |\alpha|^3|q|) \\ \dot{q}_3 &= (\lambda + i\omega_3)q_3 - q_3|q_3|^2 + 2\alpha q_3 - 2\alpha^2 q_3|C_1\overline{q}_1 + C_2\overline{q}_2|^2 \\ &\quad + \mathcal{O}(|\alpha||q|^{M'} + |\alpha|^2|q|^5 + |\alpha|^3|q|)\,, \end{aligned} \tag{27}$$

for certain $C_1, C_2 \in \mathbb{C}$. This coordinate transformation is moreover near-identity, in the sense that

$$\Phi(z,\alpha) - z = \mathcal{O}(\|(\alpha,z)\|^2)\,. \tag{28}$$

Theorem 3. *The phase variables of Equation (27) are governed by Equation (23), where $M = \frac{M'-1}{2}$.*

In the next subsections, we prove Theorems 2 and 3 in that order.

Removing non-resonant terms for the first two oscillators: first reduction

We prove Theorem 2 by employing a number of consecutive near-identity coordinate transformations. For convenience, we now set

$$\begin{aligned} P_1(z) &= z_3(z_1 - \overline{z}_2)\,, &(29)\\ P_2(z) &= z_3(z_2 - \overline{z}_1) \text{ and} \\ P_3(z) &= (z_3 - \overline{z}_1) + (z_3 - \overline{z}_2) = 2z_3 - \overline{z}_1 - \overline{z}_2\,, &(30) \end{aligned}$$

so that Equation (22) may be summarized as

$$\dot{z}_k = (\lambda + i\omega_k)z_k - z_k|z_k|^2 + \alpha P_k(z) \quad \text{for } k \in \{1,2,3\}\,. \tag{31}$$

Note that the $P_k(z)$ are polynomials, consisting only of monomials of a single degree (degree 2 for $k = 1,2$ and degree 1 for $k = 3$).

We also introduce the augmented polynomials

$$\begin{aligned} Q_1(z) &= \frac{z_3 z_1}{\lambda + i\omega_3} - \frac{z_3\overline{z}_2}{\lambda + i(\omega_3 - \omega_2 - \omega_1)} \\ Q_2(z) &= \frac{z_3 z_2}{\lambda + i\omega_3} - \frac{z_3\overline{z}_1}{\lambda + i(\omega_3 - \omega_2 - \omega_1)}\,. \end{aligned} \tag{32}$$

These are selected to satisfy the relations

$$DQ_j(z)((\lambda + i\omega_k)z_k)_{k=1}^3 - (\lambda + i\omega_j)Q_j(z) = P_j(z) \tag{33}$$

for $j \in \{1,2\}$, as one can readily verify. We start with the near-identity coordinate transformation

$$\begin{aligned} u_j &= z_j - \alpha Q_j(z) \quad \text{for } j \in \{1,2\}\,, \\ u_3 &= z_3\,. \end{aligned} \tag{34}$$

Note that therefore

$$\begin{aligned} z_j &= u_j + \alpha Q_j(z) \quad \text{for } j \in \{1,2\}\,, \\ z_3 &= u_3\,. \end{aligned} \tag{35}$$

In these new coordinates, the equations of motion for u_3 are given by

$$\begin{aligned}\dot{u}_3 = \dot{z}_3 &= (\lambda + i\omega_3)z_3 - z_3|z_3|^2 + 2\alpha z_3 - \alpha(\overline{z}_1 + \overline{z}_2) \\ &= (\lambda + i\omega_3)u_3 - u_3|u_3|^2 + 2\alpha u_3 - \alpha(\overline{u}_1 + \overline{u}_2) - \alpha^2(\overline{Q_1(z)} + \overline{Q_2(z)}) \\ &= (\lambda + i\omega_3)u_3 - u_3|u_3|^2 + 2\alpha u_3 - \alpha(\overline{u}_1 + \overline{u}_2) \\ &- \alpha^2(\overline{Q_1(u)} + \overline{Q_2(u)}) + \mathcal{O}(|\alpha|^3|u|)\,.\end{aligned} \tag{36}$$

The equations of motion for u_j, $j \in \{1,2\}$, are given by

$$\begin{aligned}\dot{u}_j &= \dot{z}_j - \alpha DQ_j(z)(\dot{z}_k)_{k=1}^3 \\ &= (\lambda + i\omega_j)z_j - z_j|z_j|^2 + \alpha P_j(z) \\ &- \alpha DQ_j(z)((\lambda + i\omega_k)z_k - z_k|z_k|^2 + \alpha P_k(z))_{k=1}^3 \\ &= (\lambda + i\omega_j)z_j - z_j|z_j|^2 + \alpha P_j(z) \\ &- \alpha DQ_j(z)((\lambda + i\omega_k)z_k)_{k=1}^3 + \alpha DQ_j(z)(z_k|z_k|^2)_{k=1}^3 - \alpha^2 DQ_j(z)(P_k(z))_{k=1}^3 \\ &= (\lambda + i\omega_j)u_j + \alpha(\lambda + i\omega_j)Q_j(z) - z_j|z_j|^2 + \alpha P_j(z) \\ &- \alpha DQ_j(z)((\lambda + i\omega_k)z_k)_{k=1}^3 + \alpha DQ_j(z)(z_k|z_k|^2)_{k=1}^3 - \alpha^2 DQ_j(z)(P_k(z))_{k=1}^3\,.\end{aligned} \tag{37}$$

Rearranging terms, we obtain

$$\begin{aligned}\dot{u}_j &= (\lambda + i\omega_j)u_j - z_j|z_j|^2 + \alpha DQ_j(z)(z_k|z_k|^2)_{k=1}^3 \\ &+ \alpha\left((\lambda + i\omega_j)Q_j(z) + P_j(z) - DQ_j(z)((\lambda + i\omega_k)z_k)_{k=1}^3\right) \\ &- \alpha^2 DQ_j(z)(P_k(z))_{k=1}^3\,.\end{aligned} \tag{38}$$

By Equation (33), the second line in Equation (38) vanishes, and we are left with

$$\begin{aligned}\dot{u}_j &= (\lambda + i\omega_j)u_j - z_j|z_j|^2 + \alpha DQ_j(z)(z_k|z_k|^2)_{k=1}^3 \\ &- \alpha^2 DQ_j(z)(P_k(z))_{k=1}^3 \\ &= (\lambda + i\omega_j)u_j - z_j|z_j|^2 + \alpha DQ_j(z)(z_k|z_k|^2)_{k=1}^3 \\ &- \alpha^2 DQ_j(u)(P_k(u))_{k=1}^3 + \mathcal{O}(|\alpha|^3|u|)\,.\end{aligned} \tag{39}$$

We now focus our attention on the term

$$-z_j|z_j|^2 + \alpha DQ_j(z)(z_k|z_k|^2)_{k=1}^3 \tag{40}$$

appearing in Equation (39). From Equation (35) and the fact that Q_j is of degree 2, we see that

$$\begin{aligned}z_j|z_j|^2 &= (u_j + \alpha Q_j(z))(u_j + \alpha Q_j(z))(\overline{u}_j + \alpha\overline{Q_j(z)}) \\ &= u_j|u_j|^2 + \alpha(2|u_j|^2 Q_j(z) + u_j^2\overline{Q_j(z)}) + \mathcal{O}(|\alpha|^2|z|^4|u| + |\alpha|^3|z|^6) \\ &= u_j|u_j|^2 + \alpha(2|u_j|^2 Q_j(u) + u_j^2\overline{Q_j(u)}) + \mathcal{O}(|\alpha|^2|u|^5)\,.\end{aligned} \tag{41}$$

Note that the α-term $2|u_j|^2 Q_j(u) + u_j^2\overline{Q_j(u)}$ consists only of monomials of degree 4. More generally, Equation (41) still holds if $Q_j(z)$ consists of monomials of degree $\ell \geq 2$, in which case $2|u_j|^2 Q_j(u) + u_j^2\overline{Q_j(u)}$ consists only of monomials of degree $2 + \ell$. This plays a role later on, as our aim is to remove order α^1 terms from the initial Equation (22). More precisely, there will remain a term $\mathcal{O}(|\alpha||z|^{2N})$, but where N can be chosen arbitrarily high.

Returning to the term (40), Equation (41) gives us

$$\begin{aligned}&-z_j|z_j|^2 + \alpha DQ_j(z)(z_k|z_k|^2)_{k=1}^3 \\ =\ &-u_j|u_j|^2 - \alpha(2|u_j|^2 Q_j(u) + u_j^2\overline{Q_j(u)}) + \alpha DQ_j(u)(u_k|u_k|^2)_{k=1}^3 + \mathcal{O}(|\alpha|^2|u|^5) \\ =\ &-u_j|u_j|^2 + \alpha L_j(u) + \mathcal{O}(|\alpha|^2|u|^5)\,,\end{aligned} \tag{42}$$

where we have set

$$L_j(u) := -2|u_j|^2 Q_j(u) - u_j^2\overline{Q_j(u)}) + DQ_j(u)(u_k|u_k|^2)_{k=1}^3\,. \tag{43}$$

Note that L_j consists of monomials of degree 4, and more generally of degree $2+\ell$ if Q_j consists of monomials of degree $\ell \geq 2$.

We now apply Equation (42) to Equation (39), to obtain

$$\begin{aligned}\dot{u}_j &= (\lambda + i\omega_j)u_j - u_j|u_j|^2 + \alpha L_j(u) - \alpha^2 DQ_j(u)(P_k(u))_{k=1}^3 \\ &\quad + \mathcal{O}(|\alpha|^2|u|^5 + |\alpha|^3|u|)\,.\end{aligned} \tag{44}$$

Thus, combining Equation (44) with Equation (36) gives us

$$\begin{aligned}\dot{u}_1 &= (\lambda + i\omega_1)u_1 - u_1|u_1|^2 + \alpha L_1(u) - \alpha^2 DQ_1(u)(P_k(u))_{k=1}^3 \\ &\quad + \mathcal{O}(|\alpha|^2|u|^5 + |\alpha|^3|u|) \\ \dot{u}_2 &= (\lambda + i\omega_2)u_2 - u_2|u_2|^2 + \alpha L_2(u) - \alpha^2 DQ_2(u)(P_k(u))_{k=1}^3 \\ &\quad + \mathcal{O}(|\alpha|^2|u|^5 + |\alpha|^3|u|) \\ \dot{u}_3 &= (\lambda + i\omega_3)u_3 - u_3|u_3|^2 + 2\alpha u_3 - \alpha(\overline{u}_1 + \overline{u}_2) \\ &\quad - \alpha^2(\overline{Q_1(u)} + \overline{Q_2(u)}) + \mathcal{O}(|\alpha|^3|u|) \\ &= (\lambda + i\omega_3)u_3 - u_3|u_3|^2 + 2\alpha u_3 - \alpha(\overline{u}_1 + \overline{u}_2) + \alpha^2 M(u) + \mathcal{O}(|\alpha|^3|u|)\,.\end{aligned} \tag{45}$$

Here we have set

$$M(u) := -\overline{Q_1(u)} - \overline{Q_2(u)}\,. \tag{46}$$

which consists only of monomials of degree 2.

Removing non-resonant terms for the first two oscillators: cleanup

From Equation (45) we get the slightly weaker statement

$$\begin{aligned}\dot{u}_1 &= (\lambda + i\omega_1)u_1 - u_1|u_1|^2 + \alpha L_1(u) - \alpha^2 DQ_1(u)(P_k(u))_{k=1}^3 \\ &\quad + \mathcal{O}(|\alpha|^2|u|^4 + |\alpha|^3|u|) \\ \dot{u}_2 &= (\lambda + i\omega_2)u_2 - u_2|u_2|^2 + \alpha L_2(u) - \alpha^2 DQ_2(u)(P_k(u))_{k=1}^3 \\ &\quad + \mathcal{O}(|\alpha|^2|u|^4 + |\alpha|^3|u|) \\ \dot{u}_3 &= (\lambda + i\omega_3)u_3 - u_3|u_3|^2 + 2\alpha u_3 - \alpha(\overline{u}_1 + \overline{u}_2) + \alpha^2 M(u) + \mathcal{O}(|\alpha|^3|u|)\,.\end{aligned} \tag{47}$$

Note that we have only replaced $\mathcal{O}(|\alpha|^2|u|^5 + |\alpha|^3|u|)$ by $\mathcal{O}(|\alpha|^2|u|^4 + |\alpha|^3|u|)$ in the equations for $\dot{u}_1$ and $\dot{u}_2$. We will see that this ultimately makes no difference, as terms of order 4 in z are never resonant, but it makes the inductive argument below clearer.

We now assume that, in Equation (47) above, L_1 and L_2 are polynomials consisting only of monomials of some even degree $\ell \geq 4$, whereas M consists of monomials of degrees in $\{2, 4, \ldots, \ell - 2\}$. Because L_1 and L_2 consist of monomials of even degree, they cannot have resonant terms. Thus, there exist polynomials R_1 and R_2 consisting of monomials of degree ℓ that satisfy

$$DR_j(u)((\lambda + i\omega_k)u_k)_{k=1}^3 - (\lambda + i\omega_j)R_j(u) = L_j(u)\,, \tag{48}$$

for $j \in \{1, 2\}$. See *Supplementary Note 2* of [1] for a precise definition of resonant terms and a proof of the existence of corresponding polynomials R_1 and R_2. As before, we make a coordinate transformation

$$\begin{aligned}v_j &= u_j - \alpha R_j(u) \quad \text{for } j \in \{1, 2\}\,, \\ v_3 &= u_3\,.\end{aligned} \tag{49}$$

The equation for $\dot{v}_3$ becomes

$$
\begin{aligned}
\dot{v}_3 &= (\lambda + i\omega_3)v_3 - v_3|v_3|^2 + 2\alpha v_3 - \alpha(\overline{u}_1 + \overline{u}_2) + \alpha^2 M(u) + \mathcal{O}(|\alpha|^3|u|) \\
&= (\lambda + i\omega_3)v_3 - v_3|v_3|^2 + 2\alpha v_3 - \alpha(\overline{v}_1 + \overline{v}_2) - \alpha^2(\overline{R_1(u)} + \overline{R_2(u)}) \\
&+ \alpha^2 M(u) + \mathcal{O}(|\alpha|^3|u|) \\
&= (\lambda + i\omega_3)v_3 - v_3|v_3|^2 + 2\alpha v_3 - \alpha(\overline{v}_1 + \overline{v}_2) - \alpha^2(\overline{R_1(v)} + \overline{R_2(v)}) \\
&+ \alpha^2 M(v) + \mathcal{O}(|\alpha|^3|v|) \\
&= (\lambda + i\omega_3)v_3 - v_3|v_3|^2 + 2\alpha v_3 - \alpha(\overline{v}_1 + \overline{v}_2) + \alpha^2 \tilde{M}(v) + \mathcal{O}(|\alpha|^3|v|)\,.
\end{aligned} \tag{50}
$$

Here we have set

$$
\tilde{M}(v) = M(v) - \overline{R_1(v)} - \overline{R_2(v)}\,, \tag{51}
$$

which is a polynomial consisting of monomials of degrees in $\{2, 4, \dots, \ell\}$.

We now turn our attention to $\dot{v}_j$, for $j \in \{1, 2\}$. As before, we find

$$
\begin{aligned}
\dot{v}_j &= \dot{u}_j - \alpha DR_j(u)(\dot{u}_k)_{k=1}^3 \\
&= (\lambda + i\omega_j)u_j - u_j|u_j|^2 + \alpha L_j(u) - \alpha^2 DQ_j(u)(P_k(u))_{k=1}^3 \\
&- \alpha DR_j(u)((\lambda + i\omega_k)u_k - u_k|u_k|^2)_{k=1}^3 + \mathcal{O}(|\alpha|^2|u|^4 + |\alpha|^3|u|) \\
&= (\lambda + i\omega_j)v_j - u_j|u_j|^2 + \alpha DR_j(u)(u_k|u_k|^2)_{k=1}^3 \\
&+ \alpha(\lambda + i\omega_j)R_j(u) + \alpha L_j(u) - \alpha DR_j(u)((\lambda + i\omega_k)u_k)_{k=1}^3 \\
&- \alpha^2 DQ_j(u)(P_k(u))_{k=1}^3 + \mathcal{O}(|\alpha|^2|u|^4 + |\alpha|^3|u|) \\
&= (\lambda + i\omega_j)v_j - u_j|u_j|^2 + \alpha DR_j(u)(u_k|u_k|^2)_{k=1}^3 \\
&- \alpha^2 DQ_j(u)(P_k(u))_{k=1}^3 + \mathcal{O}(|\alpha|^2|u|^4 + |\alpha|^3|u|)\,,
\end{aligned} \tag{52}
$$

where in the last step we have used Equation (48). Just as in Equation (42), we find

$$
-u_j|u_j|^2 + \alpha DR_j(u)(u_k|u_k|^2)_{k=1}^3 = -v_j|v_j|^2 + \alpha\tilde{L}_j(v) + \mathcal{O}(|\alpha|^2|v|^4)\,, \tag{53}
$$

where $\tilde{L}_j(v)$ is a polynomial consisting of monomials of degree $\ell + 2$. Returning to Equation (52), we find

$$
\dot{v}_j = (\lambda + i\omega_j)v_j - v_j|v_j|^2 + \alpha\tilde{L}_j(v) - \alpha^2 DQ_j(v)(P_k(v))_{k=1}^3 + \mathcal{O}(|\alpha|^2|v|^4 + |\alpha|^3|v|)\,. \tag{54}
$$

Summarizing, we have now found

$$
\begin{aligned}
\dot{v}_1 &= (\lambda + i\omega_1)v_1 - v_1|v_1|^2 + \alpha\tilde{L}_1(v) - \alpha^2 DQ_1(v)(P_k(v))_{k=1}^3 + \mathcal{O}(|\alpha|^2|v|^4 + |\alpha|^3|v|) \\
\dot{v}_2 &= (\lambda + i\omega_2)v_2 - v_2|v_2|^2 + \alpha\tilde{L}_2(v) - \alpha^2 DQ_2(v)(P_k(v))_{k=1}^3 + \mathcal{O}(|\alpha|^2|v|^4 + |\alpha|^3|v|) \\
\dot{v}_3 &= (\lambda + i\omega_3)v_3 - v_3|v_3|^2 + 2\alpha v_3 - \alpha(\overline{v}_1 + \overline{v}_2) + \alpha^2\tilde{M}(v) + \mathcal{O}(|\alpha|^3|v|)\,.
\end{aligned} \tag{55}
$$

Here $\tilde{M}$ is a polynomial consisting of monomials of degrees in $\{2, 4, \dots, \ell\}$, and L_1 and L_2 are polynomials consisting only of monomials of degree $\ell + 2$. Thus, we may apply this argument successively, to arrive at the equations

$$
\begin{aligned}
\dot{w}_1 &= (\lambda + i\omega_1)w_1 - w_1|w_1|^2 - \alpha^2 DQ_1(w)(P_k(w))_{k=1}^3 \\
&+ \mathcal{O}(|\alpha||w|^{2N} + |\alpha|^2|w|^4 + |\alpha|^3|w|) \\
\dot{w}_2 &= (\lambda + i\omega_2)w_2 - w_2|w_2|^2 - \alpha^2 DQ_2(w)(P_k(w))_{k=1}^3 \\
&+ \mathcal{O}(|\alpha||w|^{2N} + |\alpha|^2|w|^4 + |\alpha|^3|w|) \\
\dot{w}_3 &= (\lambda + i\omega_3)w_3 - w_3|w_3|^2 + 2\alpha w_3 - \alpha(\overline{w}_1 + \overline{w}_2) + \alpha^2 K(w) + \mathcal{O}(|\alpha|^3|w|)\,.
\end{aligned} \tag{56}
$$

Here $N \in \mathbb{N}$ may be chosen arbitrarily high and K is a polynomial consisting of monomials of degrees in $\{2, 4, \dots, 2N - 2\}$.

Removing non-resonant terms for the third oscillator: first reduction

We now make the coordinate change

$$p_1 = w_1, \quad p_2 = w_2, \quad p_3 = w_3 + \frac{i\alpha}{\omega_1 + \omega_3}\overline{w}_1 + \frac{i\alpha}{\omega_2 + \omega_3}\overline{w}_2\,. \tag{57}$$

Note that this is a near-identity transformation, since we may view α as a variable as well. One verifies that the equations for $\dot{p}_1$ and $\dot{p}_2$ are the same as those for $\dot{w}_1$ and $\dot{w}_2$, respectively, but with the w-variables replaced by the p-variables in Equation (56). For brevity, we write

$$C_j = \frac{i}{\omega_j + \omega_3} \in \mathbb{C} \;\text{ for } j \in \{1, 2\}\,, \tag{58}$$

so that

$$p_3 = w_3 + \alpha C_1 \overline{w}_1 + \alpha C_2 \overline{w}_2\,. \tag{59}$$

Crucial about the numbers C_j, $j \in \{1, 2\}$, is that they satisfy

$$-(\lambda + i\omega_3)C_j + (\lambda - i\omega_j)C_j = 1\,, \tag{60}$$

as one easily verifies. For $\dot{p}_3$, we get

$$\begin{aligned}
\dot{p}_3 &= \dot{w}_3 + \alpha C_1 \dot{\overline{w}}_1 + \alpha C_2 \dot{\overline{w}}_2 \\
&= (\lambda + i\omega_3)w_3 - w_3|w_3|^2 + 2\alpha w_3 - \alpha(\overline{w}_1 + \overline{w}_2) + \alpha^2 K(w) \\
&+ \alpha C_1((\lambda - i\omega_1)\overline{w}_1 - \overline{w}_1|w_1|^2) + \alpha C_2((\lambda - i\omega_2)\overline{w}_2 - \overline{w}_2|w_2|^2) \\
&+ \mathcal{O}(|\alpha|^2|w|^{2N} + |\alpha|^3|w|) \\
&= (\lambda + i\omega_3)p_3 - w_3|w_3|^2 + 2\alpha w_3 \\
&- \alpha(\overline{w}_1 + \overline{w}_2) - \alpha(\lambda + i\omega_3)(C_1\overline{w}_1 + C_2\overline{w}_2) \\
&+ \alpha(C_1(\lambda - i\omega_1)\overline{w}_1 + C_2(\lambda - i\omega_2)\overline{w}_2) \\
&- \alpha C_1\overline{w}_1|w_1|^2 - \alpha C_2\overline{w}_2|w_2|^2 + \alpha^2 K(w) \\
&+ \mathcal{O}(|\alpha|^2|w|^{2N} + |\alpha|^3|w|)\,.
\end{aligned} \tag{61}$$

Gathering terms in $\alpha\overline{w}_1$ and $\alpha\overline{w}_2$, we may further write this as

$$\begin{aligned}
\dot{p}_3 &= (\lambda + i\omega_3)p_3 - w_3|w_3|^2 + 2\alpha w_3 \\
&+ \alpha\overline{w}_1(-1 - (\lambda + i\omega_3)C_1 + (\lambda - i\omega_1)C_1) \\
&+ \alpha\overline{w}_2(-1 - (\lambda + i\omega_3)C_2 + (\lambda - i\omega_2)C_2) \\
&- \alpha C_1\overline{w}_1|w_1|^2 - \alpha C_2\overline{w}_2|w_2|^2 + \alpha^2 K(w) \\
&+ \mathcal{O}(|\alpha|^2|w|^{2N} + |\alpha|^3|w|) \\
&= (\lambda + i\omega_3)p_3 - w_3|w_3|^2 + 2\alpha w_3 \\
&- \alpha C_1\overline{w}_1|w_1|^2 - \alpha C_2\overline{w}_2|w_2|^2 + \alpha^2 K(w) \\
&+ \mathcal{O}(|\alpha|^2|w|^{2N} + |\alpha|^3|w|)\,,
\end{aligned} \tag{62}$$

where in the last step we use Equation (60).

At this point, it is convenient to introduce some new notation. We write $\mathrm{NRT}(p)$ to denote a polynomial in p consisting only of non-resonant terms. In addition, we assume that $\mathrm{NRT}(0) = 0$, so that the polynomial in question has no constant term. Similar to the $\mathcal{O}$ notation, the exact polynomial that $\mathrm{NRT}(p)$ represents may differ per line. For instance, as K consists entirely of non-constant monomials of even degree (which are therefore all non-resonant)

we may replace $K(w)$ by a term $\mathrm{NRT}(w)$. This yields

$$\begin{aligned}\dot{p}_3 &= (\lambda + i\omega_3)p_3 - w_3|w_3|^2 + 2\alpha w_3 \\ &\quad - \alpha C_1 \overline{w}_1 |w_1|^2 - \alpha C_2 \overline{w}_2 |w_2|^2 + \alpha^2 K(w) \\ &\quad + \mathcal{O}(|\alpha|^2|w|^{2N} + |\alpha|^3|w|) \\ &= (\lambda + i\omega_3)p_3 - w_3|w_3|^2 + 2\alpha w_3 \\ &\quad - \alpha C_1 \overline{p}_1 |p_1|^2 - \alpha C_2 \overline{p}_2 |p_2|^2 + \alpha^2 K(p) \\ &\quad + \mathcal{O}(|\alpha|^2|p|^{2N} + |\alpha|^3|p|) \\ &= (\lambda + i\omega_3)p_3 - w_3|w_3|^2 + 2\alpha w_3 - \alpha C_1 \overline{p}_1 |p_1|^2 - \alpha C_2 \overline{p}_2 |p_2|^2 \\ &\quad + \alpha^2 \,\mathrm{NRT}(p) + \mathcal{O}(|\alpha|^2|p|^{2N} + |\alpha|^3|p|)\,.\end{aligned} \tag{63}$$

We now turn our attention to the terms $-w_3|w_3|^2$ and $2\alpha w_3$. First of all, we have

$$\begin{aligned}2\alpha w_3 &= 2\alpha p_3 - 2\alpha^2 C_1 \overline{w}_1 - 2\alpha^2 C_2 \overline{w}_2 \\ &= 2\alpha p_3 - 2\alpha^2 C_1 \overline{p}_1 - 2\alpha^2 C_2 \overline{p}_2 = 2\alpha p_3 + \alpha^2 \,\mathrm{NRT}(p)\,.\end{aligned} \tag{64}$$

Second, we find

$$\begin{aligned}w_3|w_3|^2 &= (p_3 - \alpha C_1 \overline{p}_1 - \alpha C_2 \overline{p}_2)^2 (\overline{p}_3 - \alpha \overline{C}_1 p_1 - \alpha \overline{C}_2 p_2) \\ &= p_3|p_3|^2 - \alpha(2C_1|p_3|^2\overline{p}_1 + 2C_2|p_3|^2\overline{p}_2 + \overline{C}_1 p_3^2 p_1 + \overline{C}_2 p_3^2 p_2) \\ &\quad + \alpha^2 \overline{p}_3 (C_1\overline{p}_1 + C_2\overline{p}_2)^2 + 2\alpha^2 p_3 |C_1\overline{p}_1 + C_2\overline{p}_2|^2 + \mathcal{O}(|\alpha|^3|p|^3) \\ &= p_3|p_3|^2 - \alpha(2C_1|p_3|^2\overline{p}_1 + 2C_2|p_3|^2\overline{p}_2 + \overline{C}_1 p_3^2 p_1 + \overline{C}_2 p_3^2 p_2) \\ &\quad + 2\alpha^2 p_3 |C_1\overline{p}_1 + C_2\overline{p}_2|^2 + \alpha^2 \,\mathrm{NRT}(p) + \mathcal{O}(|\alpha|^3|p|^3)\,.\end{aligned} \tag{65}$$

where in the last step we have used that $\alpha^2\overline{p}_3(C_1\overline{p}_1 + C_2\overline{p}_2)^2$ consists entirely of non-resonant terms. Note that the terms in α^1 are likewise all non-resonant, but we keep them written out explicitly for now. Combining equations (64) and (65) with Equation (63), we obtain

$$\begin{aligned}\dot{p}_3 &= (\lambda + i\omega_3)p_3 - w_3|w_3|^2 + 2\alpha w_3 - \alpha C_1 \overline{p}_1 |p_1|^2 - \alpha C_2 \overline{p}_2 |p_2|^2 \\ &\quad + \alpha^2 \,\mathrm{NRT}(p) + \mathcal{O}(|\alpha|^2|p|^{2N} + |\alpha|^3|p|) \\ &= (\lambda + i\omega_3)p_3 - p_3|p_3|^2 + 2\alpha p_3 \\ &\quad + \alpha(2C_1|p_3|^2\overline{p}_1 + 2C_2|p_3|^2\overline{p}_2 + \overline{C}_1 p_3^2 p_1 + \overline{C}_2 p_3^2 p_2) \\ &\quad - \alpha C_1 \overline{p}_1 |p_1|^2 - \alpha C_2 \overline{p}_2 |p_2|^2 \\ &\quad - 2\alpha^2 p_3 |C_1\overline{p}_1 + C_2\overline{p}_2|^2 \\ &\quad + \alpha^2 \,\mathrm{NRT}(p) + \mathcal{O}(|\alpha|^2|p|^{2N} + |\alpha|^3|p|) \\ &= (\lambda + i\omega_3)p_3 - p_3|p_3|^2 + 2\alpha p_3 + \alpha W(p) - 2\alpha^2 p_3 |C_1\overline{p}_1 + C_2\overline{p}_2|^2 \\ &\quad + \alpha^2 \,\mathrm{NRT}(p) + \mathcal{O}(|\alpha|^2|p|^{2N} + |\alpha|^3|p|)\,.\end{aligned} \tag{66}$$

where we have set

$$\begin{aligned}W(p) &= 2C_1|p_3|^2\overline{p}_1 + 2C_2|p_3|^2\overline{p}_2 + \overline{C}_1 p_3^2 p_1 + \overline{C}_2 p_3^2 p_2 \\ &\quad - C_1\overline{p}_1|p_1|^2 - C_2\overline{p}_2|p_2|^2\,.\end{aligned} \tag{67}$$

Note that $W(p)$ consists entirely of third order, non-resonant monomials. In summary, we now have

$$\begin{aligned}\dot{p}_1 &= (\lambda + i\omega_1)p_1 - p_1|p_1|^2 - \alpha^2 DQ_1(p)(P_k(p))_{k=1}^3 \\ &\quad + \mathcal{O}(|\alpha||p|^{2N} + |\alpha|^2|p|^4 + |\alpha|^3|p|) \\ \dot{p}_2 &= (\lambda + i\omega_2)p_2 - p_2|p_2|^2 - \alpha^2 DQ_2(p)(P_k(p))_{k=1}^3 \\ &\quad + \mathcal{O}(|\alpha||p|^{2N} + |\alpha|^2|p|^4 + |\alpha|^3|p|) \\ \dot{p}_3 &= (\lambda + i\omega_3)p_3 - p_3|p_3|^2 + 2\alpha p_3 + \alpha W(p) - 2\alpha^2 p_3 |C_1\overline{p}_1 + C_2\overline{p}_2|^2 \\ &\quad + \alpha^2 \,\mathrm{NRT}(p) + \mathcal{O}(|\alpha|^2|p|^{2N} + |\alpha|^3|p|)\,.\end{aligned} \tag{68}$$

Removing non-resonant terms for the third oscillator: cleanup

As before, we will now push the non-resonant terms in α^1 to arbitrary high order in p by successive coordinate transformations. To this end, we relax the $\mathcal{O}$-term in the last line of Equation (68), so that the equation for $\dot{p}_3$ reads

$$\begin{aligned}\dot{p}_3 &= (\lambda + i\omega_3)p_3 - p_3|p_3|^2 + 2\alpha p_3 + \alpha W(p) - 2\alpha^2 p_3|C_1\overline{p}_1 + C_2\overline{p}_2|^2 \\ &\quad + \alpha^2\,\mathrm{NRT}(p) + \mathcal{O}(|\alpha|^2|p|^4 + |\alpha|^3|p|)\,.\end{aligned} \tag{69}$$

We now assume that the term $W(p)$ in Equation (69) consists entirely of non-resonant monomials of degree $\ell \geq 3$. To remove them via coordinate transformation, we introduce the new variables

$$q_1 = p_1, \quad q_2 = p_2, \quad q_3 = p_3 - \alpha X(p)\,. \tag{70}$$

Here $X(p)$ is a polynomial consisting entirely of non-resonant monomials of degree $\ell \geq 3$, that satisfies

$$DX(p)((\lambda + i\omega_k)p_k)_{k=1}^3 - (\lambda + i\omega_3)X(p) = W(p)\,. \tag{71}$$

Again, the equations for the first two oscillators remain the same, up to the given accuracy of Equation (68). For the third oscillator, we obtain

$$\begin{aligned}\dot{q}_3 &= \dot{p}_3 - \alpha DX(p)(\dot{p}_k)_{k=1}^3 \\ &= (\lambda + i\omega_3)p_3 - p_3|p_3|^2 + 2\alpha p_3 + \alpha W(p) - 2\alpha^2 p_3|C_1\overline{p}_1 + C_2\overline{p}_2|^2 \\ &\quad - \alpha DX(p)((\lambda + i\omega_k)p_k - p_k|p_k|^2 + 2\alpha\delta_k^3 p_3)_{k=1}^3 \\ &\quad + \alpha^2\,\mathrm{NRT}(p) + \mathcal{O}(|\alpha|^2|p|^4 + |\alpha|^3|p|)\,.\end{aligned} \tag{72}$$

Here $\delta_k^3 \in \{0, 1\}$ is defined by

$$\delta_k^3 = \begin{cases} 1 & \text{if } k = 3\,; \\ 0 & \text{if } k \neq 3\,. \end{cases} \tag{73}$$

We first note that

$$\alpha DX(p)(2\alpha\delta_k^3 p_3)_{k=1}^3 = \alpha \sum_{k=1}^{3} \frac{\partial X(p)}{\partial p_k} 2\alpha\delta_k^3 p_3 = 2\alpha^2 \frac{\partial X(p)}{\partial p_3} p_3\,. \tag{74}$$

The monomials of this latter expression are all contained in $X(p)$ as well (though possibly with different coefficients). As these monomials are all non-resonant, we see that we may write $\alpha DX(p)(2\alpha\delta_k^3 p_3)_{k=1}^3 = \alpha^2\,\mathrm{NRT}(p)$. Likewise, we have

$$2\alpha p_3 = 2\alpha q_3 + 2\alpha^2 X(p) = 2\alpha q_3 + \alpha^2\,\mathrm{NRT}(p)\,. \tag{75}$$

Returning to Equation (72) and writing $p_3 = q_3 + \alpha X(p)$, we obtain

$$\begin{aligned}\dot{q}_3 &= (\lambda + i\omega_3)q_3 - p_3|p_3|^2 + 2\alpha q_3 - 2\alpha^2 p_3|C_1\overline{p}_1 + C_2\overline{p}_2|^2 \\ &\quad + \alpha DX(p)(p_k|p_k|^2)_{k=1}^3 \\ &\quad + \alpha(\lambda + i\omega_3)X(p) - \alpha DX(p)((\lambda + i\omega_k)p_k)_{k=1}^3 + \alpha W(p) \\ &\quad + \alpha^2\,\mathrm{NRT}(p) + \mathcal{O}(|\alpha|^2|p|^4 + |\alpha|^3|p|) \\ &= (\lambda + i\omega_3)q_3 - p_3|p_3|^2 + 2\alpha q_3 - 2\alpha^2 p_3|C_1\overline{p}_1 + C_2\overline{p}_2|^2 \\ &\quad + \alpha DX(p)(p_k|p_k|^2)_{k=1}^3 \\ &\quad + \alpha^2\,\mathrm{NRT}(p) + \mathcal{O}(|\alpha|^2|p|^4 + |\alpha|^3|p|)\,,\end{aligned} \tag{76}$$

where in the last step we have used Equation (71). Once again, we need to investigate the term $p_3|p_3|^2$. To this end, we see that

$$p_3|p_3|^2 = q_3|q_3|^2 + 2\alpha|q_3|^2 X(p) + \alpha q_3^2\overline{X(p)} + \mathcal{O}(|\alpha|^2|p|^4 + |\alpha|^3|p|)\,. \tag{77}$$

Thus, we arrive at

$$
\begin{aligned}
\dot{q}_3 &= (\lambda + i\omega_3)q_3 - q_3|q_3|^2 + 2\alpha q_3 - 2\alpha^2 p_3|C_1\overline{p}_1 + C_2\overline{p}_2|^2 \\
&\quad + \alpha DX(p)(p_k|p_k|^2)_{k=1}^3 - 2\alpha|q_3|^2 X(p) - \alpha q_3^2\overline{X(p)} \\
&\quad + \alpha^2\,\mathrm{NRT}(p) + \mathcal{O}(|\alpha|^2|p|^4 + |\alpha|^3|p|) \\
&= (\lambda + i\omega_3)q_3 - q_3|q_3|^2 + 2\alpha q_3 - 2\alpha^2 q_3|C_1\overline{q}_1 + C_2\overline{q}_2|^2 \\
&\quad + \alpha DX(q)(q_k|q_k|^2)_{k=1}^3 - 2\alpha|q_3|^2 X(q) - \alpha q_3^2\overline{X(q)} \\
&\quad + \alpha^2\,\mathrm{NRT}(q) + \mathcal{O}(|\alpha|^2|q|^4 + |\alpha|^3|q|) \\
&= (\lambda + i\omega_3)q_3 - q_3|q_3|^2 + 2\alpha q_3 - 2\alpha^2 q_3|C_1\overline{q}_1 + C_2\overline{q}_2|^2 + \alpha\tilde{W}(q) \\
&\quad + \alpha^2\,\mathrm{NRT}(q) + \mathcal{O}(|\alpha|^2|q|^4 + |\alpha|^3|q|)\,,
\end{aligned}
\tag{78}
$$

where we have set

$$
\tilde{W}(q) := DX(q)(q_k|q_k|^2)_{k=1}^3 - 2|q_3|^2 X(q) - q_3^2\overline{X(q)}\,. \tag{79}
$$

Note that $\tilde{W}(q)$ consists entirely of monomials of degree $\ell + 2$. Moreover, it is not hard to see that all these monomials are non-resonant, using the fact that those of the original $X(q)$ are non-resonant. This is proven formally in *Supplementary Note 2* of [1].

Using successive coordinate transformations, we may therefore arrive at

$$
\begin{aligned}
\dot{q}_1 &= (\lambda + i\omega_1)q_1 - q_1|q_1|^2 - \alpha^2 DQ_1(q)(P_k(q))_{k=1}^3 \\
&\quad + \mathcal{O}(|\alpha||q|^{2N} + |\alpha|^2|q|^4 + |\alpha|^3|q|) \\
\dot{q}_2 &= (\lambda + i\omega_2)q_2 - q_2|q_2|^2 - \alpha^2 DQ_2(q)(P_k(q))_{k=1}^3 \\
&\quad + \mathcal{O}(|\alpha||q|^{2N} + |\alpha|^2|q|^4 + |\alpha|^3|q|) \\
\dot{q}_3 &= (\lambda + i\omega_3)q_3 - q_3|q_3|^2 + 2\alpha q_3 - 2\alpha^2 q_3|C_1\overline{q}_1 + C_2\overline{q}_2|^2 + \alpha^2\,\mathrm{NRT}(q) \\
&\quad + \mathcal{O}(|\alpha||q|^{2N} + |\alpha|^2|q|^4 + |\alpha|^3|q|)\,.
\end{aligned}
\tag{80}
$$

where $N \in \mathbb{N}$ may be chosen arbitrarily high.

Removing higher order non-resonant terms

Motivated by Equation (80), we now study systems of the form

$$
\begin{aligned}
\dot{z}_1 &= (\lambda + i\omega_1)z_1 - z_1|z_1|^2 + \alpha^2 R_1(z) + \alpha^2 S_1(z) \\
&\quad + \mathcal{O}(|\alpha||z|^{2N} + |\alpha|^2|z|^5 + |\alpha|^3|z|) \\
\dot{z}_2 &= (\lambda + i\omega_2)z_2 - z_2|z_2|^2 + \alpha^2 R_2(z) + \alpha^2 S_2(z) \\
&\quad + \mathcal{O}(|\alpha||z|^{2N} + |\alpha|^2|z|^5 + |\alpha|^3|z|) \\
\dot{z}_3 &= (\lambda + i\omega_3)z_3 - z_3|z_3|^2 + 2\alpha z_3 + \alpha^2 R_3(z) + \alpha^2 S_3(z) \\
&\quad + \mathcal{O}(|\alpha||z|^{2N} + |\alpha|^2|z|^5 + |\alpha|^3|z|)\,,
\end{aligned}
\tag{81}
$$

where $R_j(z)$ and $S_j(z)$ are polynomials consisting of all resonant and all non-resonant monomials, respectively, for $j \in \{1, 2, 3\}$. Moreover $R_j(0) = S_j(0)$, so constant terms are absent. Note that, instead of a term $\mathcal{O}(|\alpha||z|^{2N} + |\alpha|^2|z|^4 + |\alpha|^3|z|)$ as in Equation (80), we write $\mathcal{O}(|\alpha||z|^{2N} + |\alpha|^2|z|^5 + |\alpha|^3|z|)$ in Equation (81). This is because monomials of even degree are always non-resonant, and so can be absorbed in the $S_j(z)$.

As the $S_j(z)$ are non-resonant, there exist polynomials $T_j(z)$, consisting of monomials of the same degree as those of $S_j(z)$, satisfying

$$
DT_j(z)((\lambda + i\omega_k)z_k)_{k=1}^3 - (\lambda + i\omega_j)T_j(z) = S_j(z)\,. \tag{82}
$$

We now make the coordinate change

$$
u_j = z_j - \alpha^2 T_j(z) \quad \text{for } j \in \{1, 2, 3\}\,. \tag{83}
$$

This gives us

$$\begin{aligned}
\dot{u}_j &= \dot{z}_j - \alpha^2 DT_j(z)(\dot{z}_k)_{k=1}^3 \\
&= (\lambda + i\omega_j)z_j - z_j|z_j|^2 + \delta_j^3 2\alpha z_j + \alpha^2 R_j(z) + \alpha^2 S_j(z) \\
&- \alpha^2 DT_j(z)((\lambda + i\omega_k)z_k - z_k|z_k|^2)_{k=1}^3 \\
&+ \mathcal{O}(|\alpha||z|^{2N} + |\alpha|^2|z|^5 + |\alpha|^3|z|) \\
&= (\lambda + i\omega_j)u_j + \alpha^2(\lambda + i\omega_j)T_j(z) - z_j|z_j|^2 + \delta_j^3 2\alpha u_j \\
&+ \alpha^2 R_j(u) + \alpha^2 S_j(z) - \alpha^2 DT_j(z)((\lambda + i\omega_k)z_k - z_k|z_k|^2)_{k=1}^3 \\
&+ \mathcal{O}(|\alpha||u|^{2N} + |\alpha|^2|u|^5 + |\alpha|^3|u|) \\
&= (\lambda + i\omega_j)u_j - z_j|z_j|^2 + \delta_j^3 2\alpha u_j + \alpha^2 R_j(u) + \alpha^2 DT_j(z)(z_k|z_k|^2)_{k=1}^3 \\
&+ \mathcal{O}(|\alpha||u|^{2N} + |\alpha|^2|u|^5 + |\alpha|^3|u|)\,,
\end{aligned} \tag{84}$$

where in the last step we have used Equation (82). It remains to investigate the terms $z_j|z_j|^2$ and $\alpha^2 DT_j(z)(z_k|z_k|^2)_{k=1}^3$. To this end, we have

$$\begin{aligned}
& -z_j|z_j|^2 + \alpha^2 DT_j(z)(z_k|z_k|^2)_{k=1}^3 \\
= \;& -u_j|u_j|^2 - 2\alpha^2|u_j|^2 T_j(z) - \alpha^2 u_j^2\overline{T_j(z)} + \alpha^2 DT_j(u)(u_k|u_k|^2)_{k=1}^3 \\
& + \mathcal{O}(|\alpha|^4|u|) \\
= \;& -u_j|u_j|^2 + \alpha^2 \tilde{S}_j(u) + \mathcal{O}(|\alpha|^4|u|)\,.
\end{aligned} \tag{85}$$

Here we have set

$$\tilde{S}_j(u) := -2|u_j|^2 T_j(u) - u_j^2\overline{T_j(u)} + DT_j(u)(u_k|u_k|^2)_{k=1}^3\,. \tag{86}$$

As in the previous subsection, $\tilde{S}_j(u)$ consists of all non-resonant terms because $T_j(u)$ does. Moreover, the lowest degree among the monomials in $\tilde{S}_j(u)$ is at least two more than that of $T_j(u)$, and so than that of $S_j(u)$. Returning to Equation (84), we obtain

$$\begin{aligned}
\dot{u}_j &= (\lambda + i\omega_j)u_j - u_j|u_j|^2 + \delta_j^3 2\alpha u_j + \alpha^2 R_j(u) + \alpha^2 \tilde{S}_j(u) \\
&+ \mathcal{O}(|\alpha||u|^{2N} + |\alpha|^2|u|^5 + |\alpha|^3|u|)\,.
\end{aligned} \tag{87}$$

Applying similar transformations successively, we may force the non-resonant terms $\alpha^2\tilde{S}_j(u)$ to be in $\mathcal{O}(|\alpha|^2|u|^5)$. Thus, we finally arrive at

$$\begin{aligned}
\dot{u}_j &= (\lambda + i\omega_j)u_j - u_j|u_j|^2 + \delta_j^3 2\alpha u_j + \alpha^2 R_j(u) \\
&+ \mathcal{O}(|\alpha||u|^{2N} + |\alpha|^2|u|^5 + |\alpha|^3|u|)\,.
\end{aligned} \tag{88}$$

We may therefore return to Equation (80) and select only the resonant terms from the given order α^2 terms. For $\dot{q}_3$, this is precisely $-2\alpha^2 q_3|C_1\overline{q}_1 + C_2\overline{q}_2|^2$, by definition of $\mathrm{NRT}(q)$. Thus we are left with the equations for $\dot{q}_1$ and $\dot{q}_2$, for which we have to split $DQ_1(q)(P_k(q))_{k=1}^3$ and $DQ_2(q)(P_k(q))_{k=1}^3$, respectively, into resonant and non-resonant terms. Using that even degree terms are always non-resonant, we get

$$\begin{aligned}
DQ_1(z)(P_k(z))_{k=1}^3 &= \frac{(2z_3 - \overline{z}_1 - \overline{z}_2)z_1}{\lambda + i\omega_3} - \frac{(2z_3 - \overline{z}_1 - \overline{z}_2)\overline{z}_2}{\lambda + i(\omega_3 - \omega_2 - \omega_1)} \\
&+ \frac{z_3(z_3(z_1 - \overline{z}_2))}{\lambda + i\omega_3} - \frac{z_3\overline{z_3(z_2 - \overline{z}_1)}}{\lambda + i(\omega_3 - \omega_2 - \omega_1)} \\
&= \frac{z_3^2 z_1 - z_3^2\overline{z}_2}{\lambda + i\omega_3} - \frac{|z_3|^2\overline{z}_2 - |z_3|^2 z_1}{\lambda + i(\omega_3 - \omega_2 - \omega_1)} + \mathrm{NRT}(z) \\
&= -\frac{z_3^2\overline{z}_2}{\lambda + i\omega_3} + \frac{|z_3|^2 z_1}{\lambda + i(\omega_3 - \omega_2 - \omega_1)} + \mathrm{NRT}(z)\,.
\end{aligned} \tag{89}$$

In exactly the same way, or realizing the $(z_1, z_2) \mapsto (z_2, z_1)$ symmetry between $DQ_1(z)(P_k(z))_{k=1}^3$ and $DQ_2(z)(P_k(z))_{k=1}^3$, we obtain

$$DQ_2(z)(P_k(z))_{k=1}^3 = -\frac{z_3^2 \overline{z}_1}{\lambda + i\omega_3} + \frac{|z_3|^2 z_2}{\lambda + i(\omega_3 - \omega_2 - \omega_1)} + \mathrm{NRT}(z)\,. \tag{90}$$

Thus, the final equations become

$$\begin{aligned}
\dot{q}_1 &= (\lambda + i\omega_1)q_1 - q_1|q_1|^2 + \alpha^2 \left(\frac{q_3^2 \overline{q}_2}{\lambda + i\omega_3} - \frac{|q_3|^2 q_1}{\lambda + i(\omega_3 - \omega_2 - \omega_1)} \right) \\
&\quad + \mathcal{O}(|\alpha||q|^{2N} + |\alpha|^2|q|^5 + |\alpha|^3|q|) \\
\dot{q}_2 &= (\lambda + i\omega_2)q_2 - q_2|q_2|^2 + \alpha^2 \left(\frac{q_3^2 \overline{q}_1}{\lambda + i\omega_3} - \frac{|q_3|^2 q_2}{\lambda + i(\omega_3 - \omega_2 - \omega_1)} \right) \\
&\quad + \mathcal{O}(|\alpha||q|^{2N} + |\alpha|^2|q|^5 + |\alpha|^3|q|) \\
\dot{q}_3 &= (\lambda + i\omega_3)q_3 - q_3|q_3|^2 + 2\alpha q_3 - 2\alpha^2 q_3 |C_1\overline{q}_1 + C_2\overline{q}_2|^2 \\
&\quad + \mathcal{O}(|\alpha||q|^{2N} + |\alpha|^2|q|^5 + |\alpha|^3|q|)\,.
\end{aligned} \tag{91}$$

We have now arrived at:

Proof of Theorem 2. Setting $M' = 2N$, we see that Equation (27) agrees with Equation (91). The latter was obtained from Equation (22) by applying a number of consecutive near-identity coordinate transformations. As the concatenation of multiple near-identity transformations is again near-identity, the result follows. □

Extracting the phase equations

It remains to prove Theorem 3. Writing $q_j(t) = r_j(t)e^{i\theta_j(t)}$, it is not hard to see that

$$\dot{\theta}_j = \mathrm{Im}\left(\frac{\dot{q}_j}{q_j}\right) = \mathrm{Im}\left(\frac{\dot{q}_j\overline{q_j}}{|q_j|^2}\right)\,, \tag{92}$$

where $\mathrm{Im}(z) \in \mathbb{R}$ denotes the imaginary part of a complex number z. We similarly note that

$$r_j(t) = \sqrt{\lambda} + \mathcal{O}(|\alpha||\lambda|^{1/2}). \tag{93}$$

Proof of Theorem 3. Applying Equation (92) to Equation (91) while using (93), we obtain

$$\begin{aligned}
\dot{\theta}_1 &= \omega_1 + \alpha^2\,\mathrm{Im}\left(\frac{q_3^2 \overline{q}_2 \overline{q}_1}{\lambda(\lambda + i\omega_3)} - \frac{|q_3|^2}{\lambda + i(\omega_3 - \omega_2 - \omega_1)} \right) \\
&\quad + \mathcal{O}(|\alpha||\lambda|^{N-1/2} + |\alpha|^2|\lambda|^2 + |\alpha|^3) \\
\dot{\theta}_2 &= \omega_2 + \alpha^2\,\mathrm{Im}\left(\frac{q_3^2 \overline{q}_2 \overline{q}_1}{\lambda(\lambda + i\omega_3)} - \frac{|q_3|^2}{\lambda + i(\omega_3 - \omega_2 - \omega_1)} \right) \\
&\quad + \mathcal{O}(|\alpha||\lambda|^{N-1/2} + |\alpha|^2|\lambda|^2 + |\alpha|^3) \\
\dot{\theta}_3 &= \omega_3 + \mathcal{O}(|\alpha||\lambda|^{N-1/2} + |\alpha|^2|\lambda|^2 + |\alpha|^3)\,.
\end{aligned} \tag{94}$$

It remains to write out the term

$$\alpha^2\,\mathrm{Im}\left(\frac{q_3^2 \overline{q}_2 \overline{q}_1}{\lambda(\lambda + i\omega_3)} - \frac{|q_3|^2}{\lambda + i(\omega_3 - \omega_2 - \omega_1)} \right)\,.$$

To this end, we write

$$\lambda + i\omega_3 = e^{i\gamma}\sqrt{\lambda^2 + \omega_3^2} \tag{95}$$

for some argument $\gamma \in [0, 2\pi)$, so that

$$\frac{1}{\lambda + i\omega_3} = \frac{e^{-i\gamma}}{\sqrt{\lambda^2 + \omega_3^2}}\,, \tag{96}$$

Note as well that

$$\frac{1}{\lambda + i(\omega_3 - \omega_2 - \omega_1)} = \frac{\lambda - i(\omega_3 - \omega_2 - \omega_1)}{\lambda^2 + (\omega_3 - \omega_2 - \omega_1)^2}\,. \tag{97}$$

We thus get

$$\begin{aligned}
&\alpha^2 \,\mathrm{Im}\left(\frac{q_3^2\overline{q}_2\overline{q}_1}{\lambda(\lambda + i\omega_3)} - \frac{|q_3|^2}{\lambda + i(\omega_3 - \omega_2 - \omega_1)}\right) \\
&= \alpha^2 \,\mathrm{Im}\left(\frac{\lambda^2 e^{i(2\theta_3 - \theta_1 - \theta_2 - \gamma)}}{\lambda\sqrt{\lambda^2 + \omega_3^2}} - \lambda\frac{\lambda - i(\omega_3 - \omega_2 - \omega_1)}{\lambda^2 + (\omega_3 - \omega_2 - \omega_1)^2}\right) + \mathcal{O}(|\alpha|^3) \\
&= \alpha^2 \frac{\lambda}{\sqrt{\lambda^2 + \omega_3^2}} \sin(2\theta_3 - \theta_1 - \theta_2 - \gamma) + \alpha^2 \frac{\lambda(\omega_3 - \omega_2 - \omega_1)}{\lambda^2 + (\omega_3 - \omega_2 - \omega_1)^2} + \mathcal{O}(|\alpha|^3)\,.
\end{aligned} \tag{98}$$

We arrive at

$$\begin{aligned}
\dot{\theta}_1 &= \omega_1 + \alpha^2 \frac{\lambda(\omega_3 - \omega_2 - \omega_1)}{\lambda^2 + (\omega_3 - \omega_2 - \omega_1)^2} - \alpha^2 \frac{\lambda}{\sqrt{\lambda^2 + \omega_3^2}} \sin(\theta_1 + \theta_2 - 2\theta_3 + \gamma) \\
&\quad + \mathcal{O}(|\alpha||\lambda|^{N-1/2} + |\alpha|^2|\lambda|^2 + |\alpha|^3) \\
\dot{\theta}_2 &= \omega_2 + \alpha^2 \frac{\lambda(\omega_3 - \omega_2 - \omega_1)}{\lambda^2 + (\omega_3 - \omega_2 - \omega_1)^2} - \alpha^2 \frac{\lambda}{\sqrt{\lambda^2 + \omega_3^2}} \sin(\theta_1 + \theta_2 - 2\theta_3 + \gamma) \\
&\quad + \mathcal{O}(|\alpha||\lambda|^{N-1/2} + |\alpha|^2|\lambda|^2 + |\alpha|^3) \\
\dot{\theta}_3 &= \omega_3 + \mathcal{O}(|\alpha||\lambda|^{N-1/2} + |\alpha|^2|\lambda|^2 + |\alpha|^3)\,.
\end{aligned} \tag{99}$$

The result follows by choosing $M = N - 1/2 = \frac{M'-1}{2}$, which may be chosen arbitrarily high. □

SUPPLEMENTARY NOTE III: EXTENDED MOTIF & HYPERLOCKING

In this Supplementary Note we show that hyperlocking is not limited to our three-cell motif, by providing a more elaborate example that we may nevertheless treat analytically. In addition, we obtain a first-order approximation for the boundary of the Arnold Tongue, which demonstrates its asymmetry.

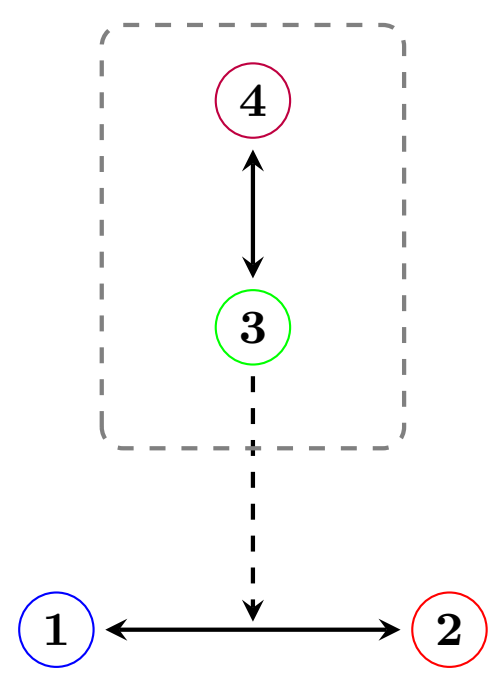

Supplementary Figure 1. **Extended triadic hypernetwork motif**. Pairwise interaction (black double arrow) between nodes 1 and 2 and a non-pairwise interaction between node 3 and nodes 1 and 2 since, node 3 modulates the coupling between nodes 1 and 2 (dashed arrow). Nodes 3 and 4 are bidirectionally coupled and grouped (dashed box).

The interaction structure we consider is depicted in Supplementary Figure 1 and corresponds to

$$\begin{aligned}
\dot{z}_1 &= f_1(z_1) + \alpha g(z_3) h(z_2, z_1) \\
\dot{z}_2 &= f_2(z_2) + \alpha g(z_3) h(z_1, z_2) \\
\dot{z}_3 &= f_3(z_3) + \beta q(z_4, z_3) \\
\dot{z}_4 &= f_4(z_4) + \beta q(z_3, z_4)
\end{aligned} \tag{100}$$

with z_j, $j \in \{1,2,3,4\}$ being the collections of dynamical variables for the four oscillators. For $\alpha = 0$, i.e., without coupling, each oscillator is assumed to have a periodic oscillation with frequency ω_j. As in the main text we choose:

$$\begin{aligned} h(z,w) &= w - \overline{z} \\ g(u) &= u\,. \end{aligned} \tag{101}$$

and additionally

$$q(z,w) = z - w.$$

We can obtain the triadic motif discussed in the paper by taking $f_4 = f_3$ for setting $\beta > 0$. Then, nodes 3 and 4 will lock and drive the connection between 1 and 2. For this motif, we introduce the two and four mismatch frequencies as

$$\Delta_2 = \frac{\omega_3 - \omega_4}{2} \quad \text{and} \quad \Delta_4 = \frac{\omega_1 + \omega_2 - \omega_3 - \omega_4}{2}$$

and obtain the following theorem:

Theorem 4 (Triplet Locking)**.** *Consider the model in Eq. (100). There exist $\lambda_0 > 0$ and $\alpha_{\max} > 0$ such that for all $0 < \lambda < \lambda_0$ and $0 < \alpha < \alpha_{\max}$, the following synchronization properties hold*

i) If $|\Delta_2| < \beta$ there is pairwise synchronization between nodes 3 and 4, that is,

$$\Omega_3 - \Omega_4 = 0$$

ii) There are constants $b_* = b_*(\lambda_0) > 0$ and $\rho = \rho(\lambda_0) > 0$ such that if

$$|\Delta_2| < \beta < b_* \lambda^3 \alpha^2 \text{ and } |\Delta_4| < \rho\lambda^4$$

then there are thresholds $0 < \alpha_1 < \alpha_2$ such that for all $\alpha \in (\alpha_1, \alpha_2)$

$$\Omega_1 + \Omega_2 - 2\Omega_3 = 0$$

that is, there is triplet synchronization

iii) There is a constant K such that if

$$|\omega_1 - \omega_2| > K\alpha_{\max}^3 \text{ and } |\omega_1 - \omega_3| > K\alpha_{\max}^2$$

then there is no pairwise synchronization between nodes $1, 2$ and 3.

Auxiliary Results

Lemma 1. *Let $\omega > 0$ and $0 < \lambda < \lambda_0 \ll \omega$. Let F, G, H be functions that map 0 to 0 with Lipschitz constant less than K in a neighborhood of zero. For $\beta = b\alpha^2$ with $b > 0$, define*

$$A_\lambda(\beta) = \frac{\lambda}{\sqrt{\lambda^2 + (\omega + G(\beta))^2}}, \qquad B_\lambda(\beta) = \frac{\lambda(\omega + \Delta + F(\beta))}{\lambda^2 + (\omega + \Delta + F(\beta))^2},$$

and set

$$m(\alpha) = \alpha^2 A_\lambda(\beta), \qquad c(\alpha) = \Delta + H(\beta) + \alpha^2 B_\lambda(\beta).$$

Then, for λ_0 small enough such that $0 < \lambda \leq \lambda_0 \ll \omega$ and for $\alpha^2 < \lambda$, one has

$$m(\alpha) - |c(\alpha)| \;\geq\; \alpha^2 \left(M_0 - Kb\right) \;-\; 2|\Delta| \;-\; C\alpha^4,$$

where $M_0 := \mu\lambda^3/\omega^3$ with $\mu = \mu(\lambda_0) > 0$ and $C := 2Kb/\lambda$.

Proof. By the triangle inequality and $|H(\beta)| \le K\beta$,

$$m(\alpha) - |c(\alpha)| \ge \alpha^2 A_\lambda(\beta) - |\Delta| - K\beta - \alpha^2 |B_\lambda(\beta)|.$$

Define $A_0 = \lambda/\sqrt{\lambda^2+\omega^2}$ and $B_0 = \lambda\omega/(\lambda^2+\omega^2)$, and write $A_\lambda(\beta) = A_0 + [A_\lambda(\beta) - A_0]$ and $|B_\lambda(\beta)| \le |B_0| + |B_\lambda(\beta) - B_0|$ to get

$$m(\alpha) - |c(\alpha)| \ge \alpha^2(A_0 - |B_0|) - |\Delta| - Kb\,\alpha^2 - \alpha^2(|A_\lambda(\beta) - A_0| + |B_\lambda(\beta) - B_0|)\,.$$

By the mean–value theorem,

$$\left|\partial_x \frac{\lambda}{\sqrt{\lambda^2+x^2}}\right| = \frac{\lambda|x|}{(\lambda^2+x^2)^{3/2}} \le \frac{2}{3\sqrt{3}}\cdot\frac{1}{\lambda}, \qquad \left|\partial_y \frac{\lambda y}{\lambda^2+y^2}\right| = \frac{\lambda|\lambda^2-y^2|}{(\lambda^2+y^2)^2} \le \frac{1}{\lambda}.$$

Hence,

$$|A_\lambda(\beta) - A_0| \le \frac{2}{3\sqrt{3}}\frac{1}{\lambda}|G(\beta)| \ \text{ and } \ |B_\lambda(\beta) - B_0| \le \frac{1}{\lambda}(|\Delta| + |F(\beta)|).$$

Using $|F(\beta)|, |G(\beta)| \le K\beta = Kb\,\alpha^2$ yields

$$\alpha^2(\,|A_\lambda(\beta) - A_0| + |B_\lambda(\beta) - B_0|\,) \ \le \ \frac{\alpha^2}{\lambda}\,|\Delta| \ + \ \frac{Kb}{\lambda}\left(1 + \frac{2}{3\sqrt{3}}\right)\alpha^4 \ < \ \frac{\alpha^2}{\lambda}\,|\Delta| \ + \ \frac{2Kb}{\lambda}\,\alpha^4.$$

Using that $\alpha^2 < \lambda$, and defining $M := A_0 - |B_0|$ and $C := \frac{2Kb}{\lambda}$, we obtain

$$m(\alpha) - |c(\alpha)| > \alpha^2(M - Kb) - 2|\Delta| - C\alpha^4$$

Next, notice that

$$M = \frac{\lambda}{\sqrt{\lambda^2+\omega^2}} - \frac{\lambda\omega}{\lambda^2+\omega^2} = \frac{x^3}{(1+x^2)\big(\sqrt{1+x^2}+1\big)} \ \text{ with } x = \frac{\lambda}{\omega},$$

so that

$$M \ \ge \ M_0 := \mu\,\frac{\lambda^3}{\omega^3}, \qquad \mu := \frac{1}{(1+x_0^2)\big(\sqrt{1+x_0^2}+1\big)} \approx \frac{1}{2},$$

where $x_0 = \lambda_0/\omega$. This gives the desired bound via the monotonicity of the denominator on $[0, x_0]$. □

Proposition 1 (Existence of fixed points)**.** *Fix $\omega > 0$ and $0 < \lambda \ll \omega$. Let F, G, H be functions that map 0 to 0 with Lipschitz constant less than K in a neighborhood of zero. For $\beta = b\alpha^2$ with $b > 0$, consider*

$$\dot\phi = c(\alpha) - m(\alpha)\sin(\phi+\gamma), \qquad m(\alpha) = \alpha^2 A_\lambda(\beta), \quad c(\alpha) = \Delta + H(\beta) + \alpha^2 B_\lambda(\beta),$$

with

$$A_\lambda(\beta) = \frac{\lambda}{\sqrt{\lambda^2 + (\omega + G(\beta))^2}}, \qquad B_\lambda(\beta) = \frac{\lambda(\omega + \Delta + F(\beta))}{\lambda^2 + (\omega+\Delta+F(\beta))^2}.$$

For all $\alpha^2 < \lambda$, there is $\mu = \mu(\lambda_0) > 0$ such that if

$$0 < b < b_0 := \frac{\mu\lambda^3}{2K\omega^3} \qquad \textit{and} \qquad |\Delta| < \delta_0 := \frac{\mu}{32}\frac{\lambda^4}{\omega^3},$$

then there exist thresholds $0 < \alpha_c^1 < \alpha_c^2$ such that for every $\alpha \in (\alpha_c^1, \alpha_c^2)$ the equation has exactly two equilibria (mod 2π) one stable and one unstable.

Proof. Lower threshold. Introduce

$$m(\alpha) := \ \alpha^2 A_\lambda(\beta) \text{ and } c(\alpha) := \Delta + H(\beta) + \alpha^2 B_\lambda(\beta)$$

and write

$$\dot{\phi} = f(\phi) = c(\alpha) - m(\alpha)\sin(\phi + \gamma).$$

From Lemma 1,

$$m(\alpha) - |c(\alpha)| > \alpha^2\left(\mu\frac{\lambda^3}{\omega^3} - Kb\right) - 2|\Delta| - \frac{2Kb}{\lambda}\alpha^4 > 0.$$

Setting $t = \alpha^2$, the inequality becomes

$$-\frac{2Kb}{\lambda}t^2 + \left(\mu\frac{\lambda^3}{\omega^3} - Kb\right)t - 2|\Delta| > 0.$$

Thus, we require

$$\mu\frac{\lambda^3}{\omega^3} - Kb > 0 \quad \text{and} \quad \left(\mu\frac{\lambda^3}{\omega^3} - Kb\right)^2 - 16\frac{Kb}{\lambda}|\Delta| > 0.$$

Lets define

$$b_0 := \mu\frac{\lambda^3}{2K\omega^3} \text{ and } \delta_0 := \frac{\mu}{32}\frac{\lambda^4}{\omega^3},$$

then for all $b < b_0$ and $|\Delta| < \delta_0$ we have two positive roots. When this holds, the roots of the quadratic equation satisfy $0 < t_- < t_+$ and we set $\alpha_c^1 = \sqrt{t_-}$ and $\alpha_c^2 = \sqrt{t_+}$.

Step 2 (equilibria and their type). For each such α, the equation $f(\phi) = 0$, since $|c| < m$, has two solutions on $[0, 2\pi)$:

$$\sin(\phi_s + \gamma) = \frac{c(\alpha)}{m(\alpha)}, \qquad \sin(\phi_u + \gamma) = \frac{c(\alpha)}{m(\alpha)}, \qquad \cos(\phi_s + \gamma) > 0, \ \ \cos(\phi_u + \gamma) < 0.$$

In fact, on any compact $[\alpha_-, \alpha_+] \subset (\alpha_c^1, \alpha_c^2)$,

$$\partial_\phi f\big(\phi_s(\alpha); \alpha\big) = -\sqrt{m(\alpha)^2 - c(\alpha)^2} < -\rho < 0,$$

which is the claimed uniform hyperbolicity bound. □

Proof of the Theorem 4 on Triplet locking

Proof. From our normal form theory there exist $\lambda_0 > 0$ and $\alpha_{\max} > 0$, such that for all $0 < \lambda < \lambda_0$ and $0 < \alpha < \alpha_{\max}$ there is a coordinate transformation that brings the phase equations to the following form

$$\begin{aligned}
\dot{\phi}_1 &= \hat{\omega}_1 - \alpha^2\frac{\lambda}{\sqrt{\lambda^2 + \omega_3^2}}\sin(\phi_1 + \phi_2 - 2\phi_3 + \gamma) + \alpha^3 R_1(\phi_1, \phi_2, \phi_3) + \lambda^2\alpha^2\hat{R}_1(\phi_1, \phi_2, \phi_3)\\
\dot{\phi}_2 &= \hat{\omega}_2 - \alpha^2\frac{\lambda}{\sqrt{\lambda^2 + \omega_3^2}}\sin(\phi_1 + \phi_2 - 2\phi_3 + \gamma) + \alpha^3 R_2(\phi_1, \phi_2, \phi_3) + \lambda^2\alpha^2\hat{R}_2(\phi_1, \phi_2, \phi_3)\\
\dot{\phi}_3 &= \omega_3 + \beta\sin(\phi_4 - \phi_3) + \beta^2 R_3(\phi_3, \phi_4)\\
\dot{\phi}_4 &= \omega_4 + \beta\sin(\phi_3 - \phi_4) + \beta^2 R_4(\phi_3, \phi_4),
\end{aligned}$$

where the higher-order terms in the coupling are periodic functions and

$$\hat{\omega}_{1,2} = \omega_{1,2} + \alpha^2\frac{\lambda(\omega_3 - \omega_2 - \omega_1)}{\lambda^2 + (\omega_3 - \omega_2 - \omega_1)^2} \quad \text{and} \quad \gamma \in \mathbb{R}.$$

For i). Since $\omega_3 - \omega_4 = O(\beta)$ we introduce $\Omega = (\omega_3 + \omega_4)/2$ and introduce the variables

$$\varphi_i = \phi_i - \Omega t, \quad \text{for} \quad i = 3, 4$$

to obtain

$$\dot{\varphi}_3 = \Delta_2 + \beta \sin(\varphi_4 - \varphi_3) + \beta^2 R_3(\varphi_3 + \Omega t, \varphi_4 + \Omega t).$$

A similar equation is obtained for φ_4. The variable φ_3 is slow and by the averaging theorem, we can introduce

$$g_3(x, y) = \frac{1}{T} \int_0^T R_3(x + \Omega t, y + \Omega t) dt$$

and because of the shift invariance $g_3(x + a, y + a) = g_3(x, y)$, there is G_3 such that $g_3(x, y) = G_3(x - y)$ by setting $a = -y$ and $G_3(u) = g_3(u, 0)$. A similar argument applies to the phase φ_4. Let's denote $\bar{\varphi}_3$ and $\bar{\varphi}_4$ the averaged variables. Then,

$$\dot{\bar{\varphi}}_3 = \Delta_2 - \beta \sin(\bar{\varphi}_3 - \bar{\varphi}_4) + \beta^2 G_3(\bar{\varphi}_3 - \bar{\varphi}_4) \tag{102}$$

$$\dot{\bar{\varphi}}_4 = -\Delta_2 - \beta \sin(\bar{\varphi}_4 - \bar{\varphi}_3) + \beta^2 G_4(\bar{\varphi}_4 - \bar{\varphi}_3). \tag{103}$$

Next, consider

$$\psi = \bar{\varphi}_3 - \bar{\varphi}_4$$

and notice that it follows

$$\dot{\psi} = 2\Delta_2 - 2\beta \sin\psi + \beta^2 G(\psi),$$

where $G(\psi) = G_3(\psi) - G_4(-\psi)$. This equation has a stable fixed point ψ^* when $|\Delta_2| < \beta$. By the averaging theorem persistence of hyperbolic equilibrium, there is a constant K such that for all times

$$|\varphi_3(t) - \varphi_4(t)| \leq K\beta$$

for the original phases. This implies that for the lift (choosing the appropriate branches)

$$|\hat{\varphi}_3(t) - \hat{\varphi}_4(t)| \leq \hat{K}\beta$$

and we obtain

$$|\Omega_3 - \Omega_4| = \lim_{t\to\infty} \frac{|\hat{\varphi}_3(t) - \hat{\varphi}_4(t)|}{t} = 0.$$

For ii). We consider

$$\phi = \phi_1 + \phi_2 - 2\phi_3.$$

Since $|\Delta_2| < \beta$ by the previous item, ϕ_3 is locked with ϕ_4 with frequency

$$\Omega_3 = \Omega_4 = \omega_4 + G(\beta)$$

and $G(\beta) < K\beta$. From the reduction,

$$\dot{\phi} = 2\hat{\Delta}_4 + 2F(\beta) - 2\alpha^2 \frac{\lambda}{\sqrt{\lambda^2 + \omega_3^2}} \sin(\phi + \gamma) + \alpha^4 R(\phi) \tag{104}$$

where we omitted an averaging step and

$$\hat{\Delta}_4 = \Delta_4 + \alpha^2 \frac{\lambda(\omega_3 - \omega_2 - \omega_1)}{\lambda^2 + (\omega_3 - \omega_2 - \omega_1)^2}$$

also notice that $F(\beta) = O(\beta^2)$. Moreover, since $\omega_3 - \omega_2 - \omega_1 = -\omega_4 - 2\Delta_4$ we obtain

$$\hat{\Delta}_4 = \Delta_4 - \alpha^2 \frac{\lambda(\omega_4 + 2\Delta_4)}{\lambda^2 + (\omega_4 + 2\Delta_4)^2}.$$

Ignoring the terms $O(\alpha^4)$ and rescaling time by a factor 2, we obtain

$$\dot{\phi} = \tilde{c}(\alpha) - \tilde{m}(\alpha)\sin(\phi - \gamma), \tag{105}$$

where

$$c(\alpha) = \Delta_4 + F(\beta) - \alpha^2 \frac{\lambda(\omega_4 + 2\Delta_4)}{\lambda^2 + (\omega_4 + 2\Delta_4)^2} \text{ and } m(\alpha) = \alpha^2 \frac{\lambda}{\sqrt{\lambda^2 + (\omega_4 + H(\beta))^2}}.$$

Now we are ready to apply Proposition 1. First, we notice that there is $K > 0$ such that

$$|F(\beta)| < K|\beta|, \;\; |G(\beta)| < K|\beta| \;\; \text{ and } |H(\beta)| < K|\beta|.$$

Then, we take

- $\beta = b\alpha^2$, for some small $b < b_* := \frac{\mu}{2K\omega_4^3}$, and $\beta = b_*\lambda^3\alpha^2$ using the Proposition 1.
- define $\rho := \frac{\mu}{32\omega_4^3}$, then $|\Delta_4| < \rho\lambda^4$, using the Proposition 1.

The constant $\mu > 0$ is defined in Proposition 1. Then there exist $0 < \alpha_1 < \alpha_2$ such that for all $\alpha \in (\alpha_1, \alpha_2)$, Eq (105) admits a stable equilibrium that will persist under small perturbations.

For iii). Introducing $\vartheta = \phi_1 - \phi_2$ we obtain

$$\dot{\vartheta} = \omega_1 - \omega_2 + R(\alpha, \phi_1, \phi_2, \phi_3),$$

where the remainder R is a periodic function and satisfies

$$|R(\alpha, \phi_1, \phi_2, \phi_3)| < M\alpha^3.$$

When $\omega_1 - \omega_2 > M\alpha^3_{\max}$ there is no locking. Similarly, for $\xi = \phi_1 - \phi_3$, we obtain

$$\dot{\xi} = \sigma + \alpha^3 G(\phi_1, \phi_2, \phi_3, \phi_4),$$

where

$$|\sigma| = |\omega_1 - \omega_3 + O(\alpha^2) + O(\lambda\alpha^2)| > \hat{K}\alpha^2_{\max}$$

where we used that $\beta = O(\alpha^2)$ and $|\omega_1 - \omega_3| > K_1\alpha^2_{\max}$. Moreover, let

$$K_2 = \sup|G(\phi_1, \phi_2, \phi_3, \phi_4)|$$

and take K_1 such that $\hat{K} > K_2$ thus $|\sigma - \alpha^3 G|$ is strictly positive. □

The triadic Motif

As we discussed earlier the triadic motif can be obtained by setting $\omega_3 = \omega_4$ for any $\beta > 0$. In this case, there will be locking between nodes 3 and 4 with effective frequency ω_3. And nodes 3 and 4 can be thought as a single unit. The equation for the triplet phase $\phi = \phi_1 + \phi_2 - 2\phi_3$ then reads as

$$\dot{\phi} = \Delta - \alpha^2_{\text{eff}}\sin(\phi + \gamma),$$

where

$$\Delta = \omega_1 + \omega_2 - 2\omega_3 + \alpha^2 \frac{\lambda(\omega_3 - \omega_2 - \omega_1)}{\lambda^2 + (\omega_3 - \omega_2 - \omega_1)^2}$$

and

$$\alpha^2_{\text{eff}} = \alpha^2 \frac{\lambda}{\sqrt{\lambda^2 + \omega_3^2}}.$$

The synchronization properties of ϕ in terms of existence of fixed points can be obtained via Proposition 1 with minor adjustments of the functions $c(\alpha)$ and $m(\alpha)$. Notice that α^2_{eff} will play the role of $m(\alpha)$ and ω_{eff} the role of $c(\alpha)$.

Asymmetry in the Arnold Tongue Lets obtain a first-order approximation of the boundary of the sync Tongue. First, we consider the following quantities

$$2\omega_3 = \omega_1 + \omega_2 + x, \qquad \beta := \frac{\omega_1 + \omega_2}{2}, \qquad \omega_3 = \beta + \frac{x}{2}, \qquad S := \sqrt{\lambda^2 + \beta^2},$$

where x now is a detuning parameter. Lets also introduce the effective quantities

$$\Delta(x) := \frac{x}{2} - \beta, \qquad \Sigma(x) := \beta + \frac{x}{2},$$

and thus

$$\omega_{\text{eff}}(x, \alpha) = -x + \alpha^2 \, c_1(x), \qquad \alpha^2_{\text{eff}}(x, \alpha) = \alpha^2 \, c_2(x),$$

where the functions are

$$c_1(x) = \frac{\lambda \, \Delta(x)}{\lambda^2 + \Delta(x)^2}, \qquad c_2(x) = \frac{\lambda}{\sqrt{\lambda^2 + \Sigma(x)^2}}.$$

Boundary condition of the Arnold Tongue gives

$$\big|\omega_{\text{eff}}(x, \alpha)\big| = \big|\alpha^2_{\text{eff}}(x, \alpha)\big| \quad \Longleftrightarrow \quad \big|-x + \alpha^2 c_1(x)\big| = \alpha^2 c_2(x).$$

The algebraic branches then are given as

$$\alpha^2_{\pm}(x) = \frac{x}{D_{\pm}(x)}, \qquad \text{where } D_{\pm}(x) := c_1(x) \pm c_2(x)$$

with

$$c_1(0) = -\frac{\lambda\beta}{S^2}, \qquad c_2(0) = \frac{\lambda}{S}, \qquad d_0^{\pm} := D_{\pm}(0) = -\frac{\lambda\beta}{S^2} \pm \frac{\lambda}{S} = \frac{\lambda}{S^2} \, (\pm S - \beta).$$

Thus, we obtain the explicit one-sided formulas

$$\alpha^2_{\pm}(x) = \frac{x}{d_0^{\pm}} + O(x^2) \quad \Longrightarrow \quad \alpha_{\pm}(x) \; \approx \; \sqrt{\frac{x}{d_0^{\pm}}} \text{ valid when } x \, d_0^{\pm} > 0$$

and

$$\alpha_{+}(x) \; \approx \; \sqrt{\frac{x \, S^2}{\lambda \, (S - \beta)}} = \sqrt{\frac{x(\lambda^2 + \beta^2)}{\lambda\big(\sqrt{\lambda^2 + \beta^2} - \beta\big)}} \quad (x > 0)$$

$$\alpha_{-}(x) \; \approx \; \sqrt{\frac{-x \, S^2}{\lambda \, (S + \beta)}} = \sqrt{\frac{(-x)(\lambda^2 + \beta^2)}{\lambda\big(\sqrt{\lambda^2 + \beta^2} + \beta\big)}} \quad (x < 0).$$

We have a cusp and because the slopes d_0^+ and d_0^- are different we obtain an asymmetry in the tongue. In Supplementary Figure 2 we provide the boundary of the synchronization tongue by solving $|\omega_{\text{eff}}| = |\alpha^2_{\text{eff}}|$ numerically for $\lambda = 0.44$, $\omega_1 = 1.2$ and $\omega_2 = 1.13$.

SUPPLEMENTARY NOTE IV: EXPERIMENTAL HYPERLOCKING

In this section, we describe the details about the experimental setup and the dynamical behavior of the oscillators without coupling.

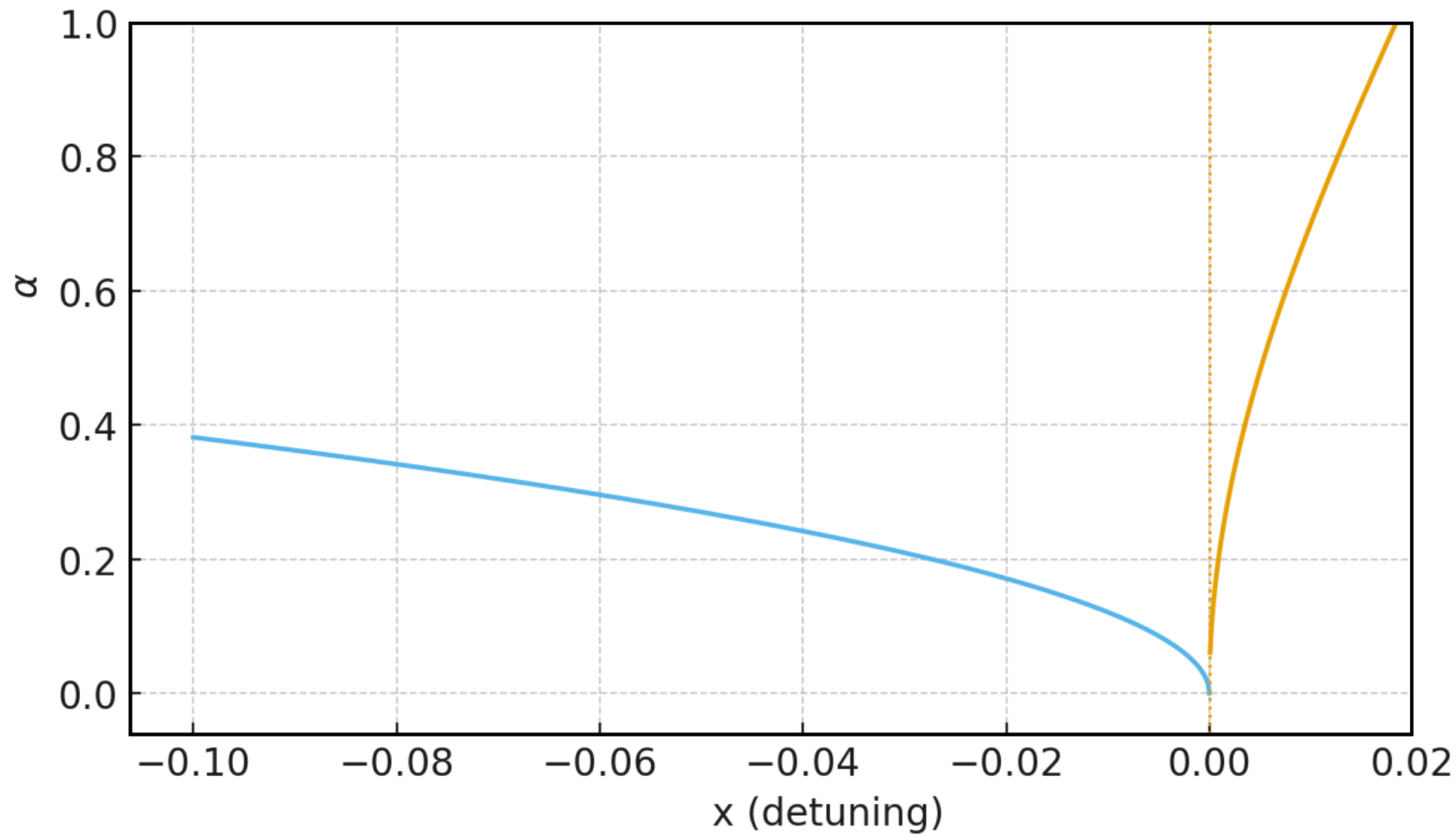

Supplementary Figure 2. **Asymmetry in the boundary of the Arnold Tongue**. We obtain the blue and orange curves by solving $|\omega_{\text{eff}}| = |\alpha^2_{\text{eff}}|$ numerically.

Experimental setup and data processing

The experiments were carried out in a standard three-electrode electrochemical cell. The cell consists of an array of nickel wires as working electrode, a platinum-coated titanium rod as a counter electrode, and a Hg/Hg_2SO_4/sat. K_2SO_4 as reference and auxiliary reference electrodes. The electrolyte was a 3.0 M sulfuric acid solution maintained at a constant temperature of 10°C. The electrode array consisted of three 1-mm diameter nickel wires embedded in epoxy with a spacing of 3 mm. A multichannel potentiostat (Gill-IK64, ACM Instruments) interfaced with a real-time LabVIEW controller measured the potential (with respect to the auxiliary reference electrode) and set the circuit potential ($V_{0,k}$) of the working electrodes individually.

The individual electrode potentials (E_k) were recorded, rescaled and offset corrected

$$\tilde{E}_k = O_k[E_k - o_k] \tag{106}$$

where o_k and O_k are the time-averaged electrode potential and amplitude rescaling factor, respectively. The factors, $O_k = 0.94\ \text{V}^{-1}$, $1\ \text{V}^{-1}$, $0.94\ \text{V}^{-1}$ were applied to counter the different amplitudes of the oscillators. Data was acquired at a rate of 200 Hz. The three nickel wires were connected to 1.00 kΩ, 0.80 kΩ and 0.90 kΩ resistors, respectively.

To determine the phase of each oscillator, we employed Hilbert transform. The Hilbert transform of the time series is defined by

$$H(E_k(t)) = \frac{1}{\pi}\,\text{PV}\int_{-\infty}^{\infty} \frac{E_k(\tau) - \langle E_k \rangle}{t - \tau}\, d\tau \tag{1}$$

where PV implies that the integral should be evaluated in the sense of Cauchy principal value and $\langle\rangle$ denotes the temporal average. The phase can be obtained by

$$\phi(t) = \arctan\left(\frac{H(E_k(t))}{E_k(t) - \langle E_k \rangle}\right) \tag{107}$$

From the phase, the frequency of an oscillator is derived as

$$\omega = \frac{1}{2\pi}\left\langle \frac{d\phi}{dt} \right\rangle \tag{108}$$

Behavior without coupling

In the nickel elctrodissolution system, electrode potential oscillations arise via a Hopf bifurcation as the circuit potential is increased. The Hopf point and the natural frequency of each oscillator depend on the magnitude of

circuit potential applied and external resistance connected to a wire. The offset circuit potential to each oscillator was established 50 mV above the Hopf point ($V_{0,1}$ = 1100 mV, $V_{0,2}$ = 1090 mV, $V_{0,3}$ = 1097 mV). For experiments with positive triplet detuning, the natural frequencies of the oscillators were adjusted to have values of $\omega_1 \approx 448$ Hz, $\omega_2 \approx 460$ Hz, $\omega_3 \approx 451$ Hz owing to the different individual resistances used for each. Without coupling, we observed that oscillators 1 and 3 have slightly larger amplitude than oscillator 2. Therefore, to counter the effect of different amplitudes, an amplitude rescaling factor was used for oscillators 1 and 3 as $O_{1,3} = 0.94$ V^{-1}. The electrode potentials are corrected for offset $o_1 = 0.97$ V, $o_2 = 0.98$ V, $o_3 = 0.97$ V.

Robustness to delays

In the manuscript, a delay of $\tau \approx T/2$ was applied to demonstrate hyperlocking. In order to confirm robustness of hyperlocking to delays, we performed two additional experiments with somewhat shorter and longer delays than $T/2$. Supplementary Figure 3 shows two cases of experimental hyperlocking, one with 2.5 % lower (Supplementary Figure 3(a)) and one with 3.9 % higher (Supplementary Figure 3) delays than $\tau = T/2$ For both experiments, hyperlocking was observed as the triplet phase difference (ψ) remained bounded.

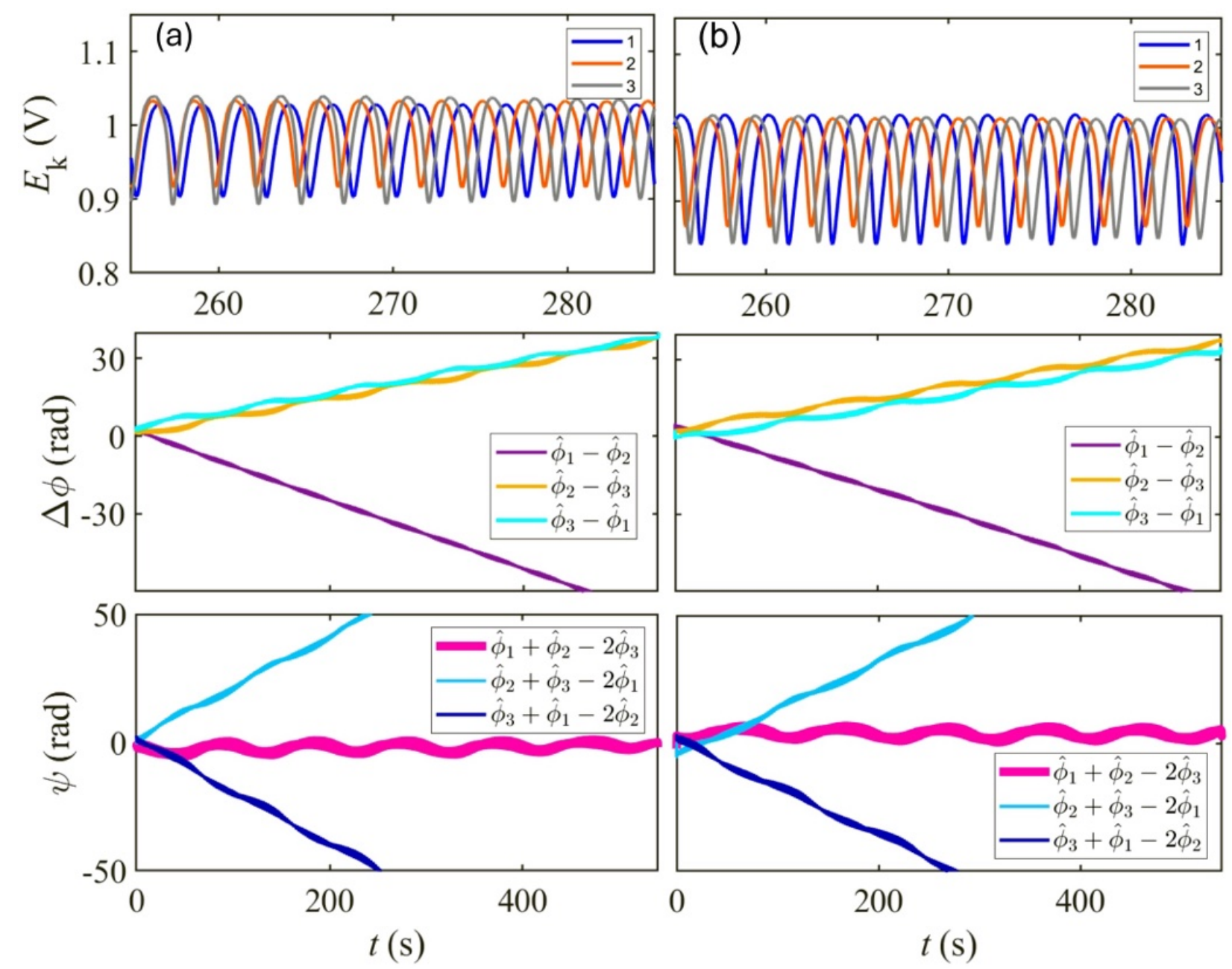

Supplementary Figure 3. **Hyperlocking in experiments is robust to coupling delays.** Top to bottom: Electrode potential time series, evolution of pairwise and triplet phase differences with time, respectively at a coupling strength K = -0.2 V^{-1}. (a) τ =0.975 (T/2)= 1.18 s, T=2.42 s. $V_{0,1}$ = 1090 mV, $V_{0,2}$ = 1090 mV, $V_{0,3}$ = 1095 mV, and, R_1 = 0.85 kΩ, R_2 = 0.80 kΩ, R_3 = 0.80 kΩ. (b) τ = 1.039 (T/2)= 1.21 s, T =2.33 s. $V_{0,1}$ = 1100 mV, $V_{0,2}$ = 1093 mV, $V_{0,3}$ = 1090 mV, and, R_1 = 1.00 kΩ, R_2 = 0.80 kΩ, R_3 = 0.90 kΩ. For both the experiments, uncoupled triplet detuning was, $\Delta\omega = \omega_1 + \omega_2 - 2\omega_3 = 6$ mHz.

SUPPLEMENTARY NOTE V: VAN DER POL OSCILLATORS

In this section, we show that hyperlocking can also be found in a system of coupled Van der Pol oscillators. We use in the general triadic system the oscillator

$$f(x_j, y_j) = \begin{pmatrix} y_j \\ \mu(1 - x_j^2)y_j - \omega_j^2 x_j \end{pmatrix} \tag{109}$$

with the interaction functions

$$h((x_1, y_1), (x_2, y_2)) = \begin{pmatrix} x_2 - x_1 \\ y_2 + y_1 \end{pmatrix}, \qquad g(x, y) = \begin{pmatrix} x & -y \\ y & x \end{pmatrix}. \tag{110}$$

We choose $\omega_1 = 0.95$, $\omega_2 = 1.05$, $\omega_3 = 1.045$, the coupling strength $\alpha = 0.05$ and the nonlinearity parameter $\mu = 0.3$, we observe stable triplet locking, see Supplementary Figure 4:

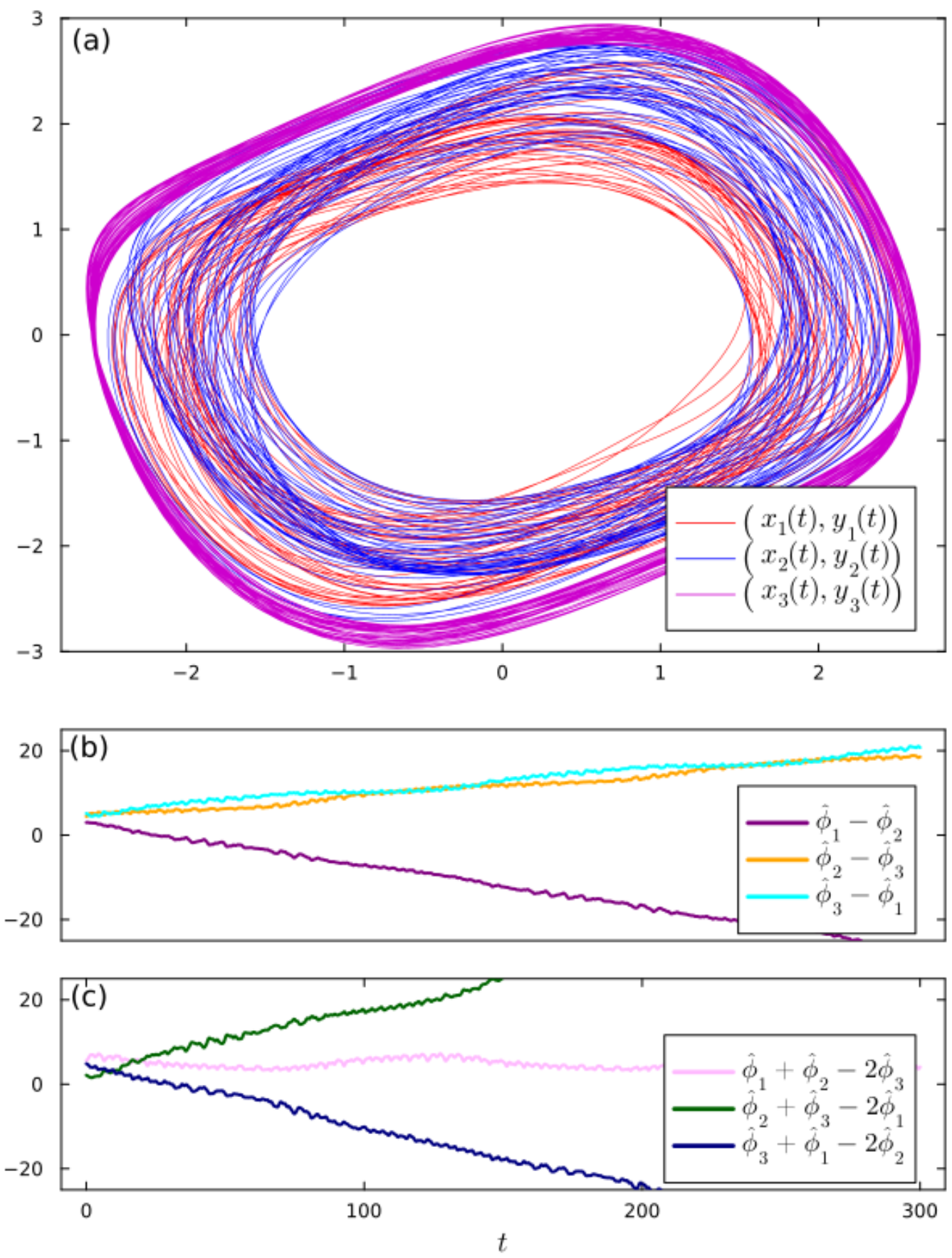

Supplementary Figure 4. **Triplet locking of a triadic network of Van-der-Pol oscillators.** Numerical solution of system (109)–(110). Panel (a): trajectories of the oscillators in the (x, y)-plane. Panel (b): pairwise phase differences (no pairwise locking). Panel (c): triplet phase differences. The triplet combination $\phi_1 + \phi_2 - 2\phi_3$ (blue) remains bounded, indicating the hyperlocking of this phase triplet.

[1] E. Nijholt, J. L. Ocampo-Espindola, D. Eroglu, I. Z. Kiss, and T. Pereira, Emergent hypernetworks in weakly coupled oscillators, Nature communications **13**, 4849 (2022).
