# Supplementary material for: Hypernetworks induce stable hyperlocking
Source: Nat Commun. 2026 Jun 24;17:7947. doi: 10.1038/s41467-026-74556-1 (PMC13448840; doi:10.1038/s41467-026-74556-1)
Supplement: Supplementary file 2 [file 41467_2026_74556_MOESM2_ESM.pdf]

nature portfolio

Peer Review File

Hypernetworks induce stable hyperlocking

Corresponding Author: Professor Tiago Pereira

This file contains all reviewer reports in order by version, followed by all author rebuttals in order by version.

Version 0:

Reviewer comments:

Reviewer #1

(Remarks to the Author)
The authors the dynamics of a hypernetwork made of three-oscillator with resonant frequencies. Combined with an adequate coupling function, the system displays what the authors call "hyperlocking": triplet phase-locking but no pairwise phase phase-locking. With analytics (phase reduction and normal forms), the authors explain why this is the case, and confirm their predictions with numerics and chemical experiments. They argue that this shows that triadic interactions can give rise to fundamentally new dynamical regimes

I liked the paper: the topic is of interest and timely, the analysis appears to be sound, and it is well written. The application of phase reduction to explain the triplet synchrony is interesting, and experiments are always a plus. Yet, some important claims seem to not be supported by the results presented, as detailed below. This would need to be added or at the very least clarified. Because of this, I cannot recommend publication of the manuscript in its current form.

MAJOR COMMENTS

- Some important claims do not match the results presented, or should be made more precise.

For example, the abstract states "our findings uncover a new synchronization regime intrinsic to higher-order interactions". This is a key message of the manuscript, a bit later early page 2: "Our findings show that triadic interactions can give rise to fundamentally new dynamical regimes". But Ref. [28] (Kralemann et al. 2013) already presents a system, a ring of three diffusively pairwise coupled Rayleigh oscillators, that also displays hyperlocking (triplet synchrony but no pairwise synchrony). So unless I am missing something, this synchronization regime is already known and is not intrinsic to higher-order interactions. Can you comment on this?

Another example: the abstract states "open new directions for controlling real-world complex dynamics", and similarly early page 2 "design coupling functions that realize desired hypernetwork states, thus offering control over collective dynamics." But only two specific choices of coupling function are shown (one in Eq 4 leading to hyperlocking, and another in Fig 5 that leads to pairwise locking) without real explanation of why they do what they do. So how did the authors choose these functions?
The introduction also says "we find open sets of coupling functions", and on page 5 "the class of coupling functions we consider", but again, only two specific functions are shown. Is there something more general about this in the SM that is not referenced in the main text?

On page 4, the authors write "In the Supplementary Note III, we provide examples of larger network architectures that contain such triadic structures and exhibit hyperlocking behavior. These cases illustrate that hyperlocking can emerge in extended systems through the embedding of triadic interactions, highlighting the broader relevance of this phenomenon for the dynamics of higher-order networks." I think showing how this and other motifs can impact dynamics of larger networks can indeed be very interesting and make the paper stronger. But I did not find this in SN III, which considers an extended motif of 4 nodes. I couldn't find anything related to how this or other motifs impact larger networks. Please clarify or remove this claim. I think that if the authors do have results on this, it might even be worth including them in the main text.

- The authors write above the Conclusion:"Our results highlight that the boundary between network and hypernetwork dynamics is not clear-cut. Only the interplay between network structure, coupling functions, and resonances decides the true nature of the emergent dynamical interactions in the system." and in the Conclusion: "Our results position triadic interactions as a new paradigm for complex dynamics, where structure and function fuse to shape emergent behaviour." But there is already work showing that the interplay between the network structure and coupling functions, and that it can sometimes reduce to pairwise interactions, for example :

- Neuhäuser, L., Scholkemper, M., Tudisco, F., & Schaub, M. T. (2024). Learning the effective order of a hypergraph dynamical system. Science Advances, 10(19), eadh4053.
- Neuhäuser, L., Mellor, A., & Lambiotte, R. (2020). Multibody interactions and nonlinear consensus dynamics on networked systems. Physical Review E, 101(3), 032310.

So, the results of the present manuscript should be framed more precisely and contextualized more with existing literature.

- The text is nicely written and clear, but it feels like it was written for a shorter format than allowed by Nature Communications. This is not a problem in itself, but the manuscript sometimes goes too fast over important steps and results in the main text, referencing the Supplementary Notes. I agree that the details of the proofs should not be in the main text, but giving a bit more explanations on key steps in the main would help the reader.

For example, the two paragraphs above Eqs 5-7 give some brief idea of the analytics, but would benefit from slightly more explanations, maybe sometimes giving examples of terms described ("all terms appearing in first order are non-resonant"). Another example include the theory/discussion of general coupling functions, and other motifs and larger networks, as mentioned above.

MINOR COMMENTS

- report the coupling function used in Fig 5 also in the text (not only in caption).
- the Supplementary Notes are often very mathematical, and would benefit from a bit more text here and there to link the proofs and theorem, and explain the meaning and importance of the results to the diverse readership of Nature Communications.

Reviewer #2

(Remarks to the Author)
The authors studied a network of oscillators with higher order interactions where the coupling is mediated by a triadic hypernetwork motif. In this case, the coupling between nodes 1 and 2 is mediated by node 3, and the interactions in the system cannot be fully reduced to pairwise interactions. The authors describe a new synchronization phenomenon in this network with higher order interactions, where the system displays a “triplet synchronization”. This occurs when the oscillators have nearly identical natural frequencies, and pairwise synchronization is found to not occur. The authors then developed an analytical study to explain this phenomenon and obtain the parameters/coupling functions for which triplet synchronization is possible. They then confirm the predictions from the analytical study through numerical simulations and also chemical experiments.

Overall, the manuscript addresses an interesting and timely topic. It is always satisfying to see a study that integrates analytical theory, numerical modeling, and experimental validation. However, I have several important questions about the work and significant concerns with the current version of the manuscript. As it stands, I am not fully convinced that it meets the high standards required for publication in Nature Communications.

Below, I list my concerns with the manuscript. I hope this is helpful.

(1) While the manuscript contains analytical, numerical, and experimental results, these consider very small networks, i.e. 3 nodes. The authors highlight the importance of higher order interactions in real-world systems in the second paragraph of the introduction, but their results are constrained to very small networks, which raises the questions about applicability. The authors only quickly note in the manuscript that this triadic motif can be part of a larger network and that hyperlocking can exist in extended systems and refer to the Supplement, which does not offer a lot of examples. Overall, I agree with the authors about the importance of higher order interactions for real-world systems, but I think it’s crucial the authors show and/or discuss how their results can actually be applied or how they contribute to real-world systems. Oscillator networks are important for power-grids, neural systems, and neural networks. How do the results presented here contribute to real-world systems?

(2) One of the main points of the manuscript in my opinion is the fact that the authors found a dynamical behavior that is exclusive for higher-order networks. This is, in fact, highlighted by the authors in the abstract, introduction, and conclusion. While this is interesting, this is not necessarily new. Higher order interactions have been studied for many years now (https://doi.org/10.1038/s42005-021-00695-0; https://doi.org/10.1016/j.physrep.2023.04.002); and several authors have already shown “exclusive dynamical behavior” in these systems. For example, higher order interactions have been shown to

enable chimera states to appear in networks without phase-lag (https://doi.org/10.1103/PhysRevE.105.L042202; https://doi.org/10.1016/j.chaos.2023.113325); or to induce synchronization in parameter regimes in which pairwise interactions wouldn't (https://doi.org/10.1103/PhysRevLett.127.258301); induce solitary states (https://doi.org/10.1103/xtgc-f766); and also to change stability properties in oscillator networks (https://arxiv.org/abs/2510.09387); to cite a few.

(3) The current manuscript is written pretty much as a letter. I believe there is a missed opportunity here to expand more on the text and offer the reader more details about the work and its implications. Particularly, the current version is lacking a "discussions" section, in my opinion. The manuscript only offers a short paragraph under "Conclusion", but no extended discussion about the implications of the results in the literature of higher-order interactions, or even possible applications in real-world systems. Since Nature Communications doesn't have a specific page limit, I believe it would strengthen the manuscript a lot if the authors added more material and discussions to the manuscript.

(4) What's the reason behind the choice for Stuart-Landau oscillators? How does this choice match with the empirical setup of chemical oscillators? Are the results also true for other oscillator networks? In the supplement, the authors show similar results for Van-der-Pol oscillators, but I'm curious to what extent this can be observed in different models, e.g. Kuramoto.

(5) The color-code in the figures is a little bit confusing. For example, in Fig. 1, even though the colors are the same in panels (a), (b), and (c), as far as I understood, they are not related. Blue in Fig. 1 (a) represents the (real part) of the trajectory of node 1, but in (b) it represents the difference in phase between nodes 1 and 2, and in (c) it represents the triplet synchronization that is explored in the paper. I'd recommend the authors to re-think the color scheme. Even though there is nothing necessarily wrong with that, my brain immediately tried to draw connections that are not there.

(6) What is special about the triplet synchronization (\phi_1 + \phi_2 - 2\phi_3)? While I understand the authors observed this phenomenon, and that this is not observed in networks with pairwise interactions only (for certain coupling choices), I still can't quite grasp what is important about this behavior. For example, why is \phi_1 + \phi_2 - 2\phi_3 more important than \phi_3 + \phi_1 - 2\phi_2? Or even, why is hyperlocking more important than standard pairwise synchronization (both phase locking and phase synchronization)? Phase locking and phase synchronization have strong implications in real-world systems; they have led to important theoretical insights in neuroscience (e.g. Communications through Coherence theory), and also direct applications in power-grid studies. While I enjoyed reading the current manuscript, I think it lacks on this point. This goes back to my comments about having more discussions in the paper, about the results and the implications.

(7) The empirical setup is very interesting and the results impressive. At the same time, however, the system has now delayed interactions, so it's not exactly what is described in Eq. (1). It's well-known that delayed interactions can disrupt synchronization in coupled networks, and induce more complicated patterns (e.g. waves, chimeras, clusters). I think there should be more discussion in the manuscript about how this delayed system can be seen as a higher-order network. Also, as a minor point, Fig. 4 could be bigger.

(8) In Fig. 5 the authors show that different choices for coupling can lead to pairwise locking in their system. I believe this is a quite important point, and it goes back to my previous comments. Why is hyperlocking phenomenon important, and what's the relevance of the type of coupling that leads to this new dynamical phenomenon? The authors only briefly mention Fig. 5 and say that they have results in the supplement about it. But as I mentioned previously, Nature Communications doesn't have a strict limit of 5 pages, so why not add the discussions in the main text? I think this would strengthen the manuscript and give the reader enough information/material to better appreciate the results and their implications.

Reviewer #3

(Remarks to the Author)
This manuscript presents a clear and timely study introducing the concept of hyperlocking, a novel synchronization regime emerging from a triadic coupling motif. The authors combine analytical derivations based on phase reduction and normal form theory with numerical simulations and an experimental realization using electrochemical oscillators. The work is technically solid, clearly presented, and likely to be of interest to researchers working on nonlinear dynamics and complex systems.
A few conceptual clarifications would strengthen the manuscript. In the introduction, the authors refer to higher-order interactions in general terms but move directly to a triadic motif, without stating that this is a specific subclass of higher-order interactions. In triadic interactions, one node regulates or modulates the coupling between two others, rather than participating symmetrically in a group interaction.
In the present model, the coupling term $g(z_3)h(z_2,z_1)$ introduces a hierarchical dependency rather than a symmetric three-body interaction like $h(z_1,z_2,z_3)$. The system captures a specific mechanism of higher-order modulation, but not the full symmetry and mutual dependence of standard higher-order dynamical frameworks. While hyperlocking is intriguing, it would help if the authors discussed whether similar behavior could also emerge in systems with simplicial or symmetric three-body coupling, or if it is specific to this coupling choice. This discussion would help readers appreciate the generality of the effect and its broader relevance to higher-order dynamical systems.
Finally, the electrochemical setup is presented as an experimental validation of the theoretical model, but the triadic coupling is digitally implemented rather than arising intrinsically from the physical system. In this sense, the experiment functions as a controlled hardware realization of the proposed dynamics, rather than a self-organized instance of higher-order coupling. Clarifying this distinction would improve the presentation and prevent possible overinterpretation of the experimental results.

Version 1:

Reviewer comments:

Reviewer #1

(Remarks to the Author)
I thank the authors for the revisions which improve the manuscript. In particular, I think the addition of the larger random networks adds a lot. I still have a few minor concerns detailed below:

"triplet locking is reported near triplet resonances of the form $\omega1 + \omega2 - 2 \omega3 = 0$, where no pairwise resonances are present (...) hyperlocking occurs near a full resonance $\omega1 \approx \omega2 \approx \omega3$,"

> Thank you, this clarifies it. I would however be careful with how hyperlocking is phrased in the manuscript. The abstract reads "Our findings uncover a new synchronization regime intrinsic to higher-order interactions" but from what I understand, the triplet locking ([28]) and your hyperlocking are the same "synchronization regime" in the sense that, from experimental time series, if one doesn't know the natural frequencies of the oscillators, there is no way of distinguishing the two dynamics: in both cases, triplet locking is present but pairwise locking is not. So it is not really a new regime. The two cases are then different "mechanisms" to obtain the same "stable regime". In fact, the conclusion uses this phrasing: "Hyperlocking is a general synchronization mechanism in networks with higher-order interactions".

"The coupling functions shown in Eq. (4) and Fig. 5 were not chosen ad hoc. They are derived from our normal form approach, so we already reverse-engineered them."

> I thank the authors for the explanation. However, I don't see this mentionned in the manuscript? This needs to be mentioned, problably already near Eq 4.

"The hyperlocked state is hyperbolic, as shown in Supp. Mat. III. As a consequence, it is structurally stable under small smooth perturbations."

> Similarly, I thank the authors for the explanation but don't find it in the manuscript or the SM. Please mention this somewhere in the main text, it is an important but technical fact that not enough readers of the journal will have knowledge of.

"We have expanded the text to include more simulations, examples, and further explanations, including hyperlocking in random triadic networks (pages 6 and 7)."

> This is very insightful, thank you. This new text also drew my attention to the fact that the motif considered contains *directed* interactions. I think this does not diminish the results, but it should be made more explicit in the abstract/intro/conclusion because most of the literature on synchronization on higher-order networks considers undirected interactions, and the results of the present manuscript shows a nice effect of directed interactions which, a priori, wouldn't have any analogous effect for undirected interactions. Based on this, it might also be worth briefly mentioning studies on the sync with higher order directed interactions and what we know from them. For example:

Gallo, L., Muolo, R., Gambuzza, L. V., Latora, V., Frasca, M., & Carletti, T. (2022). Synchronization induced by directed higher-order interactions. Communications Physics, 5(1), 263.

Reviewer #2

(Remarks to the Author)
I appreciate the authors' efforts in revising the manuscript. In my view, the new version provides a more balanced presentation of the work, particularly in how it frames the broader context, engages with prior studies on hypernetwork dynamics, and clarifies the novelty of the current contribution. I also found the new discussion section helpful and engaging. Overall, I believe the manuscript has improved substantially compared to the original submission.

I have one remaining point that I would appreciate further clarification on. Based on the authors' response and on the revised manuscript, time delays are necessary for the emergence of hyperlocking in the experimental setup. Without delays, the network would exhibit "trivial in-phase pairwise synchrony". As I understand it, the authors argue that the delayed interactions effectively mimic the triadic motif coupling. I might be missing something here, but it seems plausible that different forms of delayed interactions could also generate an effective triadic motif (perhaps with different parameter regimes), yet not necessarily lead to hyperlocking. Is that correct? If so, how does this align with the framework presented in the manuscript and with the claim that the triadic motif is necessary for hyperlocking? More specifically, I would appreciate clarification on two related points: (1) Is it possible to obtain hyperlocking in a pairwise network with appropriately structured delays (for example, distance-dependent delays)? (2) I am not fully convinced that the empirical results demonstrate robustness to delays. In the current setup, delays appear to be the key mechanism that enables the triadic motif coupling; without them, hyperlocking does not occur. To be considered "robust" I'd expect the behavior to exist in spite of a given factor (delays in this case), not because of it.

Version 2:

Reviewer comments:

Reviewer #2

(Remarks to the Author)
I appreciate the authors' response and revisions on the manuscript. The authors addressed my concerns and I'm happy to recommend publication.

Open Access This Peer Review File is licensed under a Creative Commons Attribution 4.0 International License, which permits use, sharing, adaptation, distribution and reproduction in any medium or format, as long as you give appropriate credit to the original author(s) and the source, provide a link to the Creative Commons license, and indicate if changes were made.
In cases where reviewers are anonymous, credit should be given to 'Anonymous Referee' and the source.
The images or other third party material in this Peer Review File are included in the article's Creative Commons license, unless indicated otherwise in a credit line to the material. If material is not included in the article's Creative Commons license and your intended use is not permitted by statutory regulation or exceeds the permitted use, you will need to obtain permission directly from the copyright holder.
To view a copy of this license, visit https://creativecommons.org/licenses/by/4.0/

REVIEWER COMMENTS

Reviewer #1 (Remarks to the Author):

The authors the dynamics of a hypernetwork made of three-oscillator with resonant frequencies. Combined with an adequate coupling function, the system displays what the authors call "hyperlocking": triplet phase-locking but no pairwise phase phase-locking. With analytics (phase reduction and normal forms), the authors explain why this is the case, and confirm their predictions with numerics and chemical experiments. They argue that this shows that triadic interactions can give rise to fundamentally new dynamical regimes

I liked the paper: the topic is of interest and timely, the analysis appears to be sound, and it is well written. The application of phase reduction to explain the triplet synchrony is interesting, and experiments are always a plus. Yet, some important claims seem to not be supported by the results presented, as detailed below. This would need to be added or at the very least clarified. Because of this, I cannot recommend publication of the manuscript in its current form.

We thank the referee for the careful and thoughtful evaluation of our manuscript and for the time devoted to the report. We are grateful for the referee's recommendation, as well as for the constructive comments provided. Below, we respond point by point to the referee's comments. We have clarified and, where necessary, strengthened the arguments supporting our main claims, as detailed in the responses below, and revised the manuscript accordingly.

MAJOR COMMENTS

- Some important claims do not match the results presented, or should be made more precise.

For example, the abstract states "our findings uncover a new synchronization regime intrinsic to higher-order interactions". This is a key message of the manuscript, a bit later early page 2: "Our findings show that triadic interactions can give rise to fundamentally new dynamical regimes". But Ref. [28] (Kralemann et al. 2013) already presents a system, a ring of three diffusively pairwise coupled Rayleigh oscillators, that also displays hyperlocking (triplet synchrony but no pairwise synchrony). So unless I am missing something, this synchronization regime is already known and is not intrinsic to higher-order interactions. Can you comment on this?

We agree with the referee that further explanation can clarify the hyperlocking phenomenon. For this, we included a discussion on page 2 and a discussion of Ref. [28] on page 3. A short summary is that the triplet locking is reported near triplet resonances of the form $\omega_1 + \omega_2 - 2\omega_3 = 0$, where no pairwise resonances are present. The triplet locking is explained by the classical averaging methods. In contrast, hyperlocking occurs near a full resonance $\omega_1 \approx \omega_2 \approx \omega_3$, where both pairwise and triplet locking could be possible, but for specific choices of the coupling function, the pairwise one is dynamically forbidden.

Another example: the abstract states "open new directions for controlling real-world complex dynamics", and similarly early page 2 "design coupling functions that realize desired hypernetwork states, thus offering control over collective dynamics." But only two specific choices of coupling

function are shown (one in Eq 4 leading to hyperlocking, and another in Fig 5 that leads to pairwise locking) without real explanation of why they do what they do. So how did the authors choose these functions?

> The coupling functions shown in Eq. (4) and Fig. 5 were not chosen ad hoc. They are derived from our normal form approach, so we already reverse-engineered them. The statement about "control" is meant in this sense: the theory provides a design principle for shaping collective dynamics by selecting coupling functions that enhance or suppress specific resonant interactions. While we illustrate this principle with two examples, the mechanism applies generally. In fact, we now provide examples of random networks on page 7.

The introduction also says "we find open sets of coupling functions", and on page 5 "the class of coupling functions we consider", but again, only two specific functions are shown. Is there something more general about this in the SM that is not referenced in the main text?

> The hyperlocked state is hyperbolic, as shown in Supp. Mat. III. As a consequence, it is structurally stable under small smooth perturbations. In particular, any coupling function lying in a neighborhood (in the C^r-topology) of the explicit examples shown in the manuscript will also support hyperlocking.

On page 4, the authors write "In the Supplementary Note III, we provide examples of larger network architectures that contain such triadic structures and exhibit hyperlocking behavior. These cases illustrate that hyperlocking can emerge in extended systems through the embedding of triadic interactions, highlighting the broader relevance of this phenomenon for the dynamics of higher-order networks." I think showing how this and other motifs can impact dynamics of larger networks can indeed be very interesting and make the paper stronger. But I did not find this in SN III, which considers an extended motif of 4 nodes. I couldn't find anything related to how this or other motifs impact larger networks. Please clarify or remove this claim. I think that if the authors do have results on this, it might even be worth including them in the main text.

> We added results on random networks with triadic interactions on Pages 6 and 7.

- The authors write above the Conclusion:"Our results highlight that the boundary between network and hypernetwork dynamics is not clear-cut. Only the interplay between network structure, coupling functions, and resonances decides the true nature of the emergent dynamical interactions in the system." and in the Conclusion: "Our results position triadic interactions as a new paradigm for complex dynamics, where structure and function fuse to shape emergent behaviour." But there is already work showing that the interplay between the network structure and coupling functions, and that it can sometimes reduce to pairwise interactions, for example :

- Neuhäuser, L., Scholkemper, M., Tudisco, F., & Schaub, M. T. (2024). Learning the effective order of a hypergraph dynamical system. Science Advances, 10(19), eadh4053.
- Neuhäuser, L., Mellor, A., & Lambiotte, R. (2020). Multibody interactions and nonlinear consensus dynamics on networked systems. Physical Review E, 101(3), 032310.

So, the results of the present manuscript should be framed more precisely and contextualized more with existing literature.

> We included this discussion in the conclusions on pages 6 and 7.

- The text is nicely written and clear, but it feels like it was written for a shorter format than allowed by Nature Communications. This is not a problem in itself, but the manuscript sometimes goes too fast over important steps and results in the main text, referencing the Supplementary Notes. I agree that the details of the proofs should not be in the main text, but giving a bit more explanations on key steps in the main would help the reader.

We have expanded the text to include more simulations, examples, and further explanations, including hyperlocking in random triadic networks (pages 6 and 7).

For example, the two paragraphs above Eqs 5-7 give some brief idea of the analytics, but would benefit from slightly more explanations, maybe sometimes giving examples of terms described ("all terms appearing in first order are non-resonant"). Another example include the theory/discussion of general coupling functions, and other motifs and larger networks, as mentioned above.

We have expanded the discussion around Eqs. (5)–(7), while keeping technical details in the Supplementary Notes. We have added brief clarifying remarks in the sections on general coupling functions and on extensions to other motifs and larger networks to improve accessibility without overloading the main text.

MINOR COMMENTS

- report the coupling function used in Fig 5 also in the text (not only in caption).

Done

- the Supplementary Notes are often very mathematical, and would benefit from a bit more text here and there to link the proofs and theorem, and explain the meaning and importance of the results to the diverse readership of Nature Communications.

We amended the Notes following the referee´s suggestion.

Reviewer #2 (Remarks to the Author):

The authors studied a network of oscillators with higher order interactions where the coupling is mediated by a triadic hypernetwork motif. In this case, the coupling between nodes 1 and 2 is mediated by node 3, and the interactions in the system cannot be fully reduced to pairwise interactions. The authors describe a new synchronization phenomenon in this network with higher order interactions, where the system displays a "triplet synchronization". This occurs when the oscillators have nearly identical natural frequencies, and pairwise synchronization is found to not occur. The authors then developed an analytical study to explain this phenomenon and obtain the parameters/coupling functions for which triplet synchronization is possible. They then confirm the predictions from the analytical study through numerical simulations and also chemical experiments.

Overall, the manuscript addresses an interesting and timely topic. It is always satisfying to see a study that integrates analytical theory, numerical modeling, and experimental validation. However, I have several important questions about the work and significant concerns with the current version of the manuscript. As it stands, I am not fully convinced that it meets the high standards required for publication in Nature Communications.

> We thank the Referee for their careful reading of the manuscript and for their detailed and constructive report. We appreciate their recognition of the timeliness of the topic and the integration of analytical, numerical, and experimental approaches, and we address their concerns in detail below.

Below, I list my concerns with the manuscript. I hope this is helpful.

(1) While the manuscript contains analytical, numerical, and experimental results, these consider very small networks, i.e. 3 nodes. The authors highlight the importance of higher order interactions in real-world systems in the second paragraph of the introduction, but their results are constrained to very small networks, which raises the questions about applicability. The authors only quickly note in the manuscript that this triadic motif can be part of a larger network and that hyperlocking can exist in extended systems and refer to the Supplement, which does not offer a lot of examples. Overall, I agree with the authors about the importance of higher order interactions for real-world systems, but I think it's crucial the authors show and/or discuss how their results can actually be applied or how they contribute to real-world systems. Oscillator networks are important for power-grids, neural systems, and neural networks. How do the results presented here contribute to real-world systems?

> While the analytical theory is developed for a triadic motif to isolate the mechanism, the applicability to larger systems is now explicitly demonstrated on page 7. There, we study random hypernetworks governed by Eq. (9) that combine pairwise and triadic interactions. As shown in Fig. 6, strong triadic coupling induces hyperlocking, while no pairwise phase locking occurs. These results address scalability beyond three-node systems.

(2) One of the main points of the manuscript in my opinion is the fact that the authors found a dynamical behavior that is exclusive for higher-order networks. This is, in fact, highlighted by the

authors in the abstract, introduction, and conclusion. While this is interesting, this is not necessarily new. Higher order interactions have been studied for many years now (https://doi.org/10.1038/s42005-021-00695-0; https://doi.org/10.1016/j.physrep.2023.04.002); and several authors have already shown “exclusive dynamical behavior” in these systems. For example, higher order interactions have been shown to enable chimera states to appear in networks without phase-lag (https://doi.org/10.1103/PhysRevE.105.L042202; https://doi.org/10.1016/j.chaos.2023.113325); or to induce synchronization in parameter regimes in which pairwise interactions wouldn't (https://doi.org/10.1103/PhysRevLett.127.258301); induce solitary states (https://doi.org/10.1103/xtgc-f766); and also to change stability properties in oscillator networks (https://arxiv.org/abs/2510.09387); to cite a few.

We discuss this topic on page 7 in the conclusions.

(3) The current manuscript is written pretty much as a letter. I believe there is a missed opportunity here to expand more on the text and offer the reader more details about the work and its implications. Particularly, the current version is lacking a “discussions” section, in my opinion. The manuscript only offers a short paragraph under “Conclusion”, but no extended discussion about the implications of the results in the literature of higher-order interactions, or even possible applications in real-world systems. Since Nature Communications doesn't have a specific page limit, I believe it would strengthen the manuscript a lot if the authors added more material and discussions to the manuscript.

We expanded the discussion of hyperlocking with additional examples, included experimental details, enlarged the Supplementary Material, and added an outlook section on the effects of hyperlocking in random triadic networks. We did not move technical results from the Supplementary Material into the main text, as these are primarily methodological and would not further clarify the main conceptual message. We also expanded the conclusions and references.

(4) What's the reason behind the choice for Stuart-Landau oscillators? How does this choice match with the empirical setup of chemical oscillators? Are the results also true for other oscillator networks? In the supplement, the authors show similar results for Van-der-Pol oscillators, but I'm curious to what extent this can be observed in different models, e.g. Kuramoto.

The Stuart–Landau oscillator is the normal form of a Hopf bifurcation and is also a natural minimal model for chemical oscillators operating close to the onset of oscillation (Kiss et al, Chaos, 16, 033109, 2006). The effect is not model-specific: in the Supplementary Material, we show analogous behavior for Van-der-Pol oscillators. We now provide results for random triadic networks on page 7 and discuss the Kuramoto model on page 4.

(5) The color-code in the figures is a little bit confusing. For example, in Fig. 1, even though the colors are the same in panels (a), (b), and (c), as far as I understood, they are not related. Blue in Fig. 1 (a) represents the (real part) of the trajectory of node 1, but in (b) it represents the difference in phase between nodes 1 and 2, and in (c) it represents the triplet synchronization that is explored in the paper. I'd recommend the authors to re-think the color scheme. Even though there is nothing necessarily wrong with that, my brain immediately tried to draw connections that are not there.

We changed the colors in Fig. 1 and Fig.4.

(6) What is special about the triplet synchronization (\phi_1 + \phi_2 - 2\phi_3)? While I understand the authors observed this phenomenon, and that this is not observed in networks with pairwise interactions only (for certain coupling choices), I still can't quite grasp what is important about this behavior. For example, why is \phi_1 + \phi_2 - 2\phi_3 more important than \phi_3 + \phi_1 - 2\phi_2? Or even, why is hyperlocking more important than standard pairwise synchronization (both phase locking and phase synchronization)? Phase locking and phase synchronization have strong implications in real-world systems; they have led to important theoretical insights in neuroscience (e.g. Communications through Coherence theory), and also direct applications in power-grid studies. While I enjoyed reading the current manuscript, I think it lacks on this point. This goes back to my comments about having more discussions in the paper, about the results and the implications.

> There is nothing intrinsically special about the specific combination $\phi_1+\phi_2-2\phi_3$; it is selected by the structure of the triadic coupling, and different triadic interactions would select different combinations. The key point is therefore not the particular integers, but the existence of a stable three-phase locking relation that persists while all pairwise phase differences remain unlocked. This distinguishes hyperlocking from pairwise synchronization, where coherence is expressed through pairwise phase relations. We have clarified this distinction and its implications in the Discussion.

(7) The empirical setup is very interesting and the results impressive. At the same time, however, the system has now delayed interactions, so it's not exactly what is described in Eq. (1). It's well-known that delayed interactions can disrupt synchronization in coupled networks, and induce more complicated patterns (e.g. waves, chimeras, clusters). I think there should be more discussion in the manuscript about how this delayed system can be seen as a higher-order network. Also, as a minor point, Fig. 4 could be bigger.

> The experimental implementation indeed involves delays, which were chosen to suppress trivial in-phase pairwise synchrony. The electrochemical oscillators operate close to a Hopf bifurcation, where delayed feedback acts as a phase-shifted (conjugate) coupling. Observations of hyperlocking indicate that the triadic synchronization mechanism is robust to delays, noise, and other experimental imperfections. We expanded the discussion and enlarged Fig. 4.

(8) In Fig. 5 the authors show that different choices for coupling can lead to pairwise locking in their system. I believe this is a quite important point, and it goes back to my previous comments. Why is hyperlocking phenomenon important, and what's the relevance of the type of coupling that leads to this new dynamical phenomenon? The authors only briefly mention Fig. 5 and say that they have results in the supplement about it. But as I mentioned previously, Nature Communications doesn't have a strict limit of 5 pages, so why not add the discussions in the main text? I think this would strengthen the manuscript and give the reader enough information/material to better appreciate the results and their implications.

> Fig. 5 shows that hyperlocking results from an interplay between higher-order topology and nonlinear coupling. Hyperlocking supports coherent collective dynamics that are undetectable at the pairwise level and are instead encoded in collective phase constraints. Following the referee's suggestion, we have expanded the discussion of Fig. 5 and its implications in the main text.

Reviewer #3 (Remarks to the Author):

This manuscript presents a clear and timely study introducing the concept of hyperlocking, a novel synchronization regime emerging from a triadic coupling motif. The authors combine analytical derivations based on phase reduction and normal form theory with numerical simulations and an experimental realization using electrochemical oscillators. The work is technically solid, clearly presented, and likely to be of interest to researchers working on nonlinear dynamics...

We thank the referee for their positive and constructive assessment of the paper.

A few conceptual clarifications would strengthen the manuscript. In the introduction, the authors refer to higher-order interactions in general terms but move directly to a triadic motif, without stating that this is a specific subclass of higher-order interactions. In triadic interactions, one node regulates or modulates the coupling between two others, rather than participating symmetrically in a group interaction.

We added a clarification in the introduction.

In the present model, the coupling term $g(z_3)h(z_2,z_1)$ introduces a hierarchical dependency rather than a symmetric three-body interaction like $h(z_1,z_2,z_3)$. The system captures a specific mechanism of higher-order modulation, but not the full symmetry and mutual dependence of standard higher-order dynamical frameworks. While hyperlocking is intriguing, it would help if the authors discussed whether similar behavior could also emerge in systems with simplicial or symmetric three-body coupling, or if it is specific to this coupling choice. This discussion would help readers appreciate the generality of the effect and its broader relevance to higher-order dynamical systems.

The coupling term represents a triadic, modulatory interaction rather than a fully symmetric three-body interaction. We have clarified this distinction in the Introduction. Whether analogous hyperlocking regimes can also emerge in fully symmetric simplicial systems is an interesting open question

Finally, the electrochemical setup is presented as an experimental validation of the theoretical model, but the triadic coupling is digitally implemented rather than arising intrinsically from the physical system. In this sense, the experiment functions as a controlled hardware realization of the proposed dynamics, rather than a self-organized instance of higher-order coupling. Clarifying this distinction would improve the presentation and prevent possible overinterpretation of the experimental results.

The individual nodes are genuine electrochemical oscillators, subject to experimental heterogeneity, noise, and drift. However, because no quantitative chemical-kinetics model exists for this reaction, the system cannot be regarded as a direct hardware realization of a known set of ordinary differential equations. Instead, the experiment should be understood as a controlled hybrid realization in which real chemical oscillators are coupled through a digitally implemented scheme that mimics a triadic hypernetwork interaction. This approach enables a precise, stable, and reproducible implementation of modulatory coupling while avoiding confounding chemical complexities. We have clarified this distinction in the revised manuscript at the end of the “Hyperlocking with electrochemical oscillator network” and at the beginning of the “Conclusion” sections (pages 6 and 7).

Hypernetworks induce stable hyperlocking

Eddie Nijholt* and Tiago Pereira†
Institute of Mathematical Sciences and Computation, University of São Paulo, São Paulo, Brazil
Department of Mathematics, Imperial College London, London, UK

Matthias Wolfrum‡
Weierstrass Institute for Applied Analysis and Stochastics, Mohrenstr. 39, 10117 Berlin, Germany.

Sagnik Chakraborty§ and István Z. Kiss¶
Department of Chemistry, Saint Louis University, St. Louis, MO 63025, USA.

Jürgen Kurths**
Potsdam Institute for Climate Impact Research - Telegraphenberg A 31, Potsdam, 14473, Germany
(Dated: February 11, 2026)

Hypernetworks capture coupling structures where interactions extend beyond pairs to groups of three or more units, called hyperedges. They are of increasing importance for many systems such as the brain, social groups, ecosystems, and the climate. We describe here a synchronization phenomenon that is distinctive for hypernetworks. We uncover that in a system of three coupled oscillators with resonant frequencies, the coupling by a triadic hypernetwork motif, where a third node modulates the interaction between two others, can induce a stable locking of a phase triplet, while no pairwise locking is observed. Using normal form transformations and phase reduction, we derive analytically how a specific choice of the coupling functions induces this hyperlocking. We confirm our predictions with both numerical simulations and chemical experiments. Our findings uncover a new synchronization regime intrinsic to higher-order interactions and open new directions for controlling real-world complex dynamics beyond pairwise frameworks.

INTRODUCTION

Many natural and engineered systems, from the brain to social groups, ecosystems, and the climate, are composed of interacting elements [1]. A standard modeling assumption for such systems is that interactions happen between pairs of elements [2–5]. While this pairwise modeling has been successful, recent work has shown that, in many applications, interactions between more than two elements cannot be decomposed as a sum of pairwise interactions [6–9]. These interactions between three or more elements are known as higher-order interactions.

Higher-order interactions encompass a broad class of mechanisms, including symmetric interactions, such as simplicial complexes, as well as asymmetric motifs in which one element modulates the interactions between others [6]. Here, we consider the latter case and focus on triadic interactions, where one node regulates or mediates the coupling between two others, rather than participating symmetrically in a group interaction. We depict this situation in Fig. 1.

Such triadic interactions appear in a wide range of systems. For example, in the brain, a triadic interaction appears when communication between two regions depends on the activity of a third one [10]. Similarly, in ecological communities, the interaction between two species can change in the presence of a third [11]. In climate systems, extreme compound events such as simultaneous heatwaves and droughts cannot be explained by pairwise statistics alone [12]. Similarly, the interaction between tipping elements, such as ice sheets and ocean circulation, is often mediated by the state of a third component [13]. In social dynamics, individuals typically require reinforcement from multiple peers to adopt a new behavior, reflecting a dependence on simultaneous rather than cumulative influence [14].

Despite their growing relevance, the dynamical role of higher-order interactions remains elusive. Only recently have mathematical frameworks such as hypergraphs and simplicial complexes provided a direct way to represent and analyse these effects [7, 15, 16]. Parallel advances have shown that higher-order interactions can also be reliably recovered from data [17–19]. These developments reveal how higher-order coupling can reshape collective behaviour [20, 21], alter stability [22], shift the onset of synchronization [23–25], and slow loss of synchrony under parameter changes [26].

In this work, we demonstrate a new synchronization phenomenon that arises in networks with triadic interactions and has no analogue in systems with purely pairwise coupling. We show that, when all oscillators have nearly identical natural frequencies, the triadic motif shown in Fig. 1 admits open sets of coupling functions for which the dynamics converges to a stable triplet-locked state that cannot be decomposed into combinations of pair-

* eddie.nijholt@gmail.com
† tiago@icmc.usp.br
‡ wolfrum@wias-berlin.de
§ sagnik.chakraborty@slu.edu
¶ istvan.kiss@slu.edu
** kurths@pik-potsdam.de

wise phase-locking relations. Remarkably, despite the fact that all oscillator pairs are themselves frequency-resonant, pairwise phase synchronization does not occur. We call this phenomenon hyperlocking: a form of synchronization sustained exclusively by higher-order interactions, for which coherence is achieved only through a genuine triadic phase relation.

We develop a theory, based on phase reduction and normal form transformations, that explains how this behavior emerges. The framework is constructive: it allows us to design coupling functions that realize desired hypernetwork states, thus offering control over collective dynamics. We support our theoretical predictions with both numerical simulations and chemical experiments. Our findings show that triadic interactions can give rise to fundamentally new dynamical regimes, and suggest that they may play a key role in shaping collective behavior in real-world systems.

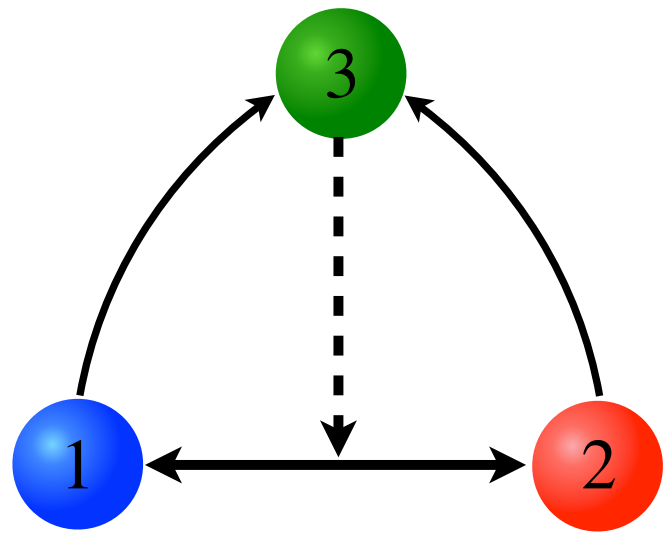

FIG. 1. **Triadic hypernetwork motif.** Pairwise interactions (black arrows) and non-pairwise interaction: node 3 influences the coupling between nodes 1 and 2 (dashed arrow). Together, this establishes a hyperedge including all three nodes and acting on nodes 1 and 2 in a symmetric way.

TRIADIC NETWORK MOTIF

The interaction structure we consider is depicted in Fig. 1. It corresponds to a system of coupled oscillators of the form

$$\begin{aligned} \dot{z}_1 &= f_1(z_1) + \alpha g(z_3) h(z_2, z_1) \qquad (1) \\ \dot{z}_2 &= f_2(z_2) + \alpha g(z_3) h(z_1, z_2) \\ \dot{z}_3 &= f_3(z_3) + \alpha h(z_1, z_3) + \alpha h(z_2, z_3)\,, \end{aligned}$$

with z_j, $j \in \{1, 2, 3\}$, being the dynamical variables for the oscillators and $\alpha \in \mathbb{R}$ the coupling strength. For $\alpha = 0$, i.e., without coupling, each oscillator is assumed to have a stable periodic oscillation with natural frequency ω_j. Eq. (1) captures the higher-order nature of Fig. 1 in the following way. The dyadic coupling function h establishes a classical network structure of pairwise interactions between the nodes, indicated in Fig. 1 by the black arrows. The hypernetwork nature of our motif is induced by the dashed arrow corresponding to the function g. It indicates that node 3 influences the dyadic couplings between nodes 1 and 2, and g, together with h, induces a hyperlink among the three nodes. As we will show below, in a situation close to full resonance, i.e., when each frequency is distinct but is near a common frequency, this type of higher-order coupling can give rise to hyperlocking.

Effective Frequencies and Hyperlocking

Since the oscillators have stable periodic orbits, the oscillatory behavior persists for small couplings $0 < \alpha \ll 1$, however, their frequencies can change because of the interaction and develop an effective frequency. To obtain the effective frequency for each oscillator, we first introduce the phase $\phi_i(t) = \phi_i(z_i(t)) \in S^1 = \mathbb{R}/2\pi\mathbb{Z}$ and the unwrapped phase $\hat{\phi}_i(t) \in \mathbb{R}$. The effective frequency is then given by

$$\Omega_i = \lim_{t\to\infty} \frac{1}{t} \hat{\phi}_i(t).$$

Clearly, it depends on the natural frequencies ω_j, on the network structure, and on the coupling functions.

Pairwise and triplet locking. In networks, one expects to have a phase locking between pairs of connected nodes whenever their natural frequencies are near a resonance $n\omega_i + m\omega_j \approx 0$ for some integers n, m. This means that the effective frequencies exactly satisfy

$$n\Omega_i + m\Omega_j = 0, \qquad (2)$$

which corresponds to the fact that $n\hat{\phi}_i(t) + m\hat{\phi}_j(t)$ stay bounded. Similarly, one can define triplet locking [27], where for three integers (ℓ, n, m) we have

$$\ell\Omega_i + n\Omega_j + m\Omega_k = 0. \qquad (3)$$

Classical theory shows that close to a triplet resonance of the natural frequencies

$$\ell\omega_1 + n\omega_2 + m\omega_3 = \mathcal{O}(\alpha^2)$$

a generic pairwise coupling typically induces a corresponding triplet locking (3). Such a triplet resonance typically comes without any additional pairwise resonances and, hence, no simultaneous pairwise locking is expected. This is completely different in the case of full resonance, where all three frequencies are nearly identical. In this case, all pairs of frequencies are resonant and pairwise locking (2), where one or several of the pairs $(\ell, n), (n, m), (m, \ell)$ are equal to $(1, -1)$, could emerge and, at the same time, non-trivial triplets as, e.g., $(\ell, n, m) = (1, 1, -2)$ are resonant and could induce a corresponding triplet locking (3). Note that at full resonance, triplet locking may also arise as a direct consequence of two pairwise lockings. For a triplet with effective frequencies $(\Omega_1, \Omega_2, \Omega_3)$, if $\Omega_1 = \Omega_2$ and $\Omega_2 = \Omega_3$, then the triplet relation

$$\Omega_1 + \Omega_2 - 2\Omega_3 = 0$$

is satisfied. In fact, two pairwise resonances

$$n\Omega_1 = m\Omega_2 \quad \text{and} \quad r\Omega_2 = s\Omega_3$$

imply not only a third pairwise resonance $nr\Omega_1 = sm\Omega_3$, but also a triplet resonance

$$n\Omega_1 + s\Omega_3 = (m + r)\Omega_2.$$

Here, the triplet relations are *induced* by pairs.

We define *hyperlocking* as a situation where, close to a full resonance, a stable triplet locking arises, while none of the available pairwise resonances induces a corresponding pairwise locking. This means in particular that a triplet locking arising at a triplet resonance we do not designate as hyperlocking, since pairwise locking is excluded *a priori* by the frequency detuning.

Our main result is that, in the hypernetwork motif, robust hyperlocking occurs for proper choices of the coupling function. The synergy between the coupling function and triadic motif generates a collective phase relation that cannot be decomposed into pairwise locking.

Networks versus triadic motif. Notice that if we set $g(z) = 1$, this is equivalent to removing the dashed link from node 3, in Fig. 1. This reduces the triadic motif to a classical network of pairwise coupled oscillators. Then, classical synchronization theory shows that if the natural frequencies are not close to full resonance, but instead satisfy a triplet relation such as $\omega_1 - 2\omega_3 + \omega_2 \approx 0$, then triplet locking may occur [27, 28]. However, near full resonance $\omega_1 \approx \omega_2 \approx \omega_3$, triplet locking appears as a byproduct of pairwise locking generically. Indeed, for networks, the effective dynamics is dominated by pairwise interactions, as we show in Supplementary Note I. This is a fundamental feature of networks: when interactions are pairwise, and nodes are near full resonance, the effective reduced dynamics remain pairwise, generically.

HYPERLOCKING NEAR A HOPF BIFURCATION

We choose Stuart–Landau oscillators

$$f_j(z) = (\lambda + i\omega_j)z - |z|^2 z, \tag{4}$$

where z is a complex number, with $j = 1, 2, 3$, and $\lambda \in \mathbb{R}$ provides the radius of a stable periodic orbit $z_j(t) = \sqrt{\lambda}e^{i\omega_j t}$ with frequency ω_j emerges for positive values of λ. We can achieve the hyperlocking behavior by choosing linear coupling functions

$$\begin{aligned} h(z, w) &= w - \overline{z} \\ g(u) &= u\,. \end{aligned} \tag{5}$$

We choose similar frequencies $\omega_1 = 0.95$, $\omega_2 = 1.05$ and $\omega_3 = 0.98$ together with a small coupling α and solve system (1) numerically using Julia [29]. In Fig. 2(a), we show the time traces of the real parts $\mathrm{Re}(z_j(t))$.

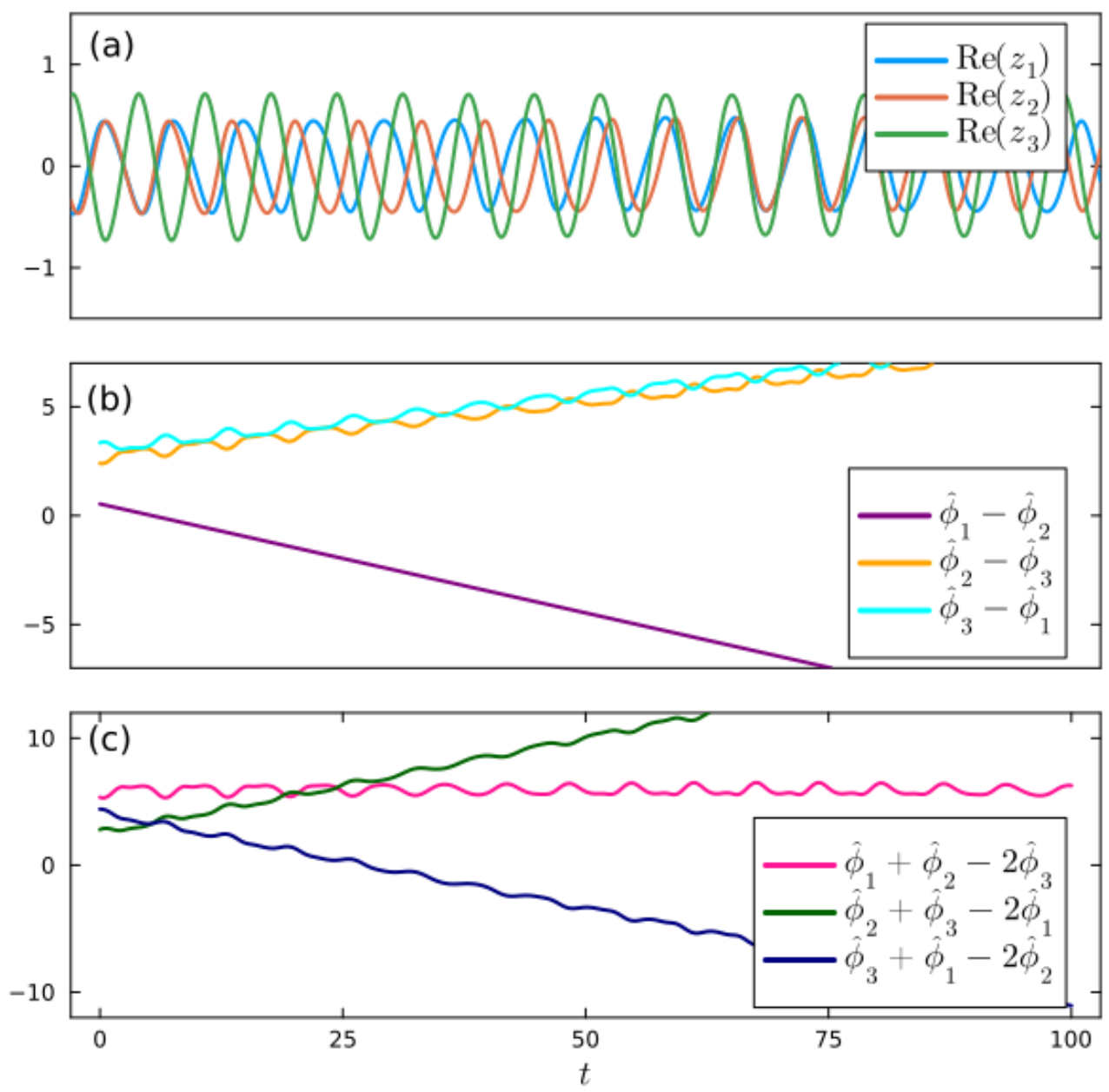

FIG. 2. **Dynamics of the triadic network.** Numerical solution of system (1)–(5). Panel (a) shows the time traces of the real parts of z_1, z_2, and z_3. Panel (b) shows the pairwise phase differences and reveals that no phase locking (where the pairwise phase difference is bounded) is observed. Panel (c) shows the triplet phase differences. The triplet combination $\phi_1 + \phi_2 - 2\phi_3$ (blue) remains bounded, indicating the existence of a hyperlocking between this triplet of phases. Parameters: $\omega_1 = 0.95, \omega_2 = 1.05, \omega_3 = 0.98, \lambda = 0.2, \alpha = 0.15$.

We observe nearly sinusoidal oscillations with amplitude $\sqrt{\lambda} \approx 0.55$. To see the interaction of their phases, we perform a polar decomposition $z_i(t) = r_j e^{i\phi_j}$ and obtain the unwrapped phases $\hat\phi_j$. In Fig. 2(b), we plot the pairwise phase differences between $\hat\phi_i - \hat\phi_j$ for all pairs $i \neq j$ and notice that the differences grow in time, indicating the absence of pairwise frequency locking. However, as seen in Fig. 2(c), there is hyperlocking for $(\ell, m, n) = (1, 1, -2)$, since the corresponding triplet phase $\hat\phi_1 + \hat\phi_2 - 2\hat\phi_3$ stays bounded.

To explore this hyperlocking further, we fix the parameters as before but vary the frequency ω_3. For each value of ω_3, we calculate numerically the effective frequencies Ω_1, Ω_2, and Ω_3 shown in Fig. 3(a). In panel (b), we show the triplet $\Omega_1 + \Omega_2 - 2\Omega_3$. In the vicinity of the triplet resonance of the natural frequencies at $\omega_3 = (\omega_1 + \omega_2)/2$, we observe a large plateau where the triadic coupling induces stable hyperlocking without any pairwise locking. The pairwise locking at the two points of pairwise resonance $\omega_3 = \omega_{1,2}$ is much less pronounced.

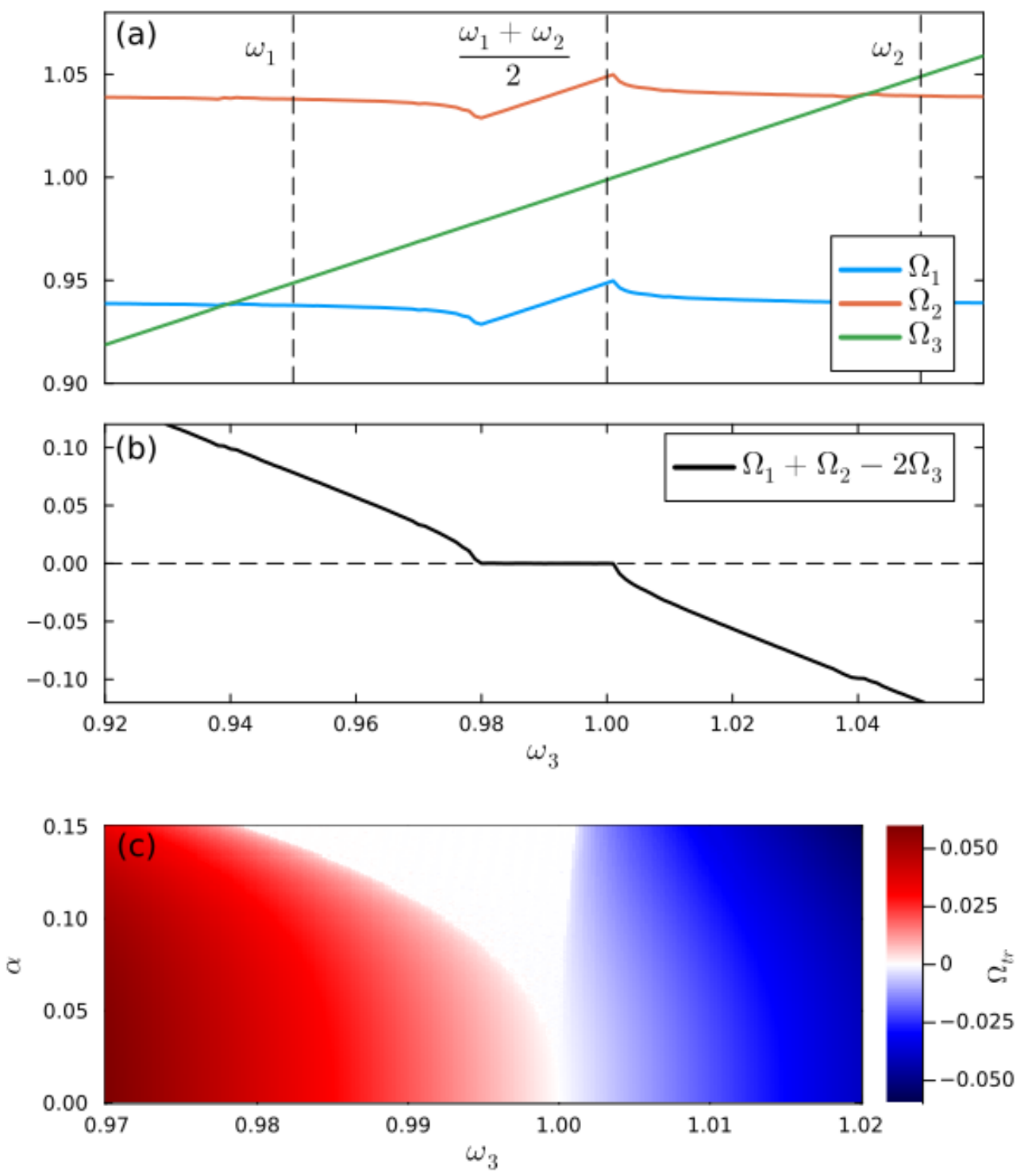

FIG. 3. **Hyperlocking between triplets of phases.** Panel (a) shows effective frequencies for varying natural frequency ω_3. Panel (b) shows the effective frequency triplet $\Omega_{\rm tr} = \Omega_1 + \Omega_2 - 2\Omega_3$ for varying ω_3. The plateau at zero reveals the hyperlocking emergent from the triadic interaction. Panel (c) shows the quadratic locking cone that arises when also the coupling strength α is varied (white region where $\Omega_{\rm tr} = 0$). All other parameters are the same as in Fig. 2.

A THEORY FOR EMERGENT HYPERLOCKING

To explain the hyperlocking behaviour, we develop a normal form theory for the triadic network as shown in Supplementary Note II. We give a short outline of our treatment. For this triadic motif with the specific choice of the coupling functions (5), all coupling terms appearing in first order are *non-resonant*. This means that they represent small, rapid fluctuations that do not reveal the dynamics on larger time scales. To treat the higher-order terms, we employ techniques from normal form theory. The idea is to use successive coordinate transformations on the z-variables to remove as many terms as possible from Eq. (1), before performing the phase reduction. We show that non-resonant terms can be removed.

The coordinate transformations employed in our theoretical analysis are perturbations of the identity map. This proximity ensures that any locking relations observed in the transformed coordinates also manifest in the original system. Therefore, the analytical derivation based on transformed coordinates captures the actual dynamics of the original system.

After the normal form transformations, we obtain the phase equations to leading order

$$\dot{\phi}_1 = \omega_1^{\rm eff} - \alpha_{\rm eff}^2 \sin(2\phi_3 - \phi_1 - \phi_2 + \gamma_1) \qquad (6)$$

$$\dot{\phi}_2 = \omega_2^{\rm eff} - \alpha_{\rm eff}^2 \sin(2\phi_3 - \phi_1 - \phi_2 + \gamma_1) \qquad (7)$$

$$\dot{\phi}_3 = \omega_3 \qquad (8)$$

where $\omega_i^{\rm eff}$ and $\alpha_{\rm eff}$ are the effective frequency and coupling functions. They are obtained in Supplementary Note II. We obtain that $\omega_i^{\rm eff} = \omega_i + O(\alpha^2\lambda)$, and $\alpha_{\rm eff} \approx \alpha\sqrt{\lambda/\omega_3}$. Notice that, to leading order, no pairwise synchronization is present. For instance, consider $\theta = \phi_1 - \phi_2$ and a frequency gap $\omega_1 - \omega_2 = O(\alpha^2)$. In this case, the higher-order corrections to Eqs. (6)-(8) are of order $O(\alpha^3)$, which means that for small coupling, no pairwise locking will be present. Because to obtain a pairwise locking state, the frequency gap needs to be of the same order as the coupling strength. We provide the full argument in Supplementary Note III. On the other hand, we can obtain a stable triplet locking even in the absence of a pairwise locking. Introducing the triplet phase

$$\varphi = \phi_1 + \phi_2 - 2\phi_3,$$

we get the Adler equation

$$\dot{\varphi} = \Delta - 2\alpha_{\rm eff}^2 \sin(\gamma_1 - \varphi)$$

where $\Delta = \omega_1^{\rm eff} + \omega_2^{\rm eff} - 2\omega_3$. In Supplementary Note III, we determine the frequency locking properties of such Adler equations. Hence, our analytical approach explains the numerically observed stable hyperlocking and, in particular, reproduces the plateau observed in Fig. 3(b) and the quadratic locking cone in Fig. 3(c).

Note that the effective phase dynamics in Eqs. (6)–(8) contains only terms of order $O(\alpha^2)$ and no Kuramoto-type pairwise sinusoidal coupling of the form $\sin(\phi_j - \phi_k + \gamma)$. This absence is a direct consequence of the underlying triadic interaction motif. This behavior is fundamentally different from that of the *classical Kuramoto model*, which arises as the phase reduction of Stuart–Landau oscillators with nearly identical natural frequencies and *pairwise linear coupling*. As shown in the Supplementary Note I, for purely pairwise linear coupling, the resonant pairwise terms persist under normal-form transformations and cannot be eliminated by nonlinear coordinate changes. Consequently, when pairs of oscillators are frequency-resonant, pairwise phase locking dominates the dynamics and is generically stable. In this situation, any apparent triplet locking then occurs only as a secondary consequence of pairwise locking.

SYNERGY BETWEEN TRIADIC MOTIF AND NONLINEAR COUPLING

The emergent dynamics of the triadic motif reflect a synergy between the hypernetwork structure and the nonlinearities of the coupling functions. For the class of

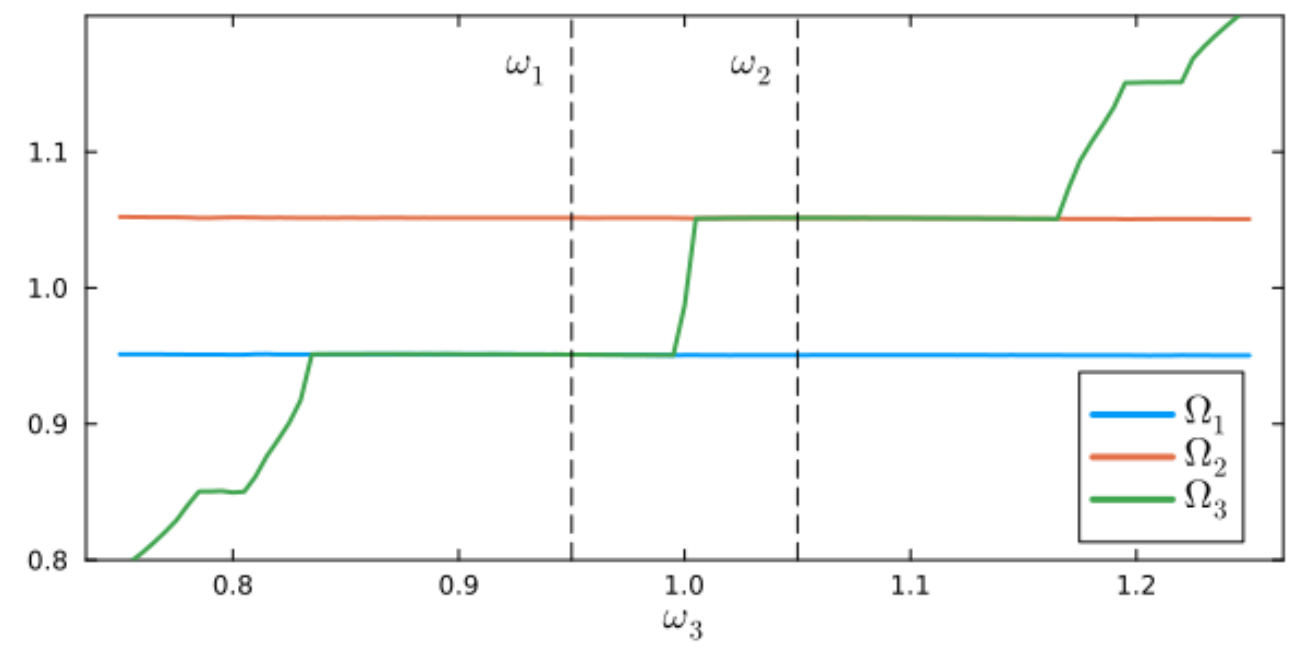

FIG. 4. **Different coupling functions may lead to pairwise locking.** Using in system (1)–(5) the coupling function $h(z, w) = w - z$, pairwise locking becomes predominant. Around the corresponding resonances of the natural frequencies there are two large regions with $\Omega_1 = \Omega_3$ and $\Omega_2 = \Omega_3$. Triplet locking $2\Omega_1 = \Omega_2 + \Omega_3$ and $2\Omega_3 = \Omega_1 + \Omega_3$ occurs only in two small regions close to the corresponding triplet resonances. Parameters as in Fig.2.

coupling functions we consider, it is striking that, despite near full resonance, no pairwise synchronization occurs. A different choice of the coupling function, however, can restore stable pairwise locking: Fig. 4 shows that with the coupling function given as $h(z, w) = w - z$, the dynamics are dominated by pairwise interactions resulting in pairwise locking as can be seen by the plateaus where $\Omega_3 = \Omega_2$ and $\Omega_3 = \Omega_2$, which means that the hyperlocking effect disappears. Note that the coupling still has the same nonlinear triadic structure, only the specific monomials in the interaction are different. But this already results in new resonant terms that are essential for the effective phase dynamics.

HYPERLOCKING WITH ELECTROCHEMICAL OSCILLATOR NETWORK

We confirmed the emergence of triplet locking in the absence of pairwise phase locking through experiments involving three oscillatory electrochemical reactions coupled via nonlinear feedback and delay. The experimental setup, shown in Fig. 5, consists of a multichannel potentiostat connected to the three nickel working electrodes in sulfuric acid electrolyte. At a constant circuit potential with an added external resistance, the electrochemical dissolution of nickel exhibits periodic electrode potential (E_k) oscillations reflecting the variations of the chemical composition of the metal-electrolyte interface.

We consider three such nickel wires that constitute the three nodes of the network. The network coupling is introduced using a real-time controller that implements an external feedback as

$$\delta V_1(t) = K\tilde{E}_3(t)(\tilde{E}_2(t-\tau) - \tilde{E}_1(t))$$
$$\delta V_2(t) = K\tilde{E}_3(t)(\tilde{E}_1(t-\tau) - \tilde{E}_2(t))$$
$$\delta V_3(t) = K((\tilde{E}_1(t-\tau) - \tilde{E}_3(t)) + (\tilde{E}_2(t-\tau) - \tilde{E}_3(t)))$$

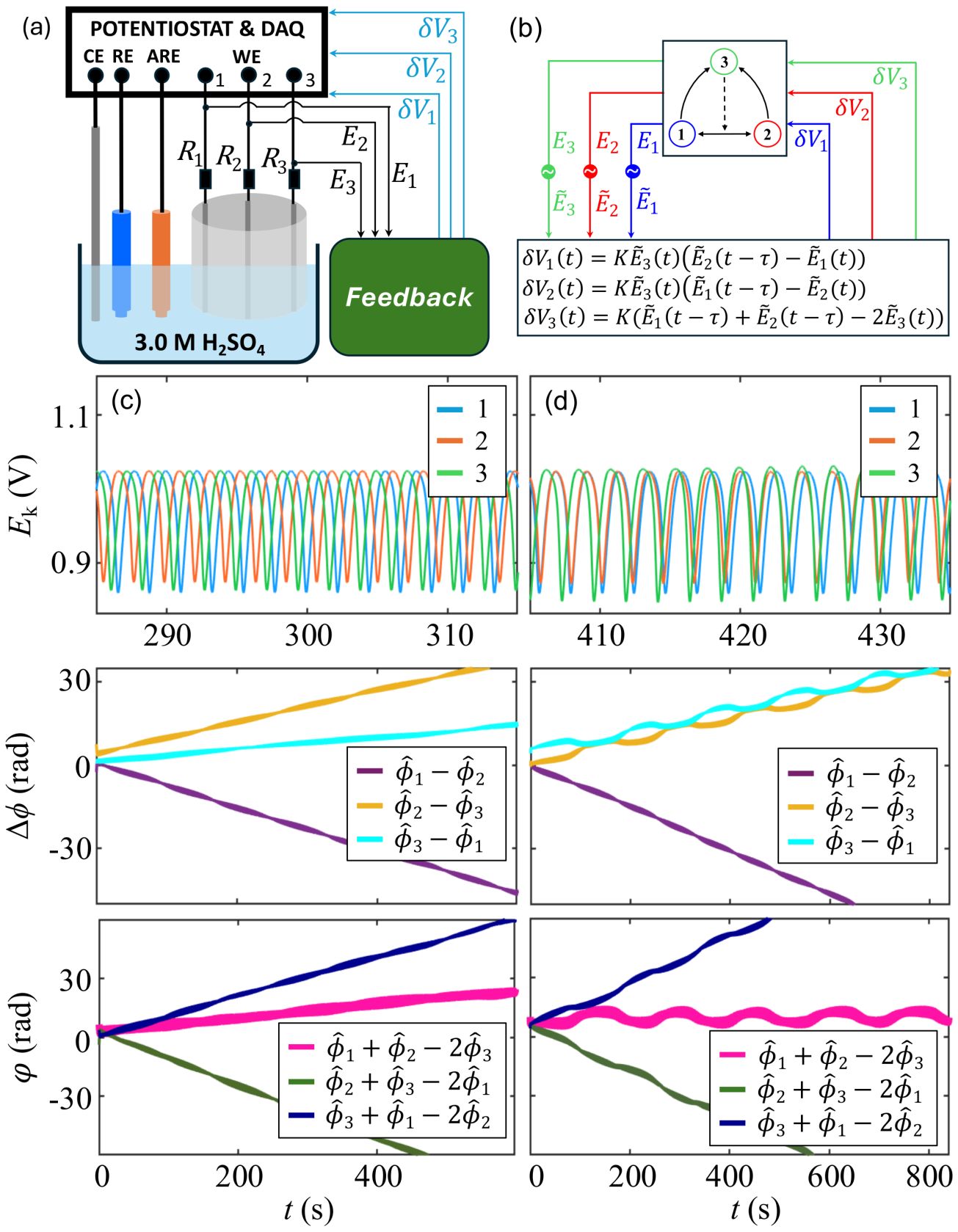

FIG. 5. **Hyperlocking in a triadic hypernetwork motif with electrochemical oscillators.** (a) Experimental setup. CE, RE, ARE, and WE are the counter, reference, auxiliary reference, and the working electrodes. R_k, δV_k, and E_k denote the individual resistors, the circuit potential perturbations, and the measured electrode potentials, respectively. (b) Schematic illustration of the feedback mechanism. K and τ are the feedback strength and delay, and $\tilde{E}_k$ are the rescaled electrode potentials used in the feedback scheme. (c-d) (Top to bottom) Electrode potential time series, evolution of pairwise and triplet phase differences with time, respectively. (c) Uncoupled oscillations. $K = 0$ V^{-1}. (d) Hyperlocking in the presence of feedback. K = -0.2 V^{-1} and τ = 1.1 s.

where δV_k and $\tilde{E}_k(t)$ are the circuit potential perturbation applied via the feedback mechanism and the offset corrected and rescaled electrode potential of the kth electrode, respectively, K is the coupling strength, τ is a time delay. The circuit potentials of oscillators 1 and 2 depend nonlinearly on the states of all three oscillators. Further experimental details are given in Supplementary Note IV. The feedback scheme represents the hypernetwork: Nodes 1 and 2 are delay-coupled, and the coupling strength is modulated by node 3. Node 3 is coupled to nodes 1 and 2 through a delayed difference mechanism.

Note that without a delay term in the coupling, the system would settle into a pairwise, in-phase synchronized state, leading to regular phase locking [30]. In this sense, introducing delay in the experiments serves a role similar to introducing conjugate coupling in the model,

namely, preventing stable pairwise synchronization. We adopted this form of coupling because delays are known to disrupt synchronization in coupled networks [31]. The delayed nonlinear feedback applied here does not produce obvious synchronization patterns, for example, one or multiple phase-cluster states [32] due to the modulation of the coupling strength.

To describe the nature of the phase dynamics, the phases $\hat{\phi}_k$, from the electrode potential oscillations, were extracted using the Hilbert transform technique [33]. The natural frequency of each oscillator was adjusted with a set of resistors so that ω_2 - ω_1 = 12 mHz, ω_3 - ω_1 = 3 mHz and ω_2 - ω_3 = 9 mHz. The natural frequencies $\omega_1 \approx 448$ mHz, $\omega_2 \approx 460$ mHz, and $\omega_3 \approx 451$ mHz create a condition for triplet resonance, as there is a small detuning for ω_1 - $2\omega_3$ + ω_2 = 6 mHz. Fig. 5 (c) demonstrates that without coupling ($K = 0$ V^{-1}), the electrode potentials of the three nodes oscillate independently and the pairwise and triplet phase differences (including φ) vary linearly with time. In this state, there is no pairwise or triplet phase locking.

Fig. 5 (d) shows the results in the presence of feedback with K = -0.2 V^{-1} and τ = 1.1 s (half the mean period of oscillations of the three nodes). No pairwise synchrony was observed: $\hat{\phi}_2 - \hat{\phi}_3$ and $\hat{\phi}_3 - \hat{\phi}_1$ show oscillations and drift with time. The triplet phase difference φ exhibited phase locking (hyperlocking, with 0 mHz triplet frequency difference) with a small amplitude oscillation, while the other two possible triplet phase differences drifted with time. The experiments demonstrate that hyperlocking is a robust phenomenon in a hypernetwork motif where pairwise locking is not possible due to the nature of the nonlinear interactions.

The electrochemical dynamics are chemically complex, involving multiple reaction pathways such as direct metal oxidation, the formation of oxide films, oxygen evolution, and the adsorption of chemical species within a diffuse double layer [34]. The experimental observation of hyperlocking, therefore, indicates that the phenomenon is robust to intrinsic nonlinearities, measurement noise, and slow experimental drifts. Although coupling was implemented using an external feedback loop, this setup effectively mimics realistic situations in which interaction strengths depend nonlinearly on the system state.

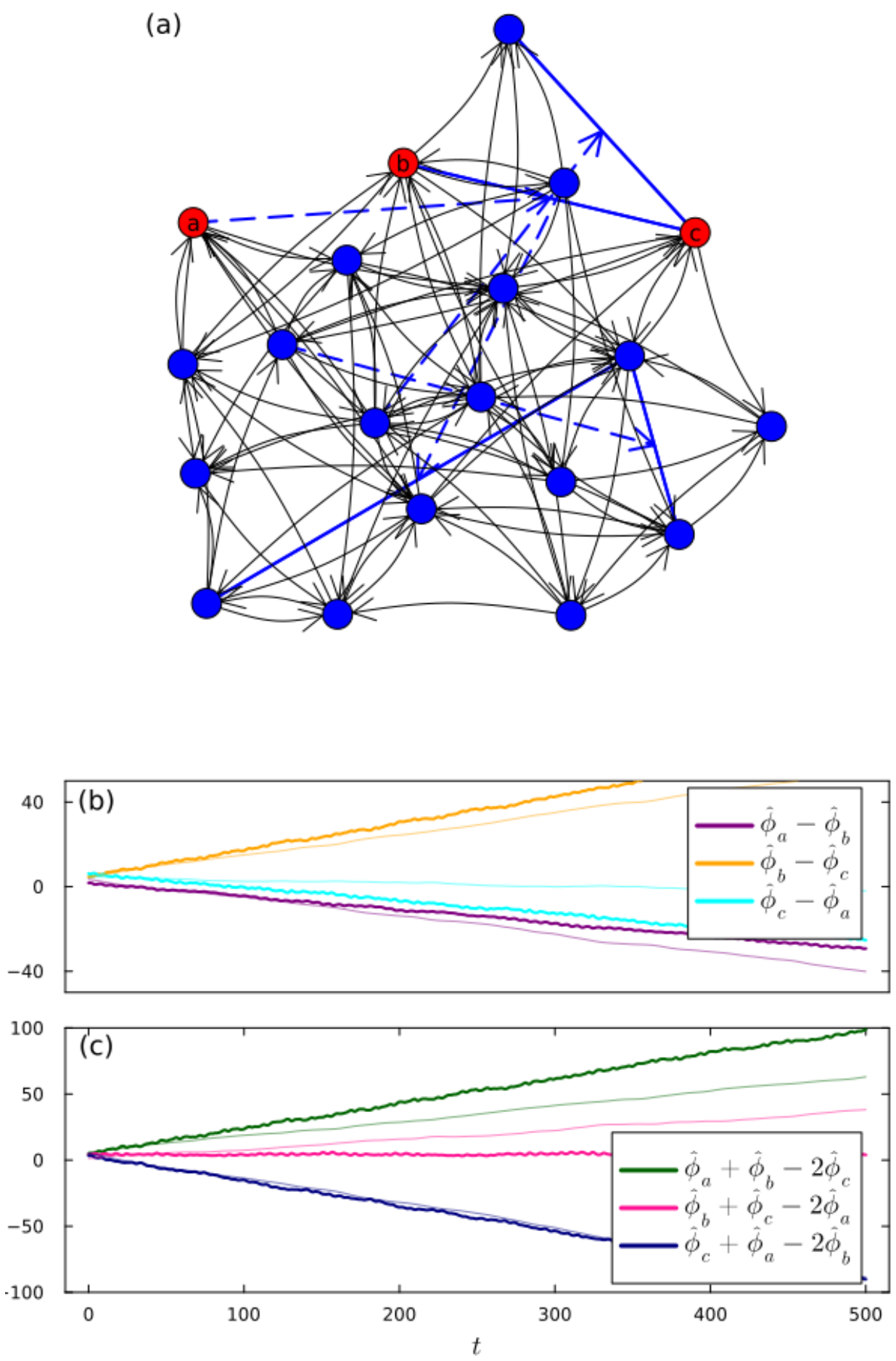

FIG. 6. **Hyperlocking in a larger network containing triadic motifs.** (a) Random hypernetwork with directed pairwise links (black arrows) and triadic coupling (blue), where a third node influences (dashed blue arrow) the interaction between a pair (blue arrows). The triplet of red nodes has frequencies $\omega_{a,b,c}$ close to full resonance. All other natural frequencies are chosen randomly. The dynamics of system (9) show a hyperlocking of this triplet for sufficiently strong triplet coupling strength α (dark colors), while for $\alpha = 0$, i.e., only pairwise but no triplet coupling, no hyperlocking is present (light colors). (b) Pairwise frequency differences of the resonant triplet show no pairwise locking among these nodes. (c) Triplet phase differences show hyperlocking when triplet coupling is present. Parameters: $\alpha = 1.5$, $\beta = 1.0$, $\lambda = 0.3$.

OUTLOOK: HYPERLOCKING FOR OTHER NONLINEAR OSCILLATORS AND LARGER NETWORKS

So far, we have investigated hyperlocking in a triadic hypernetwork motif of three coupled oscillators near a Hopf bifurcation. In Supplementary Note V, we demonstrate numerically that a similar coupling structure can also induce hyperlocking in a system of three nonlinear van der Pol oscillators.

The hyperlocking mechanism uncovered here is not restricted to the specific triadic motif. Rather, this motif can serve as a fundamental building block for more complex networks that contain such triadic structures and exhibit hyperlocking behavior. In the Supplementary Note III, we demonstrate how our analytical theory can be applied similarly to an extended motif containing the triadic motif. Below, we give an example of a hypernetwork consisting of a set $\mathcal{N} = \{1, \ldots, n\}$ of oscillating nodes with randomly chosen directed pairwise links $j \to k$, given by

a directed graph $\mathcal{P}$, and an additional randomly selected collection $\mathcal{T}$ of triplet hyperlinks $k \to \{j, \ell\}$, where node k influences a pairwise interaction between nodes j and ℓ. For $\mathcal{T}$, we admit only triplets of nodes with no pairwise links in $\mathcal{P}$. The dynamics are given by

$$\dot{z}_j = f_j(z_j) + \frac{\beta}{|\mathcal{P}|} \sum_{(j\to k)\in\mathcal{P}} q(z_k, z_j) + \frac{\alpha}{|\mathcal{T}|} \sum_{(k\to\{j,\ell\})\in\mathcal{T}} g(z_k) h(z_\ell, z_j) \quad (9)$$

with Stuart–Landau oscillators f_j as in (4) and triplet coupling functions g and h from (5) with coupling strength α. For the pairwise coupling, we choose $q(z, w) = z - w$ with coupling strength β.

For the numerical simulations shown in Fig. 6, we have first generated a random hypernetwork with $n = 20$ nodes, 93 directed links, and four triplet hyperedges, see panel (a). After choosing random natural frequencies equally distributed in the interval $[0.7, 1.3]$, we have slightly adapted the frequencies $\omega_{a,b,c}$ of the nodes in one of the hyperlinks to achieve a situation sufficiently close to a full resonance among these nodes. In panels (b) and (c), we plotted the pairwise and triplet phase combinations among these nodes with (dark colors) and without (light colors) the triplet coupling. The results clearly show that sufficiently strong triplet coupling induces hyperlocking among these nodes.

These cases illustrate that hyperlocking can emerge in extended systems through the embedding of triadic interactions, highlighting the broader relevance of this phenomenon for the dynamics of higher-order networks.

CONCLUSION

We have shown that hyperlocking emerges as a synergy between a triadic coupling structure and a nonlinear coupling function. We demonstrate that hyperlocking is a robust dynamical phenomenon that appears in a triadic motif of coupled Stuart-Landau oscillators, where it can be treated analytically. The experiments on electrochemical oscillators demonstrated that hyperlocking is robust under realistic conditions, including experimental noise and slow parameter drifts caused by electrode deterioration. Although the oscillations originate from a Hopf bifurcation [35], the observed waveforms indicate that the system operates far from the bifurcation point, as illustrated in Fig. 5, showing that hyperlocking is not a near-bifurcation effect. Consistently, we also observe hyperlocking in other dynamical settings, including networks of coupled van der Pol oscillators.

There is extensive literature showing that higher-order interactions can modify known dynamical phenomena in oscillator networks, such as chimeras [36], synchronization thresholds [37], and solitary states [38], often by shifting parameter regimes or stability boundaries relative to purely pairwise coupling. Our result is qualitatively different. In the present system, when the coupling reduces to a purely pairwise network, no triplet locking appears. The hyperlocking reported here arises only from triadic interactions, rather than as a higher-order correction to effects of pairwise coupling. Our results also highlight that the boundary between network and hypernetwork dynamics is not clear-cut. Only the interplay between network structure, coupling functions, and resonances decides the true nature of the emergent dynamical interactions in the system.

Our analytical approach to this hyperlocking consists of performing a nonlinear change of coordinates that preserves dynamical phenomena. In these new coordinates, pairwise resonant terms are eliminated, while triadic interactions survive and organize the dynamics. Therefore, hyperlocking cannot be reduced to a sum of pairwise interactions. In certain scenarios, a higher-order interaction can be expressed as a combination of pairwise interactions, see for instance [39]. In this case, the same vector field can be represented in different bases. An instructive example is when the coupling function is linear, and the higher-order interaction can be expressed as a sum of pairwise interactions [40]. In this sense, higher-order or pairwise terms sometimes provide just alternative representations of the same system.

We introduce a nonlinear change of variables that maps the system to a dynamically equivalent vector field, retaining higher-order terms only when they are dynamically relevant. This coordinate transformation changes the vector field and allows us to demonstrate that hyperlocking is a dynamical effect induced by higher-order interactions, rather than a reparametrization of pairwise coupling. Triadic motifs emerge as flexible building blocks of collective behavior, capable of supporting synchronization regimes that cannot be sustained by networks with pairwise interactions alone, and point to a broader class of higher-order dynamical phenomena awaiting exploration.

Hyperlocking is a general synchronization mechanism in networks with higher-order interactions, in which certain expected frequency lockings, although formally possible, are not dynamically selected, and coherence emerges only through higher-order relations. Surprisingly, we show that hyperlocking is negligible in generic networks with purely pairwise coupling. Triadic networks, therefore, constitute minimal building blocks in which this phenomenon can occur. These findings demonstrate that, in networks with higher-order interactions, synchronization cannot in general be reduced to the analysis of pairwise correlations and is intrinsically multibody in nature. This opens several directions for future research, including extending the analysis to other bifurcation scenarios that generate periodic oscillations in the isolated system, examining whether similar effects arise in systems with symmetric multibody interactions, and exploring the possibility of hyperlocking between clusters of oscillators rather than between individual units.

Acknowledgements. We thank Bob Rink, Chris Bick, Dima Turaev, and Deniz Eroglu for enlightening discussions. TP was supported in part by FAPESP Grant No. 2023/13706-0 and CNPq 312287/2021-6. EN was supported by FAPESP Grant No. 2024/00930-2. TP and EN were partially supported by Serrapilheira Institute (Grant No. 270 Serra-1709-16124) and warmly thank the hospitality of the Weierstrass Institute during their multiple visits. IZK and SC acknowledge financial support from the National Science Foundation Grant No 1900011 and NSF-NIGMS 1R01GM157609 grant. MW acknowledges the hospitality during his research stay at ICMC-USP. TP thanks the Humboldt Foundation for support through a Bessel Fellowship.

Data Availability. The data supporting the findings of this study consist of (i) numerical simulation outputs and (ii) experimental time series from electrochemical oscillator networks. Processed datasets underlying the figures and main analyses, as well as the raw experimental time series, are available from the corresponding author upon reasonable request. All data necessary to reproduce the main results are included in the paper and its Supplementary Information.

Code Availability. The numerical simulations and analysis routines used in this study were implemented in custom Python/MATLAB/Julia scripts. The code required to reproduce the main numerical results and figures is available upon reasonable request.

[1] T. Stankovski, T. Pereira, P. V. McClintock, and A. Stefanovska, Coupling functions: universal insights into dynamical interaction mechanisms, Reviews of Modern Physics **89**, 045001 (2017).
[2] M. Golubitsky, I. Stewart, and A. Török, Patterns of synchrony in coupled cell networks with multiple arrows, SIAM Journal on Applied Dynamical Systems **4**, 78–100 (2005).
[3] T. Pereira, D. Eroglu, G. B. Bagci, U. Tirnakli, and H. J. Jensen, Connectivity-driven coherence in complex networks, Phys. Rev. Lett. **110**, 234103 (2013).
[4] O. E. Omel'chenko and M. Wolfrum, Nonuniversal transitions to synchrony in the Sakaguchi-Kuramoto model, Phys. Rev. Lett. **109**, 164101 (2012).
[5] E. Nijholt, T. Pereira, F. C. Queiroz, and D. Turaev, Chaotic behavior in diffusively coupled systems, Communications in Mathematical Physics **401**, 2715 (2023).
[6] G. Bianconi, *Higher-order networks* (Cambridge University Press, 2021).
[7] C. Bick, E. Gross, H. A. Harrington, and M. T. Schaub, What are higher-order networks?, SIAM review **65**, 686 (2023).
[8] A. P. Millán, H. Sun, L. Giambagli, R. Muolo, T. Carletti, J. J. Torres, F. Radicchi, J. Kurths, and G. Bianconi, Topology shapes dynamics of higher-order networks, Nature Physics **21**, 353–361 (2025).
[9] R. Tönjes, C. E. Fiore, and T. Pereira, Coherence resonance in influencer networks, Nature Communications **12**, 72 (2021).
[10] S. Rosenthal, J. M. Shine, and M. Breakspear, Triadic interactions and functional information dynamics in brain networks, Nature Communications **14**, 1469 (2023).
[11] J. Grilli, G. Barabás, M. J. Michalska-Smith, and S. Allesina, Higher-order interactions stabilize dynamics in competitive network models, Nature **548**, 210 (2017).
[12] J. Zscheischler, S. Westra, B. Van Den Hurk, S. I. Seneviratne, P. J. Ward, A. Pitman, A. AghaKouchak, D. N. Bresch, M. Leonard, T. Wahl, and X. Zhang, Future climate risk from compound events, Nature Reviews Earth & Environment **1**, 333 (2020).
[13] T. Liu, N. Boers, R. Winkelmann, J. Kurths, B. Goswami, N. Wunderling, and J. F. Donges, Network interaction among tipping elements in the earth system, Nature Climate Change **13**, 67 (2023).
[14] I. Iacopini, G. Petri, A. Barrat, and V. Latora, Simplicial models of social contagion, Nature Communications **10**, 2485 (2019).
[15] F. Battiston, G. Cencetti, I. Iacopini, V. Latora, M. Lucas, A. Patania, J.-G. Young, and G. Petri, The physics of higher-order interactions in complex systems, Nature Physics **17**, 1093 (2021).
[16] D. Liuzza, F. Della Rossa, F. L. Iudice, and P. De Lellis, Nonlinear average consensus over circulating directed hypergraphs, IEEE Control Systems Letters (2025).
[17] A. Santoro, F. Battiston, G. Petri, and E. Amico, Higher-order organization of multivariate time series, Nature Physics **19**, 221–229 (2023).
[18] F. Malizia, A. Corso, L. V. Gambuzza, G. Russo, V. Latora, and M. Frasca, Reconstructing higher-order interactions in coupled dynamical systems, Nature Communications **15**, 5184 (2024).
[19] E. Nijholt, J. L. Ocampo-Espindola, D. Eroglu, I. Z. Kiss, and T. Pereira, Emergent hypernetworks in weakly coupled oscillators, Nature communications **13**, 4849 (2022).
[20] G. Ferraz de Arruda, A. Aleta, and Y. Moreno, Contagion dynamics on higher-order networks, Nature Reviews Physics **6**, 468 (2024).
[21] Y. Zhang, M. Lucas, and F. Battiston, Higher-order interactions shape collective dynamics differently in hypergraphs and simplicial complexes, Nature Communications **14**, 1605 (2023).
[22] Y. Zhang, P. S. Skardal, F. Battiston, G. Petri, and M. Lucas, Deeper but smaller: Higher-order interactions increase linear stability but shrink basins, Science Advances **10**, eado8049 (2024).
[23] S. Rakshit, B. K. Bera, E. M. Bollt, and D. Ghosh, Intralayer synchronization in evolving multiplex hypernetworks: Analytical approach, SIAM Journal on Applied Dynamical Systems **19**, 918 (2020).
[24] P. S. Skardal and A. Arenas, Higher order interactions in complex networks of phase oscillators promote abrupt synchronization switching, Communications Physics **3**, 218 (2020).
[25] A. Civilini, O. Sadekar, F. Battiston, J. Gómez-Gardeñes, and V. Latora, Explosive cooperation in social dilemmas on higher-order networks, Phys. Rev. Lett. **132**, 167401 (2024).

[26] S. von der Gracht, E. Nijholt, and B. Rink, Higher-order interactions lead to 'reluctant' synchrony breaking, Proceedings of the Royal Society A: **480**, 20230945 (2024).

[27] B. Kralemann, A. Pikovsky, and M. Rosenblum, Detecting triplet locking by triplet synchronization indices, Phys. Rev. E **87**, 052904 (2013).

[28] J. Jia, Z. Song, W. Liu, J. Kurths, and J. Xiao, Experimental study of the triplet synchronization of coupled nonidentical mechanical metronomes, Scientific Reports **5**, 17008 (2015).

[29] C. Rackauckas and Q. Nie, Differentialequations.jl – a performant and feature-rich ecosystem for solving differential equations in julia, Journal of Open Research Software 10.5334/jors.151 (2017).

[30] J. L. Ocampo-Espindola, I. Z. Kiss, C. Bick, and K. C. A. Wedgwood, Strong coupling yields abrupt synchronization transitions in coupled oscillators, Phys. Rev. Res. **6**, 033328 (2024).

[31] Y. Zhai, I. Z. Kiss, and J. L. Hudson, Control of complex dynamics with time-delayed feedback in populations of chemical oscillators: Desynchronization and clustering, Ind. Eng. Chem. Res. **47**, 3502 (2008).

[32] C. Bick, M. Sebek, and I. Z. Kiss, Robust weak chimeras in oscillator networks with delayed linear and quadratic interactions, Phys. Rev. Lett. **119**, 168301 (2017).

[33] A. Pikovsky, M. Rosenblum, and J. Kurths, *Synchronization: A Universal Concept in Nonlinear Sciences* (Cambridge University Press, 2001).

[34] D. Haim, O. Lev, L. M. Pismen, and M. Sheintuch, Modeling periodic and chaotic dynamics in anodic nickel dissolution, J. Phys. Chem. **96**, 2676 (1992).

[35] I. Z. Kiss, Z. Kazsu, and V. Gáspár, Tracking unstable steady states and periodic orbits of oscillatory and chaotic electrochemical systems using delayed feedback control., Chaos **16**, 033109 (2006).

[36] S. Kundu and D. Ghosh, Higher-order interactions promote chimera states, Physical Review E **105**, L042202 (2022).

[37] Y. Zhang, V. Latora, and A. E. Motter, Unified treatment of synchronization patterns in generalized networks with higher-order, multilayer, and temporal interactions, Communications Physics **4**, 195 (2021).

[38] V. V. Semenov, S. Dutta, S. Boccaletti, C. I. Del Genio, S. Jalan, and A. Zakharova, Solitary states in spiking oscillators with higher-order interactions, Physical Review E **112**, 034302 (2025).

[39] L. Neuhäuser, M. Scholkemper, F. Tudisco, and M. T. Schaub, Learning the effective order of a hypergraph dynamical system, Science Advances **10**, eadh4053 (2024).

[40] R. Lambiotte, M. Rosvall, and I. Scholtes, From networks to optimal higher-order models of complex systems, Nature Physics **15**, 313–320 (2019).

Reviewer #1 (Remarks to the Author):

I thank the authors for the revisions which improve the manuscript. In particular, I think the addition of the larger random networks adds a lot. I still have a few minor concerns detailed below: "triplet locking is reported near triplet resonances of the form ω1 + ω2 - 2 ω3 = 0, where no pairwise resonances are present (...) hyperlocking occurs near a full resonance ω1 ≈ ω2 ≈ ω3," Thank you, this clarifies it. I would however be careful with how hyperlocking is phrased in the manuscript. The abstract reads "Our findings uncover a new synchronization regime intrinsic to higher-order interactions" but from what I understand, the triplet locking ([28]) and your hyperlocking are the same "synchronization regime" in the sense that, from experimental time series, if one doesn't know the natural frequencies of the oscillators, there is no way of distinguishing the two dynamics: in both cases, triplet locking is present but pairwise locking is not. So it is not really a new regime. The two cases are then different "mechanisms" to obtain the same "stable regime". In fact, the conclusion uses this phrasing: "Hyperlocking is a general synchronization mechanism in networks with higher-order interactions".

> We thank the referee. We now adopt the word mechanism instead of regime. As for the distinction of hyperlocking, we notice that under the assumption of small coupling α the frequency shifts are also of order α. Thus, we can discern from the time series the frequencies and discern whether the network is near full resonance.

"The coupling functions shown in Eq. (4) and Fig. 5 were not chosen ad hoc. They are derived from our normal form approach, so we already reverse-engineered them." I thank the authors for the explanation. However, I don't see this mentioned in the manuscript? This needs to be mentioned, problably already near Eq 4.

> We added to the manuscript after Eq. 5 in pg 3.

"The hyperlocked state is hyperbolic, as shown in Supp. Mat. III. As a consequence, it is structurally stable under small smooth perturbations." Similarly, I thank the authors for the explanation but don't find it in the manuscript or the SM. Please mention this somewhere in the main text, it is an important but technical fact that not enough readers of the journal will have knowledge of.

> We added to the manuscript in pg. 5.

"We have expanded the text to include more simulations, examples, and further explanations, including hyperlocking in random triadic networks (pages 6 and 7)." This is very insightful, thank you. This new text also drew my attention to the fact that the motif considered contains *directed* interactions. I think this does not diminish the results, but it should be made more explicit in the abstract/intro/conclusion because most of the literature on synchronization on higher-order networks considers undirected interactions, and the results of the present manuscript shows a nice effect of directed interactions which, a priori, wouldn't have any analogous effect for undirected interactions. Based on this, it might also be worth briefly mentioning studies on the sync with higher order directed interactions and what we know from them. For example: Gallo, L., Muolo, R., Gambuzza, L. V., Latora, V., Frasca, M., & Carletti, T. (2022). Synchronization induced by directed higher-order interactions. Comm, Phys, 5, 263.

> Thank you. We have included this citation (now Ref [10]) in the introduction.

Reviewer #2 (Remarks to the Author):

I appreciate the authors' efforts in revising the manuscript. In my view, the new version provides a more balanced presentation of the work, particularly in how it frames the broader context, engages with prior studies on hypernetwork dynamics, and clarifies the novelty of the current contribution. I also found the new discussion section helpful and engaging. Overall, I believe the manuscript has improved substantially compared to the original submission. I have one remaining point that I would appreciate further clarification on. Based on the authors' response and on the revised manuscript, time delays are necessary for the emergence of hyperlocking in the experimental setup. Without delays, the network would exhibit "trivial in-phase pairwise synchrony". As I understand it, the authors argue that the delayed interactions effectively mimic the triadic motif coupling. I might be missing something here, but it seems plausible that different forms of delayed interactions could also generate an effective triadic motif (perhaps with different parameter regimes), yet not necessarily lead to hyperlocking. Is that correct? If so, how does this align with the framework presented in the manuscript and with the claim that the triadic motif is necessary for hyperlocking? More specifically, I would appreciate clarification on two related points: (1) Is it possible to obtain hyperlocking in a pairwise network with appropriately structured delays (for example, distance-dependent delays)? (2) I am not fully convinced that the empirical results demonstrate robustness to delays. In the current setup, delays appear to be the key mechanism that enables the triadic motif coupling; without them, hyperlocking does not occur. To be considered "robust" I'd expect the behavior to exist in spite of a given factor (delays in this case), not because of it.

> We would like to clarify that the triadic interaction is a structural feature of the coupling implemented in the experiments: the coupling between oscillators 1 and 2 is modulated by oscillator 3. This realizes the hypernetwork motif considered in the theory. The delay does not generate this motif.
>
> The role of the delay is different. In the weak-coupling, near-Hopf regime, the normal form reduction shows that the physical coupling appears as a complex coupling strength, whose phase can be tuned by small delays (see Choe et al. PRE 2010). Delays are used to adjust the effective interaction phase and not to induce higher-order structure. We added a sentence to state why the physical coupling form mimics the motif proposed in the theory, and why the delay was necessary in the experiment. We added this to the manuscript in pg. 6.

it seems plausible that different forms of delayed interactions could also generate an effective triadic motif (perhaps > with different parameter regimes), yet not necessarily lead to hyperlocking. Is that correct? Different forms of coupling motifs with different

amount of delays can generate different types of effective triadic motifs in the phase model, and only certain types of phase models can generate hyperlocking with given resonances. While this statement is true, it is also a vague, generic statement without much practical use. Instead, the manuscript shows that oscillators close to Hopf bifurcation (a common form oscillatory behavior), given a very general coupling hypernetwork motif (oscillator 3 modulating the coupling between oscillators 1 and 2) , with coupling mitigated by conjugate variables (can be adjusted by delay), yield hyperlocking. There could be other types of motifs, coupling forms, and oscillators that can produce hyperlocking - this is an interesting future direction.

(1) Is it possible to obtain hyperlocking in a pairwise network with appropriately structured delays (for example, distance-dependent delays)?

> Within the theoretical framework considered here (networks of coupled Stuart–Landau oscillators), delays alone cannot generate hyperlocking from purely pairwise interactions. They only modify the phase of pairwise coupling. In particular, as shown in Supplementary Material Note 1, pairwise networks on regular graphs, whether with standard or conjugate coupling, do not exhibit hyperlocking. In this sense, the triadic motif is a necessary structural ingredient in our framework.

(2) I am not fully convinced that the empirical results demonstrate robustness to delays.

> We have clarified this point and added several new experimental results in the Supplementary Material. Specifically, we performed additional experiments with delays varied by 2-4% relative to the values used in the main text. We clearly find that hyperlocking persists across these variations. We therefore use "robustness to delays" in the sense that the phenomenon persists over a range of delay values, rather than occurring at a finely tuned parameter.

Hypernetworks induce stable hyperlocking

Eddie Nijholt* and Tiago Pereira†
Institute of Mathematical Sciences and Computation, University of São Paulo, São Paulo, Brazil
Department of Mathematics, Imperial College London, London, UK

Matthias Wolfrum‡
Weierstrass Institute for Applied Analysis and Stochastics, Mohrenstr. 39, 10117 Berlin, Germany.

Sagnik Chakraborty§ and István Z. Kiss¶
Department of Chemistry, Saint Louis University, St. Louis, MO 63025, USA.

Jürgen Kurths**
Potsdam Institute for Climate Impact Research - Telegraphenberg A 31, Potsdam, 14473, Germany
(Dated: May 5, 2026)

Hypernetworks capture coupling structures where interactions extend beyond pairs to groups of three or more units, called hyperedges. They are of increasing importance for many systems such as the brain, social groups, ecosystems, and the climate. We describe here a synchronization phenomenon that is distinctive for hypernetworks. We uncover that in a system of three coupled oscillators with resonant frequencies, the coupling by a triadic hypernetwork motif, where a third node modulates the interaction between two others, can induce a stable locking of a phase triplet, while no pairwise locking is observed. Using normal form transformations and phase reduction, we derive analytically how a specific choice of the coupling functions induces this hyperlocking. We confirm our predictions with both numerical simulations and chemical experiments. Our findings uncover a new synchronization ~~regime~~ mechanism intrinsic to higher-order interactions and open new directions for controlling real-world complex dynamics beyond pairwise frameworks.

INTRODUCTION

Many natural and engineered systems, from the brain to social groups, ecosystems, and the climate, are composed of interacting elements [1]. A standard modeling assumption for such systems is that interactions happen between pairs of elements [2–5]. While this pairwise modeling has been successful, recent work has shown that, in many applications, interactions between more than two elements cannot be decomposed as a sum of pairwise interactions [6–9]. These interactions between three or more elements are known as higher-order interactions.

Higher-order interactions encompass a broad class of mechanisms, including symmetric interactions, such as simplicial complexes, as well as asymmetric motifs in which one element modulates the interactions between others ~~[6]~~[6, 10]. Here, we consider the latter case and focus on triadic interactions, where one node regulates or mediates the coupling between two others, rather than participating symmetrically in a group interaction. We depict this situation in Fig. 1.

Such triadic interactions appear in a wide range of systems. For example, in the brain, a triadic interaction appears when communication between two regions depends on the activity of a third one [11]. Similarly, in ecological communities, the interaction between two species can change in the presence of a third [12]. In climate systems, extreme compound events such as simultaneous heatwaves and droughts cannot be explained by pairwise statistics alone [13]. Similarly, the interaction between tipping elements, such as ice sheets and ocean circulation, is often mediated by the state of a third component [14]. In social dynamics, individuals typically require reinforcement from multiple peers to adopt a new behavior, reflecting a dependence on simultaneous rather than cumulative influence [15].

Despite their growing relevance, the dynamical role of higher-order interactions remains elusive. Only recently have mathematical frameworks such as hypergraphs and simplicial complexes provided a direct way to represent and analyse these effects [7, 16, 17]. Parallel advances have shown that higher-order interactions can also be reliably recovered from data [18–20]. These developments reveal how higher-order coupling can reshape collective behaviour [21, 22], alter stability [23], shift the onset of synchronization [24–26], and slow loss of synchrony under parameter changes [27].

In this work, we demonstrate a new synchronization phenomenon that arises in networks with triadic interactions and has no analogue in systems with purely pairwise coupling. We show that, when all oscillators have nearly identical natural frequencies, the triadic motif shown in Fig. 1 admits open sets of coupling functions for which the dynamics converges to a stable triplet-locked state that cannot be decomposed into combinations of pair-

* eddie.nijholt@gmail.com
† tiago@icmc.usp.br
‡ wolfrum@wias-berlin.de
§ sagnik.chakraborty@slu.edu
¶ istvan.kiss@slu.edu
** kurths@pik-potsdam.de

wise phase-locking relations. Remarkably, despite the fact that all oscillator pairs are themselves frequency-resonant, pairwise phase synchronization does not occur. We call this phenomenon hyperlocking: a form of synchronization sustained exclusively by higher-order interactions, for which coherence is achieved only through a genuine triadic phase relation.

We develop a theory, based on phase reduction and normal form transformations, that explains how this behavior emerges. The framework is constructive: it allows us to design coupling functions that realize desired hypernetwork states, thus offering control over collective dynamics. We support our theoretical predictions with both numerical simulations and chemical experiments. Our findings show that triadic interactions can give rise to fundamentally new dynamical ~~regimes~~mechanism, and suggest that they may play a key role in shaping collective behavior in real-world systems.

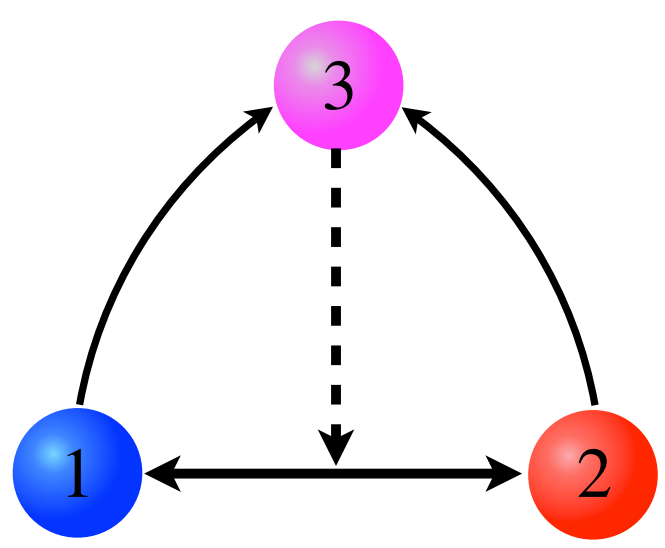

FIG. 1. **Triadic hypernetwork motif.** Pairwise interactions (black arrows) and non-pairwise interaction: node 3 influences the coupling between nodes 1 and 2 (dashed arrow). Together, this establishes a hyperedge including all three nodes and acting on nodes 1 and 2 in a symmetric way.

TRIADIC NETWORK MOTIF

The interaction structure we consider is depicted in Fig. 1. It corresponds to a system of coupled oscillators of the form

$$\begin{aligned} \dot{z}_1 &= f_1(z_1) + \alpha g(z_3) h(z_2, z_1) \\ \dot{z}_2 &= f_2(z_2) + \alpha g(z_3) h(z_1, z_2) \\ \dot{z}_3 &= f_3(z_3) + \alpha h(z_1, z_3) + \alpha h(z_2, z_3)\,, \end{aligned} \tag{1}$$

with z_j, $j \in \{1, 2, 3\}$, being the dynamical variables for the oscillators and $\alpha \in \mathbb{R}$ the coupling strength. For $\alpha = 0$, i.e., without coupling, each oscillator is assumed to have a stable periodic oscillation with natural frequency ω_j. Eq. (1) captures the higher-order nature of Fig. 1 in the following way. The dyadic coupling function h establishes a classical network structure of pairwise interactions between the nodes, indicated in Fig. 1 by the black arrows. The hypernetwork nature of our motif is induced by the dashed arrow corresponding to the function g. It indicates that node 3 influences the dyadic couplings between nodes 1 and 2, and g, together with h, induces a hyperlink among the three nodes. As we will show below, in a situation close to full resonance, i.e., when each frequency is distinct but is near a common frequency, this type of higher-order coupling can give rise to hyperlocking.

Effective Frequencies and Hyperlocking

Since the oscillators have stable periodic orbits, the oscillatory behavior persists for small couplings $0 < \alpha \ll 1$, however, their frequencies can change because of the interaction and develop an effective frequency. To obtain the effective frequency for each oscillator, we first introduce the phase $\phi_i(t) = \phi_i(z_i(t)) \in S^1 = \mathbb{R}/2\pi\mathbb{Z}$ and the unwrapped phase $\hat{\phi}_i(t) \in \mathbb{R}$. The effective frequency is then given by

$$\Omega_i = \lim_{t\to\infty} \frac{1}{t} \hat{\phi}_i(t).$$

Clearly, it depends on the natural frequencies ω_j, on the network structure, and on the coupling functions.

Pairwise and triplet locking. In networks, one expects to have a phase locking between pairs of connected nodes whenever their natural frequencies are near a resonance $n\omega_i + m\omega_j \approx 0$ for some integers n, m. This means that the effective frequencies exactly satisfy

$$n\Omega_i + m\Omega_j = 0, \tag{2}$$

which corresponds to the fact that $n\hat{\phi}_i(t) + m\hat{\phi}_j(t)$ stay bounded. Similarly, one can define triplet locking [28], where for three integers (ℓ, n, m) we have

$$\ell\Omega_i + n\Omega_j + m\Omega_k = 0. \tag{3}$$

Classical theory shows that close to a triplet resonance of the natural frequencies

$$\ell\omega_1 + n\omega_2 + m\omega_3 = \mathcal{O}(\alpha^2)$$

a generic pairwise coupling typically induces a corresponding triplet locking (3). Such a triplet resonance typically comes without any additional pairwise resonances and, hence, no simultaneous pairwise locking is expected. This is completely different in the case of full resonance, where all three frequencies are nearly identical. In this case, all pairs of frequencies are resonant and pairwise locking (2), where one or several of the pairs $(\ell, n), (n, m), (m, \ell)$ are equal to $(1, -1)$, could emerge and, at the same time, non-trivial triplets as, e.g., $(\ell, n, m) = (1, 1, -2)$ are resonant and could induce a corresponding triplet locking (3). Note that at full resonance, triplet locking may also arise as a direct consequence of two pairwise lockings. For a triplet with effective frequencies $(\Omega_1, \Omega_2, \Omega_3)$, if $\Omega_1 = \Omega_2$ and $\Omega_2 = \Omega_3$, then the triplet relation

$$\Omega_1 + \Omega_2 - 2\Omega_3 = 0$$

is satisfied. In fact, two pairwise resonances

$$n\Omega_1 = m\Omega_2 \quad \text{and} \quad r\Omega_2 = s\Omega_3$$

imply not only a third pairwise resonance $nr\Omega_1 = sm\Omega_3$, but also a triplet resonance

$$n\Omega_1 + s\Omega_3 = (m + r)\Omega_2.$$

Here, the triplet relations are *induced* by pairs.

We define *hyperlocking* as a situation where, close to a full resonance, a stable triplet locking arises, while none of the available pairwise resonances induces a corresponding pairwise locking. This means in particular that a triplet locking arising at a triplet resonance we do not designate as hyperlocking, since pairwise locking is excluded *a priori* by the frequency detuning.

Our main result is that, in the hypernetwork motif, robust hyperlocking occurs for proper choices of the coupling function. The synergy between the coupling function and triadic motif generates a collective phase relation that cannot be decomposed into pairwise locking.

Networks versus triadic motif. Notice that if we set $g(z) = 1$, this is equivalent to removing the dashed link from node 3, in Fig. 1. This reduces the triadic motif to a classical network of pairwise coupled oscillators. Then, classical synchronization theory shows that if the natural frequencies are not close to full resonance, but instead satisfy a triplet relation such as $\omega_1 - 2\omega_3 + \omega_2 \approx 0$, then triplet locking may occur [28, 29]. However, near full resonance $\omega_1 \approx \omega_2 \approx \omega_3$, triplet locking appears as a byproduct of pairwise locking generically. Indeed, for networks, the effective dynamics is dominated by pairwise interactions, as we show in Supplementary Note I. This is a fundamental feature of networks: when interactions are pairwise, and nodes are near full resonance, the effective reduced dynamics remain pairwise, generically.

HYPERLOCKING NEAR A HOPF BIFURCATION

We choose Stuart–Landau oscillators

$$f_j(z) = (\lambda + i\omega_j)z - |z|^2 z, \tag{4}$$

where z is a complex number, with $j = 1, 2, 3$, and $\lambda \in \mathbb{R}$ provides the radius of a stable periodic orbit $z_j(t) = \sqrt{\lambda} e^{i\omega_j t}$ with frequency ω_j emerges for positive values of λ. We can achieve the hyperlocking behavior by choosing linear coupling functions

$$h(z, w) = w - \bar{z} \tag{5}$$
$$g(u) = u\,.$$

We choose similar frequencies $\omega_1 = 0.95$, $\omega_2 = 1.05$ and $\omega_3 = 0.98$ together with a small coupling α and solve system (1) numerically using Julia [30][31]. In Fig. 2(a), we show the time traces of the real parts $\mathrm{Re}(z_j(t))$. These coupling functions g and h were not chosen ad hoc. They are designed from our normal form approach so that our triadic network displays hyperlocking.

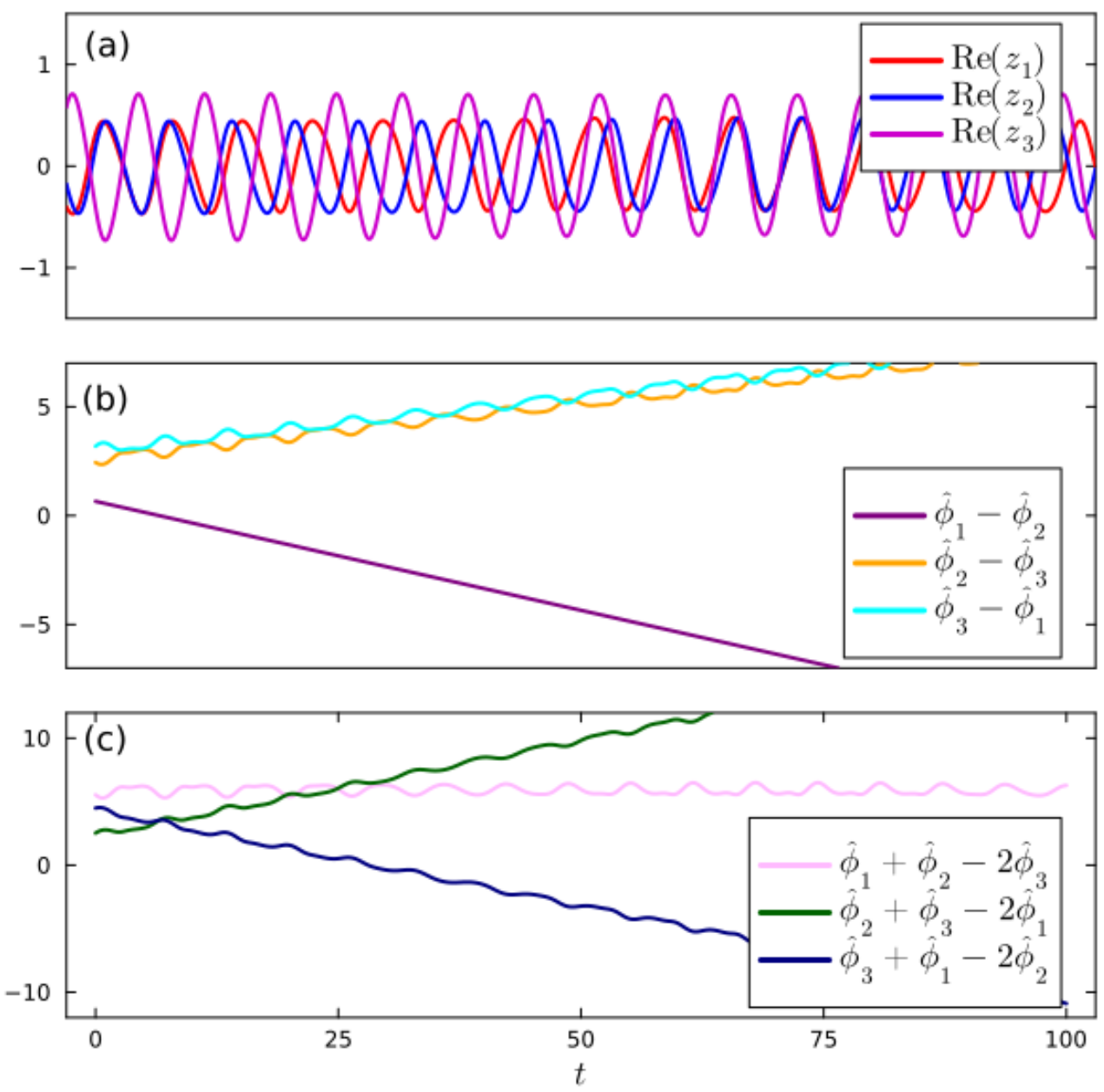

FIG. 2. **Dynamics of the triadic network.** Numerical solution of system (1)–(5). Panel (a) shows the time traces of the real parts of z_1, z_2, and z_3. Panel (b) shows the pairwise phase differences and reveals that no phase locking (where the pairwise phase difference is bounded) is observed. Panel (c) shows the triplet phase differences. The triplet combination $\phi_1+\phi_2-2\phi_3$ (blue) remains bounded, indicating the existence of a hyperlocking between this triplet of phases. Parameters: $\omega_1 = 0.95, \omega_2 = 1.05, \omega_3 = 0.98, \lambda = 0.2, \alpha = 0.15$.

We observe nearly sinusoidal oscillations with amplitude $\sqrt{\lambda} \approx 0.55$. To see the interaction of their phases, we perform a polar decomposition $z_i(t) = r_j e^{i\phi_j}$ and obtain the unwrapped phases $\hat{\phi}_j$. In Fig. 2(b), we plot the pairwise phase differences between $\hat{\phi}_i - \hat{\phi}_j$ for all pairs $i \neq j$ and notice that the differences grow in time, indicating the absence of pairwise frequency locking. However, as seen in Fig. 2(c), there is hyperlocking for $(\ell, m, n) = (1, 1, -2)$, since the corresponding triplet phase $\hat{\phi}_1 + \hat{\phi}_2 - 2\hat{\phi}_3$ stays bounded.

To explore this hyperlocking further, we fix the parameters as before but vary the frequency ω_3. For each value of ω_3, we calculate numerically the effective frequencies Ω_1, Ω_2, and Ω_3 shown in Fig. 3(a). In panel (b), we show the triplet $\Omega_1 + \Omega_2 - 2\Omega_3$. In the vicinity of the triplet resonance of the natural frequencies at $\omega_3 = (\omega_1 + \omega_2)/2$, we observe a large plateau where the triadic coupling induces stable hyperlocking without any pairwise locking. The pairwise locking at the two points of pairwise resonance $\omega_3 = \omega_{1,2}$ is much less pronounced.

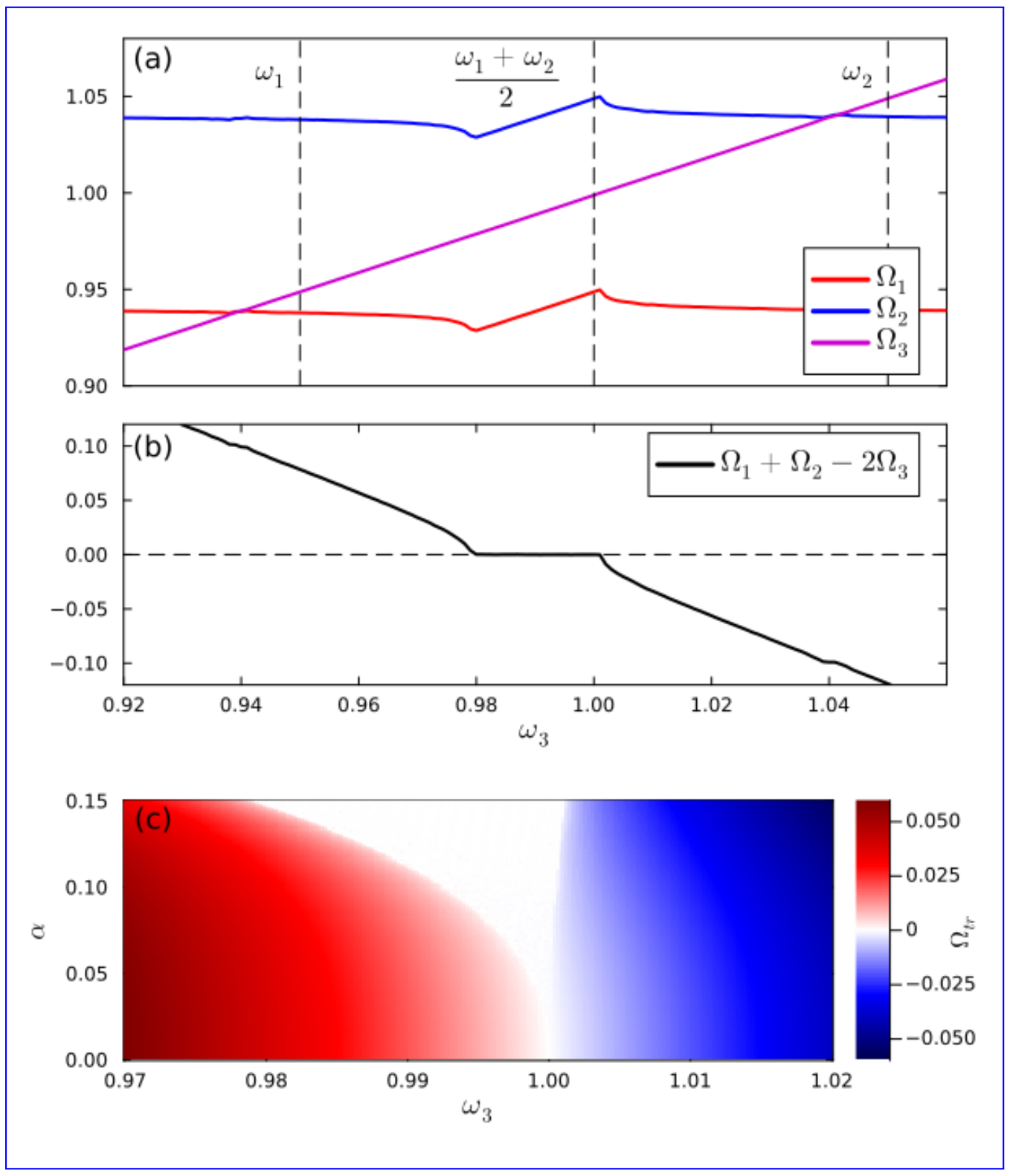

FIG. 3. **Hyperlocking between triplets of phases.** Panel (a) shows effective frequencies for varying natural frequency ω_3. Panel (b) shows the effective frequency triplet $\Omega_{\rm tr} = \Omega_1+\Omega_2-2\Omega_3$ for varying ω_3. The plateau at zero reveals the hyperlocking emergent from the triadic interaction. Panel (c) shows the quadratic locking cone that arises when also the coupling strength α is varied (white region where $\Omega_{\rm tr}=0$). All other parameters are the same as in Fig. 2.

A THEORY FOR EMERGENT HYPERLOCKING

To explain the hyperlocking behaviour, we develop a normal form theory for the triadic network as shown in Supplementary Note II. We give a short outline of our treatment. For this triadic motif with the specific choice of the coupling functions (5), all coupling terms appearing in first order are *non-resonant*. This means that they represent small, rapid fluctuations that do not reveal the dynamics on larger time scales. To treat the higher-order terms, we employ techniques from normal form theory. The idea is to use successive coordinate transformations on the z-variables to remove as many terms as possible from Eq. (1), before performing the phase reduction. We show that non-resonant terms can be removed.

The coordinate transformations employed in our theoretical analysis are perturbations of the identity map. This proximity ensures that any locking relations observed in the transformed coordinates also manifest in the original system. Therefore, the analytical derivation based on transformed coordinates captures the actual dynamics of the original system.

After the normal form transformations, we obtain the phase equations to leading order

$$\dot\phi_1 = \omega_1^{\rm eff} - \alpha_{\rm eff}^2 \sin(2\phi_3-\phi_1-\phi_2+\gamma_1) \quad (6)$$
$$\dot\phi_2 = \omega_2^{\rm eff} - \alpha_{\rm eff}^2 \sin(2\phi_3-\phi_1-\phi_2+\gamma_1) \quad (7)$$
$$\dot\phi_3 = \omega_3 \quad (8)$$

where $\omega_i^{\rm eff}$ and $\alpha_{\rm eff}$ are the effective frequency and coupling functions. They are obtained in Supplementary Note II. We obtain that $\omega_i^{\rm eff} = \omega_i + O(\alpha^2\lambda)$, and $\alpha_{\rm eff} \approx \alpha\sqrt{\lambda/\omega_3}$. Notice that, to leading order, no pairwise synchronization is present. For instance, consider $\theta=\phi_1-\phi_2$ and a frequency gap $\omega_1-\omega_2 = O(\alpha^2)$. In this case, the higher-order corrections to Eqs. (6)-(8) are of order $O(\alpha^3)$, which means that for small coupling, no pairwise locking will be present. Because to obtain a pairwise locking state, the frequency gap needs to be of the same order as the coupling strength. We provide the full argument in Supplementary Note III. On the other hand, we can obtain a stable triplet locking even in the absence of a pairwise locking. The hyperlocked state is hyperbolic and stable under small smooth perturbations.

Introducing the triplet phase

$$\varphi = \phi_1+\phi_2-2\phi_3,$$

we get the Adler equation

$$\dot\varphi = \Delta - 2\alpha_{\rm eff}^2 \sin(\gamma_1-\varphi)$$

where $\Delta = \omega_1^{\rm eff}+\omega_2^{\rm eff}-2\omega_3$. In Supplementary Note III, we determine the frequency locking properties of such Adler equations. Hence, our analytical approach explains the numerically observed stable hyperlocking and, in particular, reproduces the plateau observed in Fig. 3(b) and the quadratic locking cone in Fig. 3(c).

Note that the effective phase dynamics in Eqs. (6)–(8) contains only terms of order $O(\alpha^2)$ and no Kuramoto-type pairwise sinusoidal coupling of the form $\sin(\phi_j-\phi_k+\gamma)$. This absence is a direct consequence of the underlying triadic interaction motif. This behavior is fundamentally different from that of the *classical Kuramoto model*, which arises as the phase reduction of Stuart–Landau oscillators with nearly identical natural frequencies and *pairwise linear coupling*. As shown in the Supplementary Note I, for purely pairwise linear coupling, the resonant pairwise terms persist under normal-form transformations and cannot be eliminated by nonlinear coordinate changes. Consequently, when pairs of oscillators are frequency-resonant, pairwise phase locking dominates the dynamics and is generically stable. In this situation, any apparent triplet locking then occurs only as a secondary consequence of pairwise locking.

SYNERGY BETWEEN TRIADIC MOTIF AND NONLINEAR COUPLING

The emergent dynamics of the triadic motif reflect a synergy between the hypernetwork structure and the

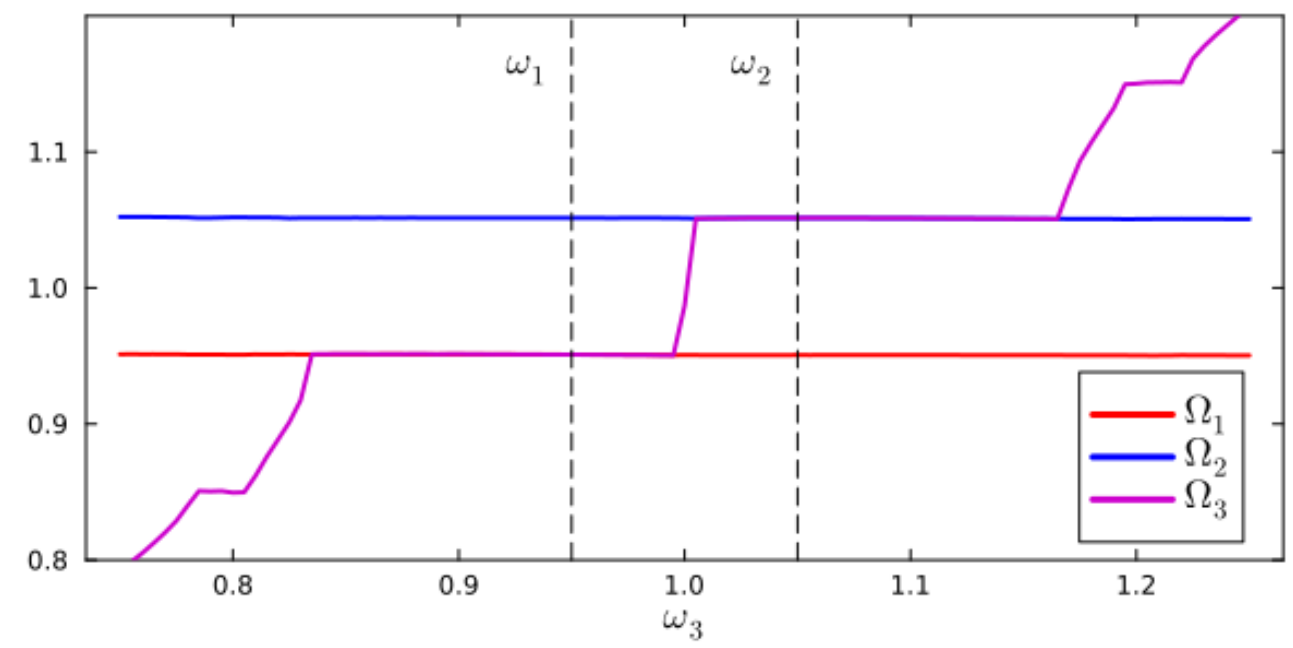

FIG. 4. **Different coupling functions may lead to pairwise locking.** Using in system (1)–(5) the coupling function $h(z,w) = w - z$, pairwise locking becomes predominant. Around the corresponding resonances of the natural frequencies there are two large regions with $\Omega_1 = \Omega_3$ and $\Omega_2 = \Omega_3$. Triplet locking $2\Omega_1 = \Omega_2 + \Omega_3$ and ~~$2\Omega_3 = \Omega_1 + \Omega_3$~~ $2\Omega_3 = \Omega_1 + \Omega_2$ occurs only in two small regions close to the corresponding triplet resonances. Parameters as in Fig.2.

nonlinearities of the coupling functions. For the class of coupling functions we consider, it is striking that, despite near full resonance, no pairwise synchronization occurs. A different choice of the coupling function, however, can restore stable pairwise locking: Fig. 4 shows that with the coupling function given as $h(z,w) = w - z$, the dynamics are dominated by pairwise interactions resulting in pairwise locking as can be seen by the plateaus where $\Omega_3 = \Omega_2$ and ~~$\Omega_3 = \Omega_2$~~$\Omega_3 = \Omega_1$, which means that the hyperlocking effect disappears. Note that the coupling still has the same nonlinear triadic structure, only the specific monomials in the interaction are different. But this already results in new resonant terms that are essential for the effective phase dynamics.

HYPERLOCKING WITH ELECTROCHEMICAL OSCILLATOR NETWORK

We confirmed the emergence of triplet locking in the absence of pairwise phase locking through experiments involving three oscillatory electrochemical reactions coupled via nonlinear feedback and delay. The experimental setup, shown in Fig. 5, consists of a multichannel potentiostat connected to the three nickel working electrodes in sulfuric acid electrolyte. At a constant circuit potential with an added external resistance, the electrochemical dissolution of nickel exhibits periodic electrode potential (E_k) oscillations reflecting the variations of the chemical composition of the metal-electrolyte interface.

We consider three such nickel wires that constitute the three nodes of the network. The network coupling is introduced using a real-time controller that implements

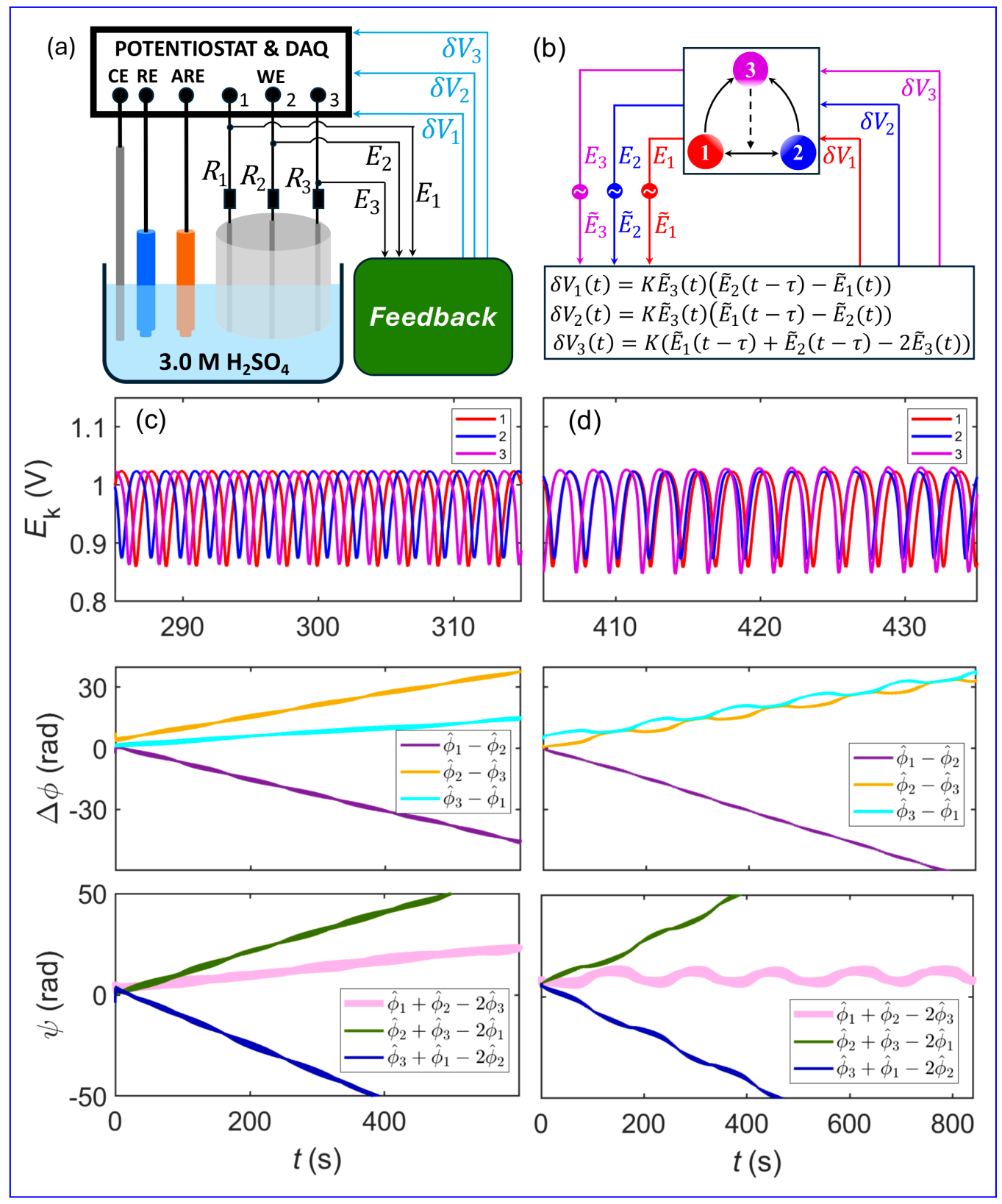

FIG. 5. **Hyperlocking in a triadic hypernetwork motif with electrochemical oscillators.** (a) Experimental setup. CE, RE, ARE, and WE are the counter, reference, auxiliary reference, and the working electrodes. R_k, δV_k, and E_k denote the individual resistors, the circuit potential perturbations, and the measured electrode potentials, respectively. (b) Schematic illustration of the feedback mechanism. K and τ are the feedback strength and delay, and $\tilde{E}_k$ are the rescaled electrode potentials used in the feedback scheme. (c-d) (Top to bottom) Electrode potential time series, evolution of pairwise and triplet phase differences with time, respectively. (c) Uncoupled oscillations. $K = 0$ V^{-1}. (d) Hyperlocking in the presence of feedback. K = -0.2 V^{-1} and $\tau = 1.1$ s.

an external feedback as

$$\delta V_1(t) = K\tilde{E}_3(t)(\tilde{E}_2(t-\tau) - \tilde{E}_1(t))$$
$$\delta V_2(t) = K\tilde{E}_3(t)(\tilde{E}_1(t-\tau) - \tilde{E}_2(t))$$
$$\delta V_3(t) = K((\tilde{E}_1(t-\tau) - \tilde{E}_3(t)) + (\tilde{E}_2(t-\tau) - \tilde{E}_3(t)))$$

where δV_k and $\tilde{E}_k(t)$ are the circuit potential perturbation applied via the feedback mechanism and the offset corrected and rescaled electrode potential of the kth electrode, respectively, K is the coupling strength, τ is a time delay. The circuit potentials of oscillators 1 and 2 depend nonlinearly on the states of all three oscillators. Further experimental details are given in Supplementary Note IV. The feedback scheme represents the hypernetwork: Nodes 1 and 2 are delay-coupled, and the coupling strength is modulated by node 3. Node 3 is coupled to nodes 1 and 2 through a delayed difference mechanism.

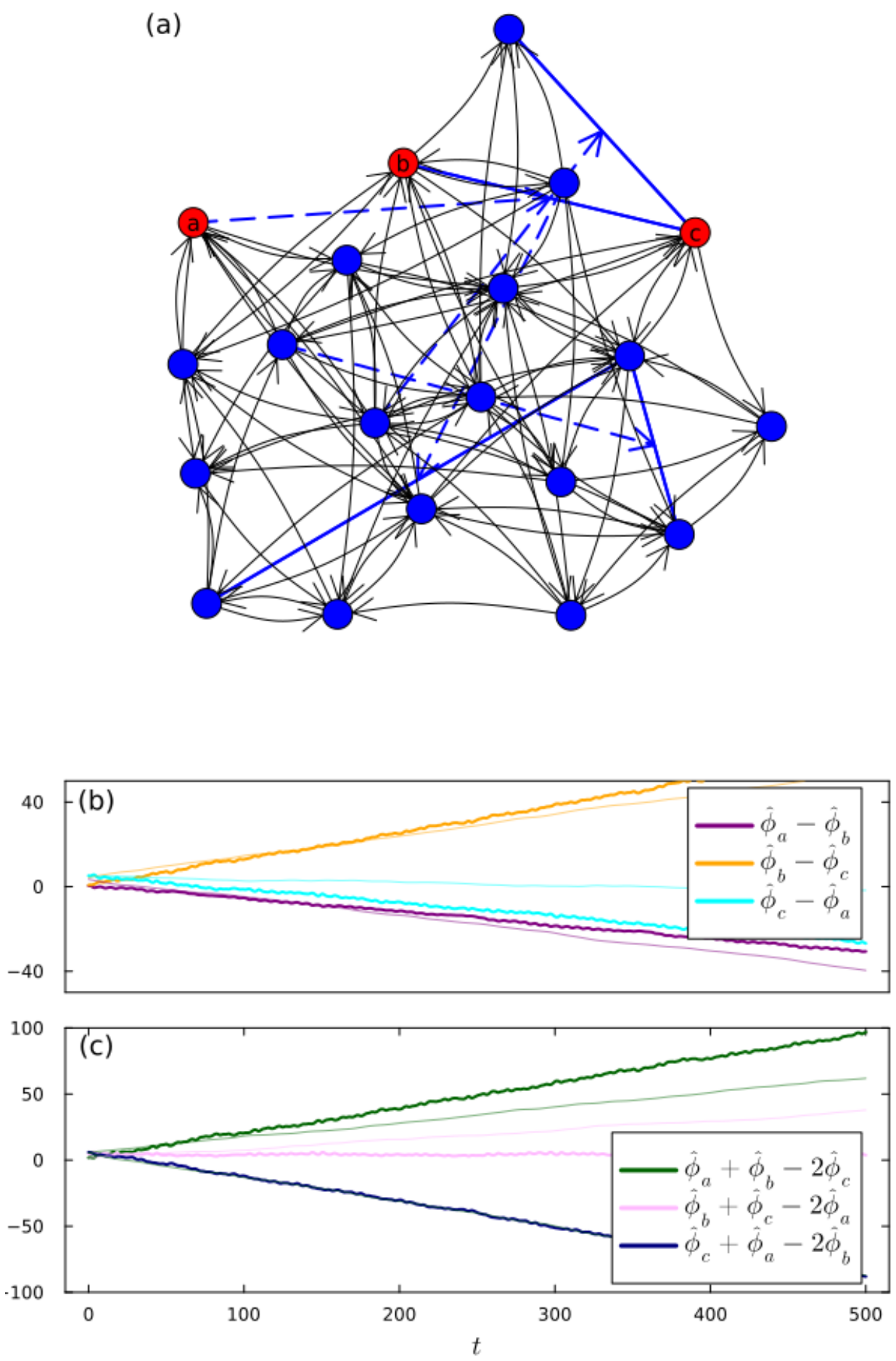

FIG. 6. **Hyperlocking in a larger network containing triadic motifs.** (a) Random hypernetwork with directed pairwise links (black arrows) and triadic coupling (blue), where a third node influences (dashed blue arrow) the interaction between a pair (blue arrows). The triplet of red nodes has frequencies $\omega_{a,b,c}$ close to full resonance. All other natural frequencies are chosen randomly. The dynamics of system (9) ~~show~~ shows a hyperlocking of this triplet for sufficiently strong triplet coupling strength α (dark colors), while for $\alpha = 0$, i.e., only pairwise but no triplet coupling, no hyperlocking is present (light colors). (b) Pairwise frequency differences of the resonant triplet show no pairwise locking among these nodes. (c) Triplet phase differences show hyperlocking when triplet coupling is present. Parameters: $\alpha = 1.5$, $\beta = 1.0$, $\lambda = 0.3$.

Note that without a delay term in the coupling, the system would settle into a pairwise, in-phase synchronized state, leading to regular phase locking [32]. In this sense, introducing delay in the experiments serves a role similar to introducing conjugate coupling in the model, namely, preventing stable pairwise synchronization. We adopted this form of coupling because delays are known to disrupt synchronization in coupled networks [33]. The delayed nonlinear feedback applied here does not produce obvious synchronization patterns, for example, one or multiple phase-cluster states [34] due to the modulation of the coupling strength.

The triadic structure arises from the form of the coupling itself, while the delay enables access to a phase regime in which pairwise synchronization is destabilized and hyperlocking can emerge. In the weak-coupling regime, delays act by adjusting the effective phase of the interaction, playing a role akin to conjugate coupling[35].

To describe the nature of the phase dynamics, the phases $\hat{\phi}_k$, from the electrode potential oscillations, were extracted using the Hilbert transform technique [36]. The natural frequency of each oscillator was adjusted with a set of resistors so that ω_2 - ω_1 = 12 mHz, ω_3 - ω_1 = 3 mHz and ω_2 - ω_3 = 9 mHz. The natural frequencies $\omega_1 \approx 448$ mHz, $\omega_2 \approx 460$ mHz, and $\omega_3 \approx 451$ mHz create a condition for triplet resonance, as there is a small detuning for ω_1 - $2\omega_3$ + ω_2 = 6 mHz. Fig. 5 (c) demonstrates that without coupling ($K = 0$ V^{-1}), the electrode potentials of the three nodes oscillate independently and the pairwise and triplet phase differences (including φ) vary linearly with time. In this state, there is no pairwise or triplet phase locking.

Fig. 5 (d) shows the results in the presence of feedback with K = -0.2 V^{-1} and $\tau = 1.1$ s (half the mean period of oscillations of the three nodes). No pairwise synchrony was observed: $\hat{\phi}_2 - \hat{\phi}_3$ and $\hat{\phi}_3 - \hat{\phi}_1$ show oscillations and drift with time. The triplet phase difference φ exhibited phase locking (hyperlocking, with 0 mHz triplet frequency difference) with a small amplitude oscillation, while the other two possible triplet phase differences drifted with time. The experiments demonstrate that hyperlocking is a robust phenomenon in a hypernetwork motif where pairwise locking is not possible due to the nature of the nonlinear interactions.

The electrochemical dynamics are chemically complex, involving multiple reaction pathways such as direct metal oxidation, the formation of oxide films, oxygen evolution, and the adsorption of chemical species within a diffuse double layer [37]. The experimental observation of hyperlocking, therefore, indicates that the phenomenon is robust to intrinsic nonlinearities, measurement noise, and slow experimental drifts. The results are also robust to the chosen coupling delay: Hyperlocking was also observed with delays set to $2-4\%$ different than $T/2$ as shown in Supplementary Note IV. Although coupling was implemented using an external feedback loop, this setup effectively mimics realistic situations in which interaction strengths depend nonlinearly on the system state.

OUTLOOK: HYPERLOCKING FOR OTHER NONLINEAR OSCILLATORS AND LARGER NETWORKS

So far, we have investigated hyperlocking in a triadic hypernetwork motif of three coupled oscillators near a

Hopf bifurcation. In Supplementary Note V, we demonstrate numerically that a similar coupling structure can also induce hyperlocking in a system of three nonlinear van der Pol oscillators.

The hyperlocking mechanism uncovered here is not restricted to the specific triadic motif. Rather, this motif can serve as a fundamental building block for more complex networks that contain such triadic structures and exhibit hyperlocking behavior. In the Supplementary Note III, we demonstrate how our analytical theory can be applied similarly to an extended motif containing the triadic motif. Below, we give an example of a hypernetwork consisting of a set $\mathcal{N} = \{1, \ldots, n\}$ of oscillating nodes with randomly chosen directed pairwise links $j \to k$, given by a directed graph $\mathcal{P}$, and an additional randomly selected collection $\mathcal{T}$ of triplet hyperlinks $k \to \{j, \ell\}$, where node k influences a pairwise interaction between nodes j and ℓ. For $\mathcal{T}$, we admit only triplets of nodes with no pairwise links in $\mathcal{P}$. The dynamics are given by

$$\dot{z}_j = f_j(z_j) + \frac{\beta}{|\mathcal{P}|} \sum_{(j \to k) \in \mathcal{P}} q(z_k, z_j) + \frac{\alpha}{|\mathcal{T}|} \sum_{(k \to \{j,\ell\}) \in \mathcal{T}} g(z_k) h(z_\ell, z_j) \qquad (9)$$

with Stuart–Landau oscillators f_j as in (4) and triplet coupling functions g and h from (5) with coupling strength α. For the pairwise coupling, we choose $q(z, w) = z - w$ with coupling strength β.

For the numerical simulations shown in Fig. 6, we have first generated a random hypernetwork with $n = 20$ nodes, 93 directed links, and four triplet hyperedges, see panel (a). After choosing random natural frequencies equally distributed in the interval $[0.7, 1.3]$, we have slightly adapted the frequencies $\omega_{a,b,c}$ of the nodes in one of the hyperlinks to achieve a situation sufficiently close to a full resonance among these nodes. In panels (b) and (c), we plotted the pairwise and triplet phase combinations among these nodes with (dark colors) and without (light colors) the triplet coupling. The results clearly show that sufficiently strong triplet coupling induces hyperlocking among these nodes.

These cases illustrate that hyperlocking can emerge in extended systems through the embedding of triadic interactions, highlighting the broader relevance of this phenomenon for the dynamics of higher-order networks.

CONCLUSION

We have shown that hyperlocking emerges as a synergy between a triadic coupling structure and a nonlinear coupling function. We demonstrate that hyperlocking is a robust dynamical phenomenon that appears in a triadic motif of coupled Stuart-Landau oscillators, where it can be treated analytically. The experiments on electrochemical oscillators demonstrated that hyperlocking is robust under realistic conditions, including experimental noise and slow parameter drifts caused by electrode deterioration. Although the oscillations originate from a Hopf bifurcation [38], the observed waveforms indicate that the system operates far from the bifurcation point, as illustrated in Fig. 5, showing that hyperlocking is not a near-bifurcation effect. Consistently, we also observe hyperlocking in other dynamical settings, including networks of coupled van der Pol oscillators.

There is extensive literature showing that higher-order interactions can modify known dynamical phenomena in oscillator networks, such as chimeras [39], synchronization thresholds [40], and solitary states [41], often by shifting parameter regimes or stability boundaries relative to purely pairwise coupling. Our result is qualitatively different. In the present system, when the coupling reduces to a purely pairwise network, no triplet locking appears. The hyperlocking reported here arises only from triadic interactions, rather than as a higher-order correction to effects of pairwise coupling. Our results also highlight that the boundary between network and hypernetwork dynamics is not clear-cut. Only the interplay between network structure, coupling functions, and resonances decides the true nature of the emergent dynamical interactions in the system.

Our analytical approach to this hyperlocking consists of performing a nonlinear change of coordinates that preserves dynamical phenomena. In these new coordinates, pairwise resonant terms are eliminated, while triadic interactions survive and organize the dynamics. Therefore, hyperlocking cannot be reduced to a sum of pairwise interactions. In certain scenarios, a higher-order interaction can be expressed as a combination of pairwise interactions, see for instance [42]. In this case, the same vector field can be represented in different bases. An instructive example is when the coupling function is linear, and the higher-order interaction can be expressed as a sum of pairwise interactions [43]. In this sense, higher-order or pairwise terms sometimes provide just alternative representations of the same system.

We introduce a nonlinear change of variables that maps the system to a dynamically equivalent vector field, retaining higher-order terms only when they are dynamically relevant. This coordinate transformation changes the vector field and allows us to demonstrate that hyperlocking is a dynamical effect induced by higher-order interactions, rather than a reparametrization of pairwise coupling. Triadic motifs emerge as flexible building blocks of collective behavior, capable of supporting synchronization regimes that cannot be sustained by networks with pairwise interactions alone, and point to a broader class of higher-order dynamical phenomena awaiting exploration.

Hyperlocking is a general synchronization mechanism in networks with higher-order interactions, in which certain expected frequency lockings, although formally possible, are not dynamically selected, and coherence emerges only through higher-order relations. Surpris-

ingly, we show that hyperlocking is negligible in generic networks with purely pairwise coupling. Triadic networks, therefore, constitute minimal building blocks in which this phenomenon can occur. These findings demonstrate that, in networks with higher-order interactions, synchronization cannot in general be reduced to the analysis of pairwise correlations and is intrinsically multibody in nature. This opens several directions for future research, including extending the analysis to other bifurcation scenarios that generate periodic oscillations in the isolated system, examining whether similar effects arise in systems with symmetric multibody interactions, and exploring the possibility of hyperlocking between clusters of oscillators rather than between individual units.

Acknowledgements. We thank Bob Rink, Chris Bick, Dima Turaev, and Deniz Eroglu for enlightening discussions. TP was supported in part by FAPESP Grant No. 2023/13706-0 and CNPq 312287/2021-6. EN was supported by FAPESP Grant No. 2024/00930-2. TP and EN were partially supported by Serrapilheira Institute (Grant No. 270 Serra-1709-16124) and warmly thank the hospitality of the Weierstrass Institute during their multiple visits. IZK and SC acknowledge financial support from the National Science Foundation Grant No 1900011 and NSF-NIGMS 1R01GM157609 grant. MW acknowledges the hospitality during his research stay at ICMC-USP. TP thanks the Humboldt Foundation for support through a Bessel Fellowship.

Data Availability. ~~The data supporting the findings of this study consist of (i) numerical simulation outputs and (ii) experimental time series from electrochemical oscillator networks. Processed datasets underlying the figures and main analyses, as well as the raw experimental time series, are available from the corresponding author upon reasonable request.All data necessary to reproduce the main results are included in the paper and its Supplementary Information.~~ Data for numerical simulations are provided at https://doi.org/10.5281/zenodo.19821152. Experimental source data for all figures are deposited at https://doi.org/QQQ/zenodo.QQQ.

Code Availability. The numerical simulations and analysis routines used in this study were implemented in ~~custom Python~~Julia, using the package 'DifferentialEquations.jl' [31]. The computational notebooks can be downloaded from https:/~~MATLAB/Juliascripts.The code required to reproduce the main numerical results and figures is available upon reasonable request~~/doi.org/10.5281/zenodo.19821152.

[1] T. Stankovski, T. Pereira, P. V. McClintock, and A. Stefanovska, Coupling functions: universal insights into dynamical interaction mechanisms, Reviews of Modern Physics **89**, 045001 (2017).

[2] M. Golubitsky, I. Stewart, and A. Török, Patterns of synchrony in coupled cell networks with multiple arrows, SIAM Journal on Applied Dynamical Systems **4**, 78–100 (2005).

[3] T. Pereira, D. Eroglu, G. B. Bagci, U. Tirnakli, and H. J. Jensen, Connectivity-driven coherence in complex networks, Phys. Rev. Lett. **110**, 234103 (2013).

[4] O. E. Omel'chenko and M. Wolfrum, Nonuniversal transitions to synchrony in the Sakaguchi-Kuramoto model, Phys. Rev. Lett. **109**, 164101 (2012).

[5] E. Nijholt, T. Pereira, F. C. Queiroz, and D. Turaev, Chaotic behavior in diffusively coupled systems, Communications in Mathematical Physics **401**, 2715 (2023).

[6] G. Bianconi, *Higher-order networks* (Cambridge University Press, 2021).

[7] C. Bick, E. Gross, H. A. Harrington, and M. T. Schaub, What are higher-order networks?, SIAM review **65**, 686 (2023).

[8] A. P. Millán, H. Sun, L. Giambagli, R. Muolo, T. Carletti, J. J. Torres, F. Radicchi, J. Kurths, and G. Bianconi, Topology shapes dynamics of higher-order networks, Nature Physics **21**, 353–361 (2025).

[9] R. Tönjes, C. E. Fiore, and T. Pereira, Coherence resonance in influencer networks, Nature Communications **12**, 72 (2021).

[10] L. Gallo, R. Muolo, L. V. Gambuzza, V. Latora, M. Frasca, and T. Carletti, Synchronization induced by directed higher-order interactions, Communications Physics **5**, 263 (2022).

[11] S. Rosenthal, J. M. Shine, and M. Breakspear, Triadic interactions and functional information dynamics in brain networks, Nature Communications **14**, 1469 (2023).

[12] J. Grilli, G. Barabás, M. J. Michalska-Smith, and S. Allesina, Higher-order interactions stabilize dynamics in competitive network models, Nature **548**, 210 (2017).

[13] J. Zscheischler, S. Westra, B. Van Den Hurk, S. I. Seneviratne, P. J. Ward, A. Pitman, A. AghaKouchak, D. N. Bresch, M. Leonard, T. Wahl, and X. Zhang, Future climate risk from compound events, Nature Reviews Earth & Environment **1**, 333 (2020).

[14] T. Liu, N. Boers, R. Winkelmann, J. Kurths, B. Goswami, N. Wunderling, and J. F. Donges, Network interaction among tipping elements in the earth system, Nature Climate Change **13**, 67 (2023).

[15] I. Iacopini, G. Petri, A. Barrat, and V. Latora, Simplicial models of social contagion, Nature Communications **10**, 2485 (2019).

[16] F. Battiston, G. Cencetti, I. Iacopini, V. Latora, M. Lucas, A. Patania, J.-G. Young, and G. Petri, The physics of higher-order interactions in complex systems, Nature Physics **17**, 1093 (2021).

[17] D. Liuzza, F. Della Rossa, F. L. Iudice, and P. De Lellis, Nonlinear average consensus over circulating directed hypergraphs, IEEE Control Systems Letters (2025).

[18] A. Santoro, F. Battiston, G. Petri, and E. Amico, Higher-order organization of multivariate time series, Nature Physics **19**, 221–229 (2023).

[19] F. Malizia, A. Corso, L. V. Gambuzza, G. Russo, V. Latora, and M. Frasca, Reconstructing higher-order interactions in coupled dynamical systems, Nature Communications **15**, 5184 (2024).

[20] E. Nijholt, J. L. Ocampo-Espindola, D. Eroglu, I. Z. Kiss, and T. Pereira, Emergent hypernetworks in weakly coupled oscillators, Nature communications **13**, 4849 (2022).

[21] G. Ferraz de Arruda, A. Aleta, and Y. Moreno, Contagion dynamics on higher-order networks, Nature Reviews Physics **6**, 468 (2024).

[22] Y. Zhang, M. Lucas, and F. Battiston, Higher-order interactions shape collective dynamics differently in hypergraphs and simplicial complexes, Nature Communications **14**, 1605 (2023).

[23] Y. Zhang, P. S. Skardal, F. Battiston, G. Petri, and M. Lucas, Deeper but smaller: Higher-order interactions increase linear stability but shrink basins, Science Advances **10**, eado8049 (2024).

[24] S. Rakshit, B. K. Bera, E. M. Bollt, and D. Ghosh, Intralayer synchronization in evolving multiplex hypernetworks: Analytical approach, SIAM Journal on Applied Dynamical Systems **19**, 918 (2020).

[25] P. S. Skardal and A. Arenas, Higher order interactions in complex networks of phase oscillators promote abrupt synchronization switching, Communications Physics **3**, 218 (2020).

[26] A. Civilini, O. Sadekar, F. Battiston, J. Gómez-Gardeñes, and V. Latora, Explosive cooperation in social dilemmas on higher-order networks, Phys. Rev. Lett. **132**, 167401 (2024).

[27] S. von der Gracht, E. Nijholt, and B. Rink, Higher-order interactions lead to 'reluctant' synchrony breaking, Proceedings of the Royal Society A: **480**, 20230945 (2024).

[28] B. Kralemann, A. Pikovsky, and M. Rosenblum, Detecting triplet locking by triplet synchronization indices, Phys. Rev. E **87**, 052904 (2013).

[29] J. Jia, Z. Song, W. Liu, J. Kurths, and J. Xiao, Experimental study of the triplet synchronization of coupled nonidentical mechanical metronomes, Scientific Reports **5**, 17008 (2015).

[30] C. Rackauckas and Q. Nie, Differentialequations.jl – a performant and feature-rich ecosystem for solving differential equations in julia, Journal of Open Research Software 10.5334/jors.151 (2017).

[31] C. Rackauckas and Q. Nie, Differentialequations.jl–a performant and feature-rich ecosystem for solving differential equations in julia, Journal of Open Research Software **5**, 15 (2017).

[32] J. L. Ocampo-Espindola, I. Z. Kiss, C. Bick, and K. C. A. Wedgwood, Strong coupling yields abrupt synchronization transitions in coupled oscillators, Phys. Rev. Res. **6**, 033328 (2024).

[33] Y. Zhai, I. Z. Kiss, and J. L. Hudson, Control of complex dynamics with time-delayed feedback in populations of chemical oscillators: Desynchronization and clustering, Ind. Eng. Chem. Res. **47**, 3502 (2008).

[34] C. Bick, M. Sebek, and I. Z. Kiss, Robust weak chimeras in oscillator networks with delayed linear and quadratic interactions, Phys. Rev. Lett. **119**, 168301 (2017).

[35] C.-U. Choe, T. Dahms, P. Hövel, and E. Schöll, Controlling synchrony by delay coupling in networks: From in-phase to splay and cluster states, Phys. Rev. E **81**, 025205 (2010).

[36] A. Pikovsky, M. Rosenblum, and J. Kurths, *Synchronization: A Universal Concept in Nonlinear Sciences* (Cambridge University Press, 2001).

[37] D. Haim, O. Lev, L. M. Pismen, and M. Sheintuch, Modeling periodic and chaotic dynamics in anodic nickel dissolution, J. Phys. Chem. **96**, 2676 (1992).

[38] I. Z. Kiss, Z. Kazsu, and V. Gáspár, Tracking unstable steady states and periodic orbits of oscillatory and chaotic electrochemical systems using delayed feedback control., Chaos **16**, 033109 (2006).

[39] S. Kundu and D. Ghosh, Higher-order interactions promote chimera states, Physical Review E **105**, L042202 (2022).

[40] Y. Zhang, V. Latora, and A. E. Motter, Unified treatment of synchronization patterns in generalized networks with higher-order, multilayer, and temporal interactions, Communications Physics **4**, 195 (2021).

[41] V. V. Semenov, S. Dutta, S. Boccaletti, C. I. Del Genio, S. Jalan, and A. Zakharova, Solitary states in spiking oscillators with higher-order interactions, Physical Review E **112**, 034302 (2025).

[42] L. Neuhäuser, M. Scholkemper, F. Tudisco, and M. T. Schaub, Learning the effective order of a hypergraph dynamical system, Science Advances **10**, eadh4053 (2024).

[43] R. Lambiotte, M. Rosvall, and I. Scholtes, From networks to optimal higher-order models of complex systems, Nature Physics **15**, 313–320 (2019).
